# The Cranial Osteology and Feeding Ecology of the Metriorhynchid Crocodylomorph Genera *Dakosaurus* and *Plesiosuchus* from the Late Jurassic of Europe

**DOI:** 10.1371/journal.pone.0044985

**Published:** 2012-09-18

**Authors:** Mark T. Young, Stephen L. Brusatte, Marco Brandalise de Andrade, Julia B. Desojo, Brian L. Beatty, Lorna Steel, Marta S. Fernández, Manabu Sakamoto, Jose Ignacio Ruiz-Omeñaca, Rainer R. Schoch

**Affiliations:** 1 School of Geosciences, University of Edinburgh, Edinburgh, United Kingdom; 2 Division of Paleontology, American Museum of Natural History, New York City, New York, United States of America; 3 Department of Earth and Environmental Sciences, Columbia University, New York City, New York, United States of America; 4 Departamento de Paleontologia e Estratigrafia, Universidade Federal do Rio Grande do Sul, Porto Alegre, Brazil; 5 División de Paleontología, Museo Argentino de Ciencias Naturales ‘Bernardino Rivadavia’, Buenos Aires, Argentina; 6 Department of Anatomy, New York College of Osteopathic Medicine, Old Westbury, New York, United States of America; 7 Department of Earth Sciences, Natural History Museum, London, United Kingdom; 8 Departamento Paleontología de Vertebrados, Museo de La Plata, La Plata, Argentina; 9 School of Earth Sciences, University of Bristol, Bristol, United Kingdom; 10 Museo del Jurásico de Asturias (MUJA), Colunga, Spain; 11 Departamento de Geología, Universidad de Oviedo, Oviedo, Spain; 12 Staatliches Museum für Naturkunde, Stuttgart, Germany; Ludwig-Maximilians-Universität München, Germany

## Abstract

**Background:**

*Dakosaurus* and *Plesiosuchus* are characteristic genera of aquatic, large-bodied, macrophagous metriorhynchid crocodylomorphs. Recent studies show that these genera were apex predators in marine ecosystems during the latter part of the Late Jurassic, with robust skulls and strong bite forces optimized for feeding on large prey.

**Methodology/Principal Findings:**

Here we present comprehensive osteological descriptions and systematic revisions of the type species of both genera, and in doing so we resurrect the genus *Plesiosuchus* for the species *Dakosaurus manselii*. Both species are diagnosed with numerous autapomorphies. *Dakosaurus maximus* has premaxillary ‘lateral plates’; strongly ornamented maxillae; macroziphodont dentition; tightly fitting tooth-to-tooth occlusion; and extensive macrowear on the mesial and distal margins. *Plesiosuchus manselii* is distinct in having: non-amblygnathous rostrum; long mandibular symphysis; microziphodont teeth; tooth-crown apices that lack spalled surfaces or breaks; and no evidence for occlusal wear facets. Our phylogenetic analysis finds *Dakosaurus maximus* to be the sister taxon of the South American *Dakosaurus andiniensis*, and *Plesiosuchus manselii* in a polytomy at the base of Geosaurini (the subclade of macrophagous metriorhynchids that includes *Dakosaurus*, *Geosaurus* and *Torvoneustes*).

**Conclusions/Significance:**

The sympatry of *Dakosaurus* and *Plesiosuchus* is curiously similar to North Atlantic killer whales, which have one larger ‘type’ that lacks tooth-crown breakage being sympatric with a smaller ‘type’ that has extensive crown breakage. Assuming this morphofunctional complex is indicative of diet, then *Plesiosuchus* would be a specialist feeding on other marine reptiles while *Dakosaurus* would be a generalist and possible suction-feeder. This hypothesis is supported by *Plesiosuchus manselii* having a very large optimum gape (gape at which multiple teeth come into contact with a prey-item), while *Dakosaurus maximus* possesses craniomandibular characteristics observed in extant suction-feeding odontocetes: shortened tooth-row, amblygnathous rostrum and a very short mandibular symphysis. We hypothesise that trophic specialisation enabled these two large-bodied species to coexist in the same ecosystem.

## Introduction

The evolution and diversification of metriorhynchid crocodylomorphs in the Mesozoic seas is a classic example of an evolutionary radiation in the fossil record [1], [2]. Metriorhynchids are highly aberrant compared to other crocodylomorphs (which are terrestrial or semi-aquatic), and evolved numerous adaptations to their pelagic lifestyle, including a complete loss of their osteoderm armour, hydrofoil-like forelimbs, a hypocercal tail, sclerotic ossicles and large salt glands [1], [3]–[9]. A flurry of recent morphological, systematic, and phylogenetic work on metriorhynchids is helping to understand their evolutionary radiation in great detail. Phylogenetic analyses robustly show that metriorhynchids are divided into two major subclades, Metriorhynchinae and Geosaurinae [1], [2], [9]–[14]. Functional and macroevolutionary studies indicate that these two subgroups were well suited for feeding on different prey and developed a great variety of body sizes, skull shapes, biting behaviours, and dental morphologies during their evolutionary history [1], [2], [11], [13]–[17].

One of the major metriorhynchid subclades, Geosaurinae, includes large-bodied taxa such as “Mr Leeds’ specimen” (GLAHM V972, the generic and specific name for this taxon is currently in press [2]), *Torvoneustes*, *Geosaurus*, and *Dakosaurus*, which had skulls and teeth well suited for feeding on large prey [1], [2], [11], [13], [14], [17]. There seems to have been a temporal and phylogenetic trend towards increasing super-predatory behaviour within this group, as progressively more derived and younger taxa had skulls that were better optimized for enduring strong bite forces [1], [13], [16]. Furthermore, because of the high diversity of tooth crown and serration morphologies among geosaurines [11], Young *et al*. [13] hypothesised that contemporaneous geosaurines were limiting competition through ecological specialisation and niche partitioning. Indeed, Late Jurassic marine ecosystems frequently had two sympatric geosaurine genera that were either apex-predators or second tier super-predators [1], [11], [18].

**Figure 1:**
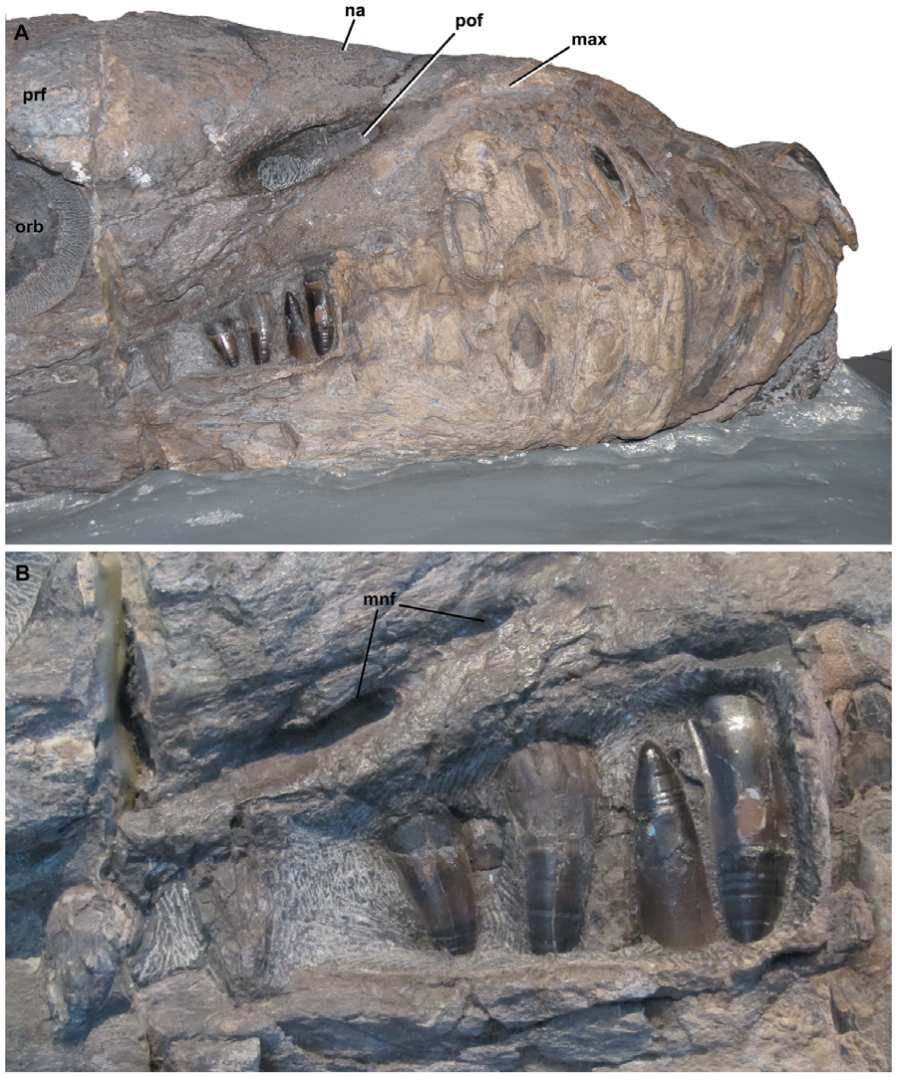
*Dakosaurus andiniensis*, referred specimen MOZ 6146P. Skull and mandible in, (A) lateral (right) view of the snout and (B) close-up on the posterior teeth, showing the interlocking dentition. Note the robust teeth and snout. Abbreviations: max, maxilla; mnf, maxillary neurovascular foramen; na, nasal; orb, orbit; pof, preorbital fenestra; prf, prefrontal.

The geosaurine genus *Dakosaurus* has been of particular interest, especially due to its unusual cranial morphology. Its skull and mandible were the most robust and powerful within Metriorhynchidae, as shown by biomechanical analyses [1], [16]. Furthermore, it had a brevirostrine and oreinirostral snout and a robust dentition, with the largest apicobasal crown lengths of any metriorhynchid and serrated carinae composed of a keel and true denticles [10], [11], [17], [19], [20] (Fig. 1). As has been hinted at in previous studies, and as we argue more fully in this monograph, it is likely that *Dakosaurus* was macrophagous: an animal that could feed upon prey items of similar body size. The larger body size of *Dakosaurus* compared to other metriorhynchids would be beneficial for such a feeding style, as it would allow this taxon to target larger prey, and would allow for a reduction in the time taken to process prey, making larger organisms more energetically feasible prey items [21].

**Figure 2:**
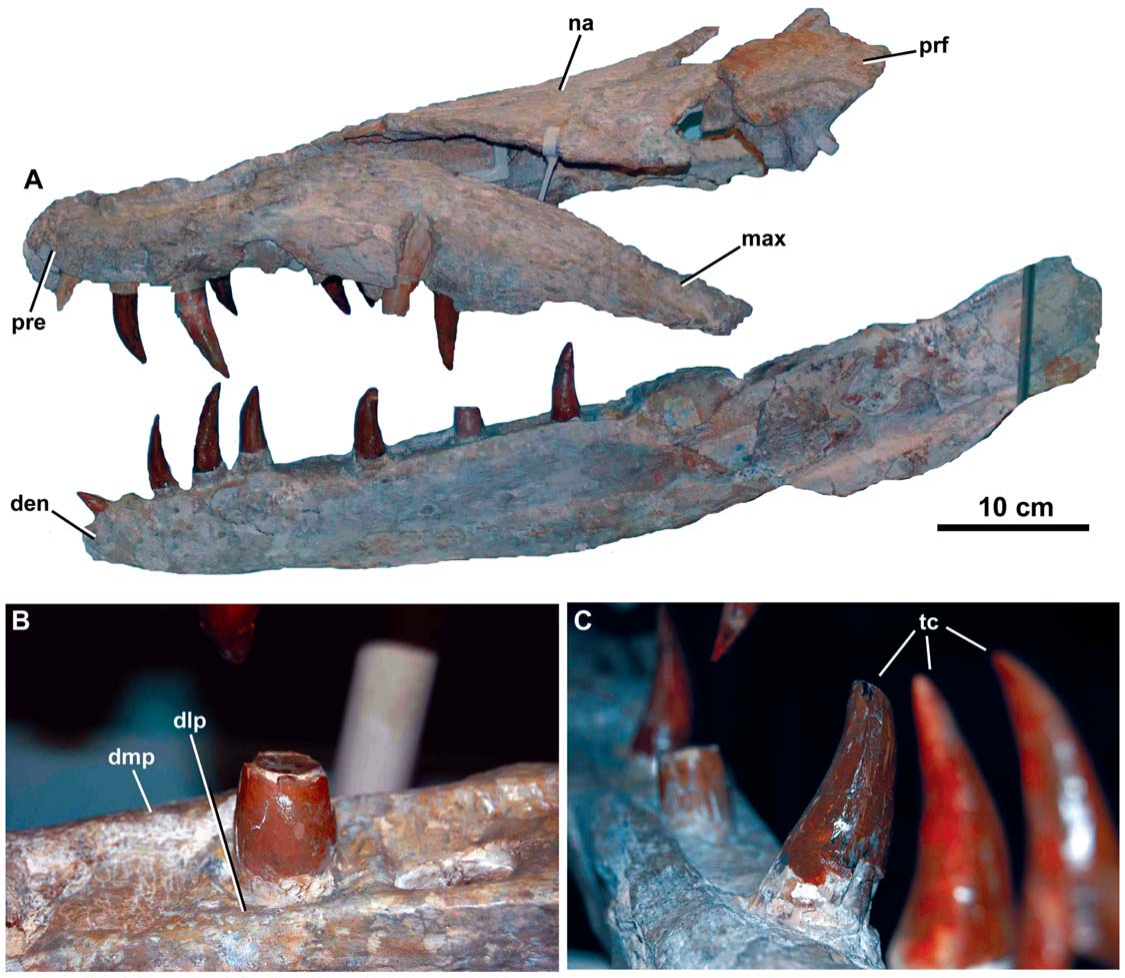
*Dakosaurus maximus*, neotype SMNS 8203. (A) General view of the skull and mandible, (B) close-up on the dentary alveoli and raised lateral and medial margins, and (C) oblique forward view of the dentary tooth row. Abbreviations: den, dentary; dlp, dentary lateral plate; dmp, dentary medial plate; max, maxilla; na, nasal; pre, premaxilla; prf, prefrontal; tc, tooth crowns.

**Figure 3:**
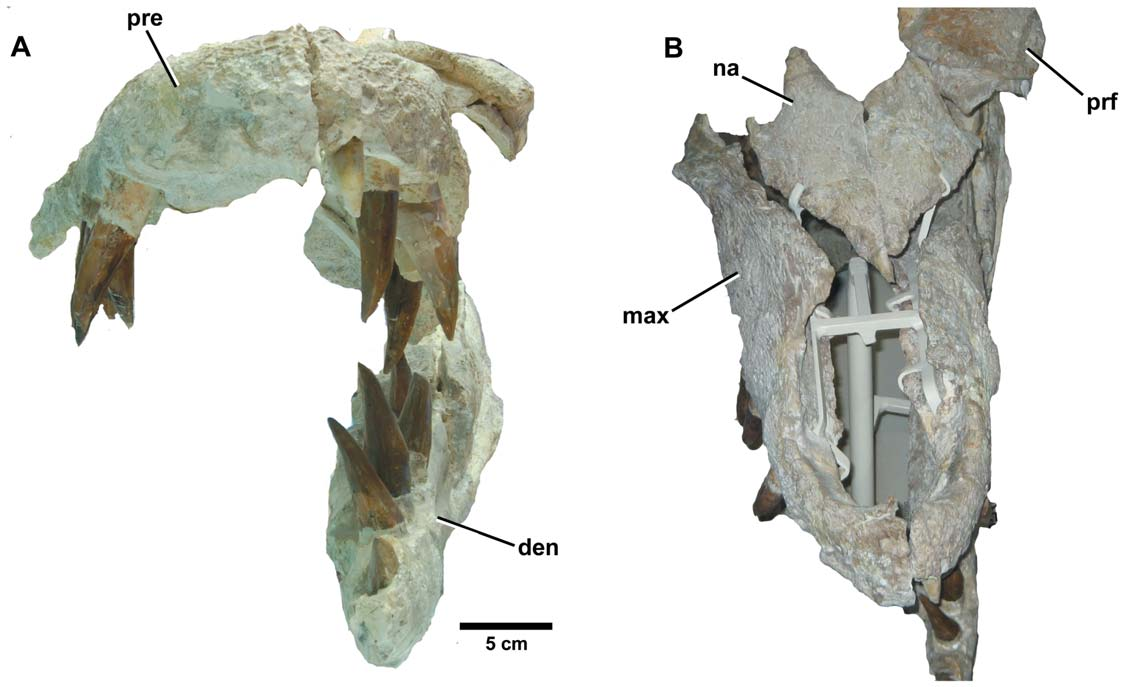
*Dakosaurus maximus*, neotype SMNS 8203. (A) Anterior view of the skull and mandible, note that level to the fourth dentary tooth the mandibular ramus deflects laterally (i.e. short symphysis), and (B) oblique dorsal view of the skull, emphasising the blunt “bullet-shaped” snout (i.e. amblygnathy). Abbreviations: den, dentary; max, maxilla; na, nasal; pre, premaxilla; prf, prefrontal.

**Figure 4:**
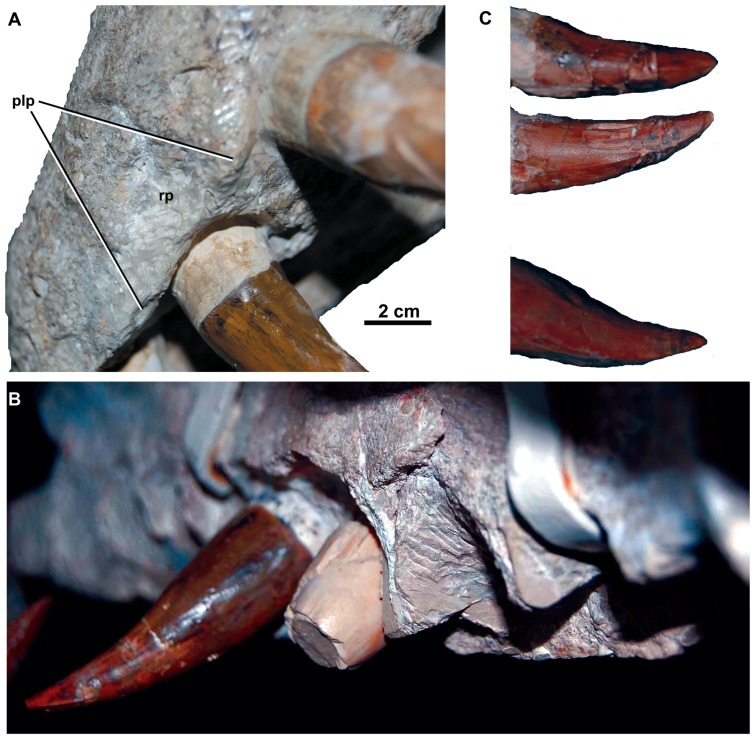
*Dakosaurus maximus*, neotype SMNS 8203. (A) Close-up on the premaxillary ‘lateral plates’, (B) close-up on the maxillary alveoli in oblique ventral view and (C) close-up on the maxillary teeth showing tooth crown wear. Abbreviations: plp, premaxillary lateral plate; rp, reception pit.

**Figure 5:**
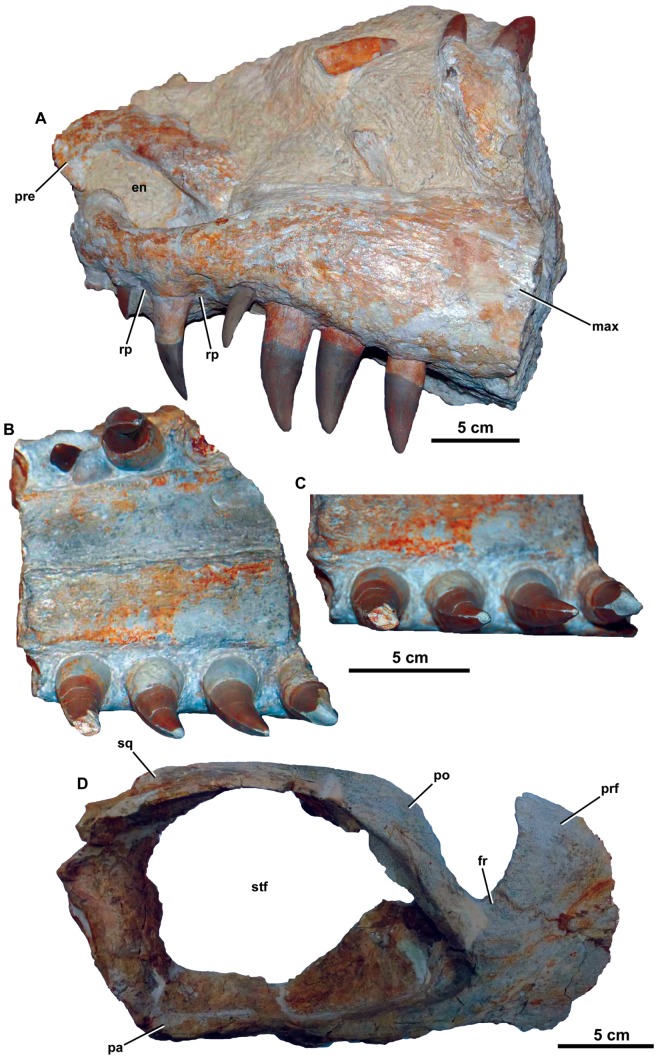
*Dakosaurus maximus*, referred specimens SMNS 10819a and SMNS 10819b. Snout (SMNS 10819a) in: (A) dorsal view, (B) ventral (palatal) view, and (C) close-up on the maxillary tooth-row in ventral view. Note that several teeth exhibit carinal wear, broken apices and spalling of the enamel at the apex. (D) Left-half of the posterior region of the skull (SMNS 10819b) in dorsal view. Abbreviations: en, external nares; fr, frontal; max, maxilla; pa, parietal; po, postorbital; pre, premaxilla; prf, prefrontal; rp, reception pit; sq, squamosal; stf, supratemporal fenetra.

**Figure 6:**
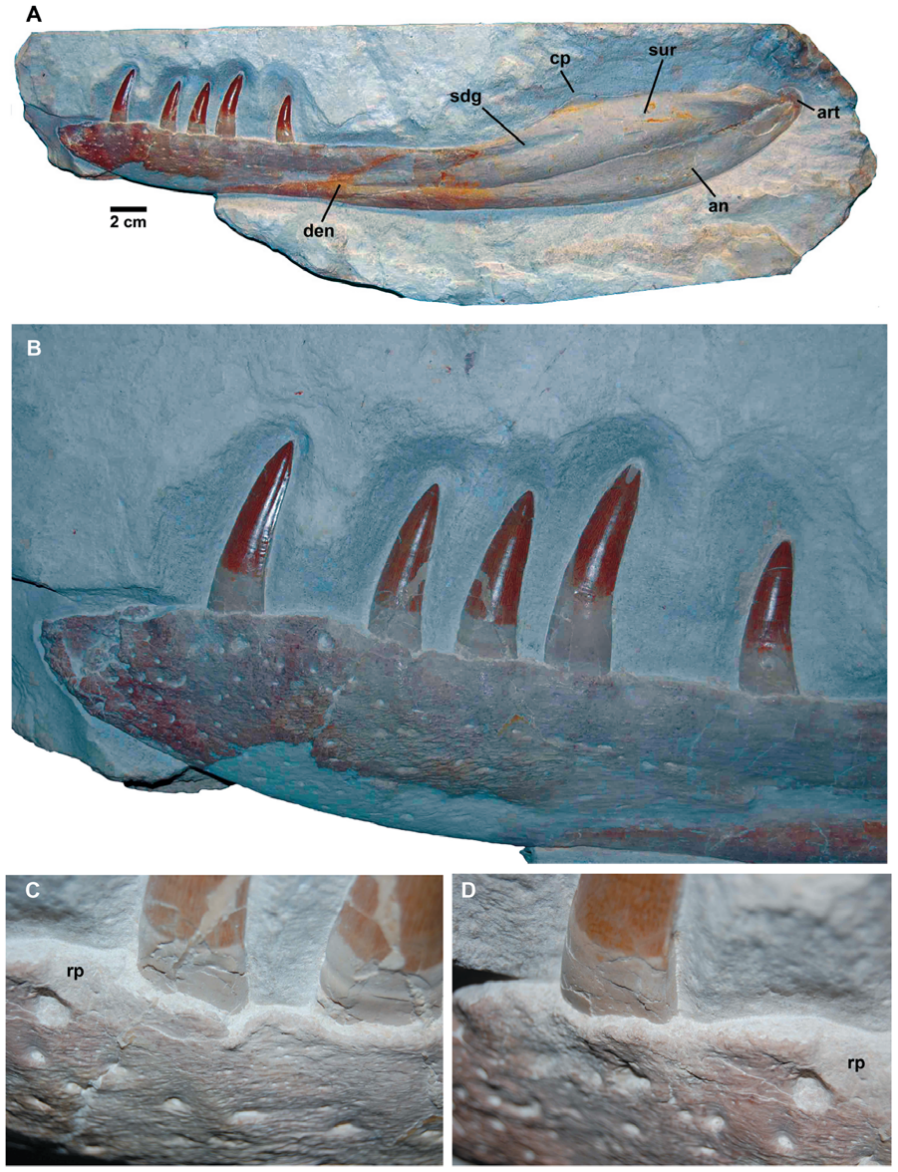
*Dakosaurus maximus*, referred specimen SMNS 82043. Left mandibular ramus in lithographic limestone in: (A) general view, (B) close-up on the anterior teeth, and (C) close-up on the dentary dorsal margin at the first preserved tooth crown, showing the various foramina and a reception pit and (D) close-up on the dentary dorsal margin, slightly further back along the tooth row. Abbreviations: an, angular; art, articular; cp, coronoid process on the surangular; den, dentary; rp, reception pit; sdg, surangulodentary groove; sur, surangular.

**Figure 7:**
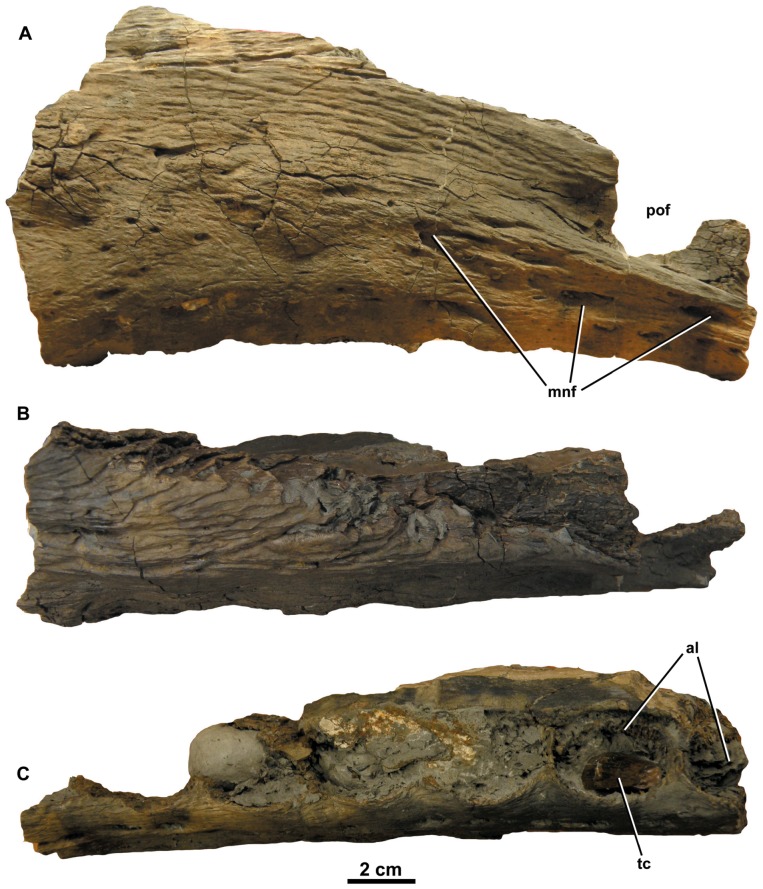
*Dakosaurus maximus*, referred specimen SMNS 56999. Left isolated maxilla in: (A) lateral view, (B) dorsal view and (C) ventral view (showing the tooth row). Abbreviations: al, alveolus; mnf, maxillary neurovascular foramen; pof, preorbital fenestra; tc, tooth crown.

**Figure 8:**
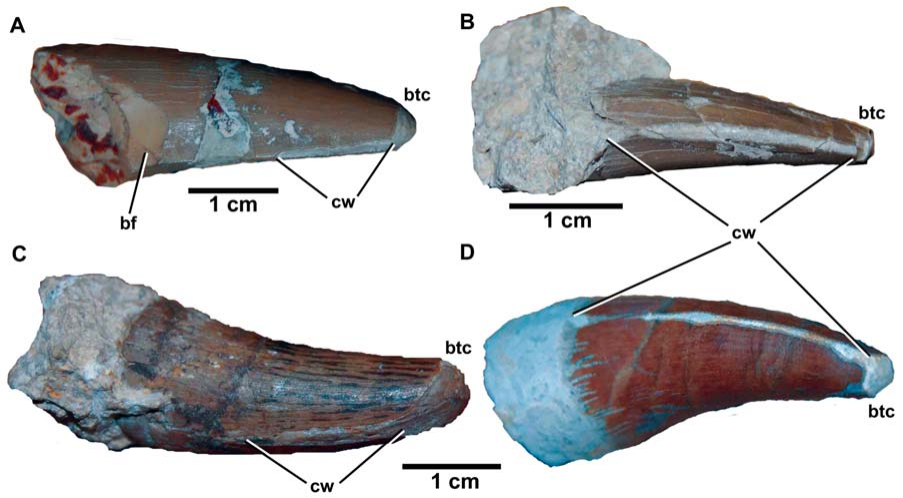
*Dakosaurus maximus*, referred specimens SMNS 91425. Numerous isolated teeth of *D. maximus* showing the occlusion wear patterns and apical breakage. Top left scale bar for (A), top right scale bar for (B), and bottom scale bar for images (C)–(D). Abbreviations: bf, basal facet; btc, broken tip; cw, carinal wear.

**Figure 9:**
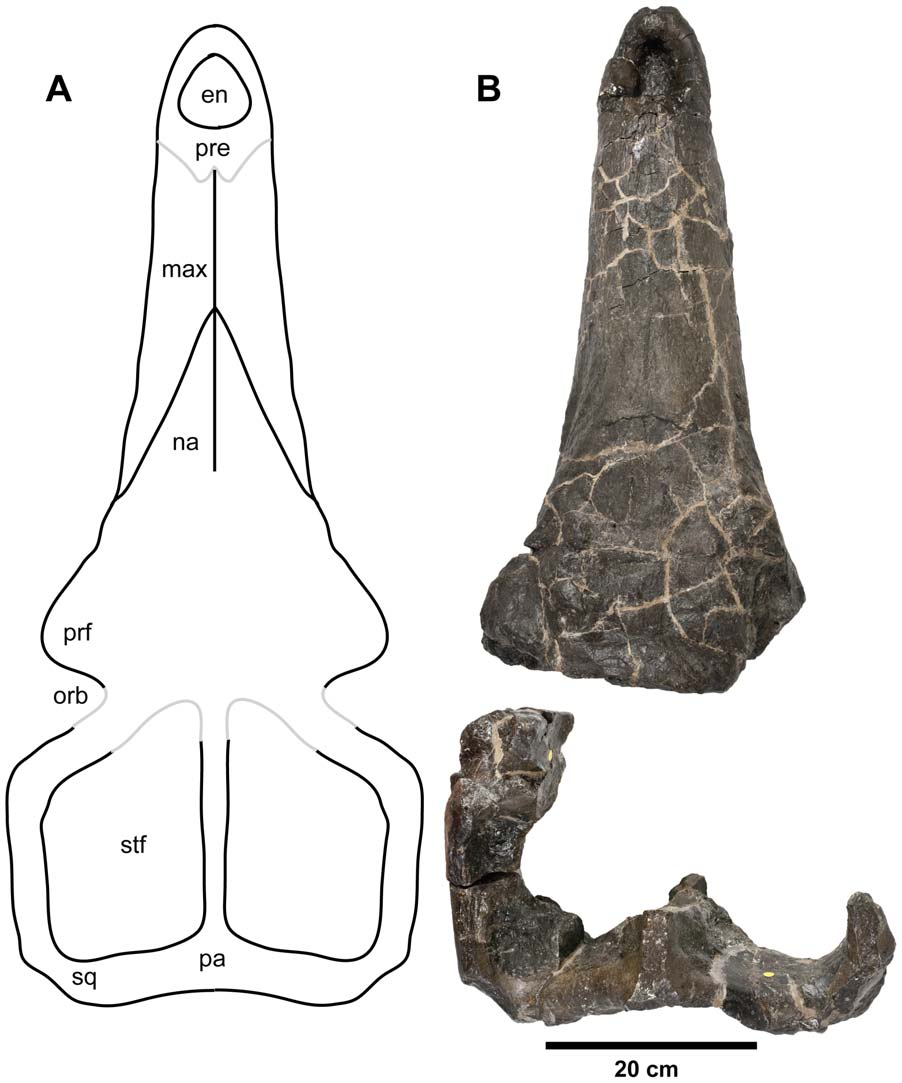
*Plesiosuchus manselii*, holotype NHMUK PV OR40103. Dorsal view of the skull, (A) hypothetical skull reconstruction (grey lines represent elements that are missing) and (B) photograph of what is preserved. Abbreviations: en, external nares; max, maxilla; na, nasal; orb, orbit; pa, parietal; pre, premaxilla; prf, prefrontal; sq, squamosal; stf, supratemporal fenestra.

**Figure 10:**
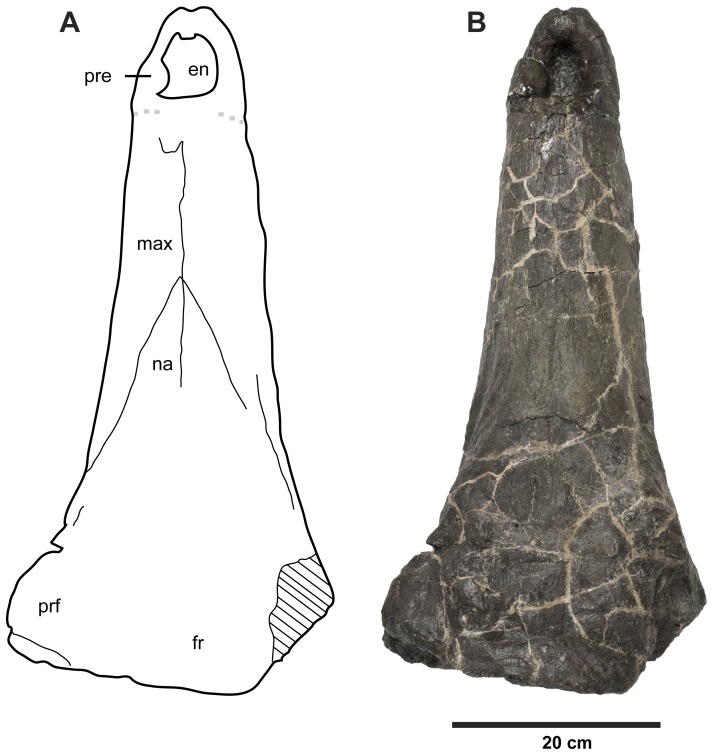
*Plesiosuchus manselii*, holotype NHMUK PV OR40103. Snout in dorsal view, (A) line drawing (grey squares represent the premaxilla-maxilla suture, the exact nature of which we are unsure) and (B) photograph. Abbreviations: en, external nares; fr, frontal; max, maxilla; na, nasal; pre, premaxilla; prf, prefrontal.

**Figure 11:**
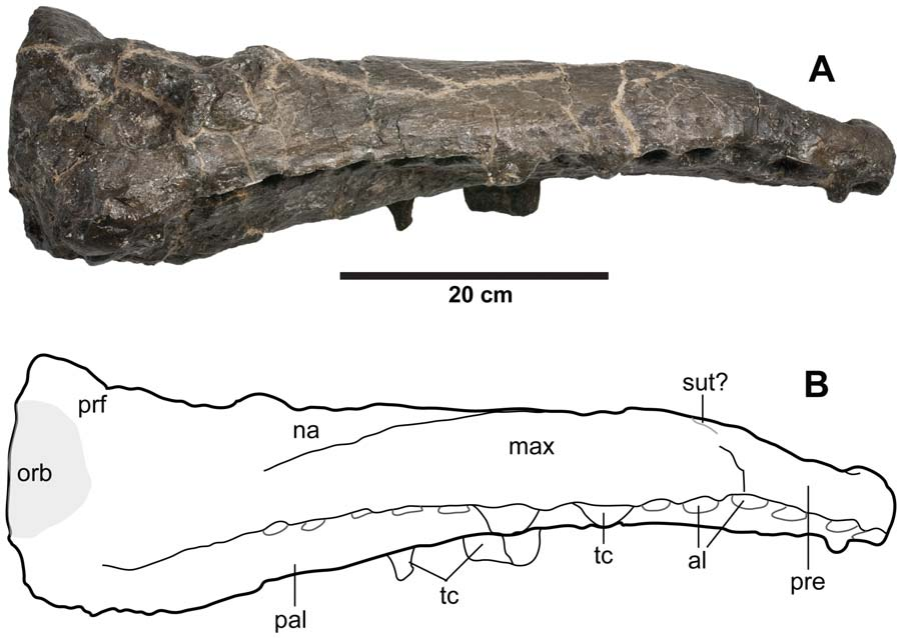
*Plesiosuchus manselii*, holotype NHMUK PV OR40103. Snout in lateral (right) view, (A) photograph and (B) line drawing (grey shaded area represents the orbital cavity, grey lines represents sutures we are unsure of). Abbreviations: al, alveolus; max, maxilla; na, nasal; orb, orbit; pal, palatine; pre, premaxilla; prf, prefrontal; sut?, suture?; tc, tooth crown.

**Figure 12:**
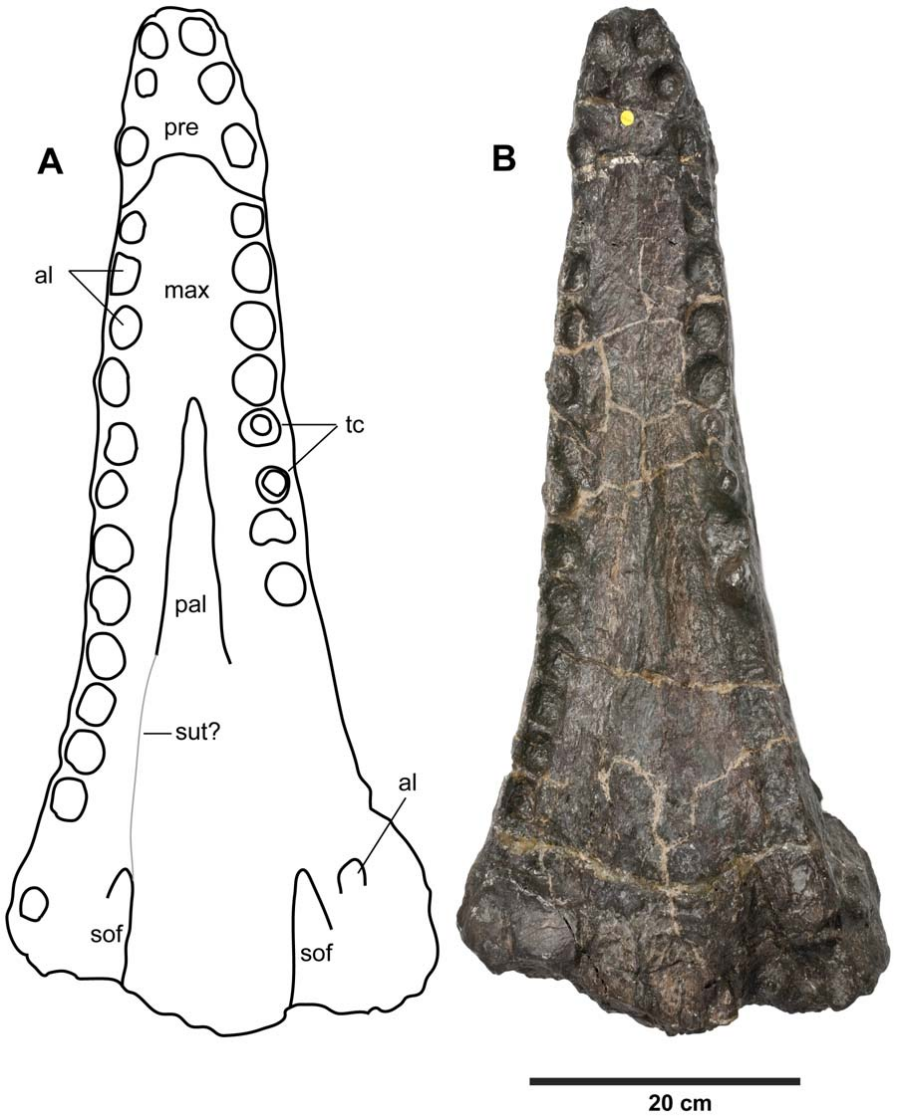
*Plesiosuchus manselii*, holotype NHMUK PV OR40103. Snout in ventral (palatal) view, (A) line drawing (grey lines represent the sutures we are unsure of) and (B) photograph. Abbreviations: al, alveolus; max, maxilla; pal, palatine; pre, premaxilla; sof, suborbital fenestra; sut?, suture?; tc, tooth crown.

**Figure 13:**
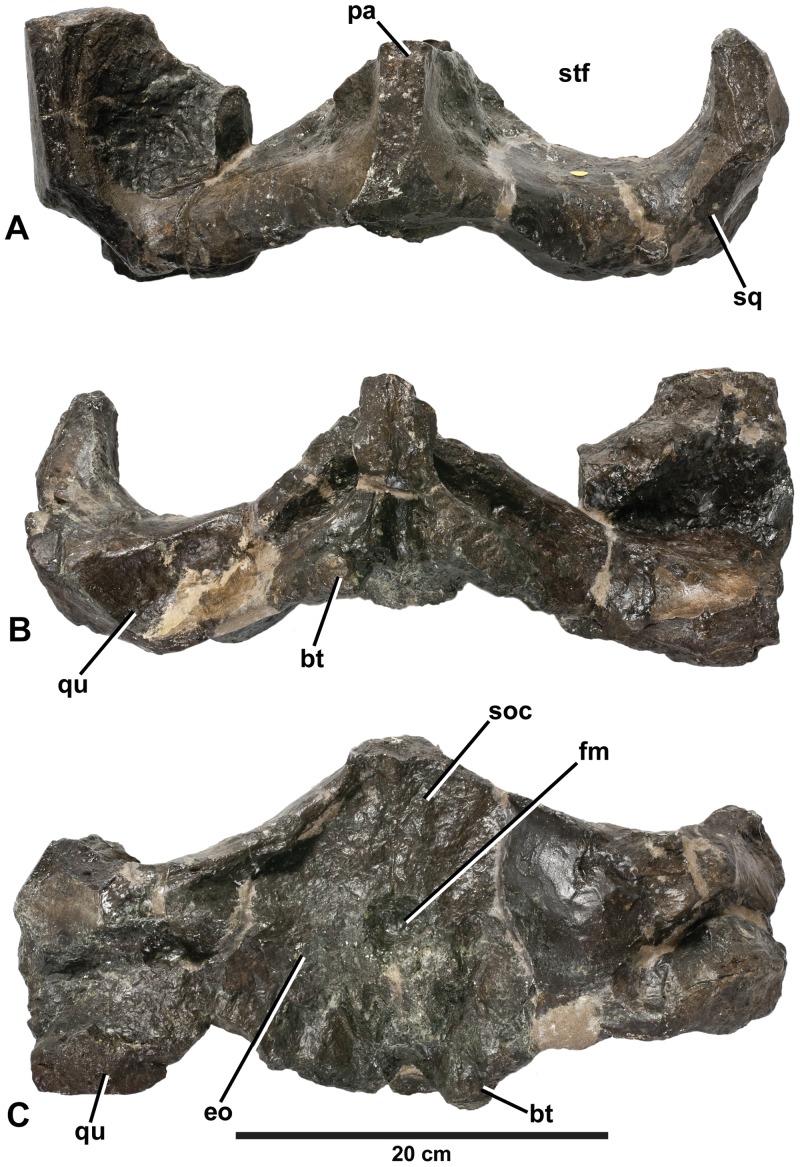
*Plesiosuchus manselii*, holotype NHMUK PV OR40103. Braincase: in (A) dorsal view, (B) ventral view and (C) occipital view. Abbreviations: bt, basal tubera; eo, exocciptial; fm, foramen magnum; pa, parietal; qu, quadrate; soc, supraoccipital; sq, squamosal; stf, supratemporal fenestra.

**Figure 14:**
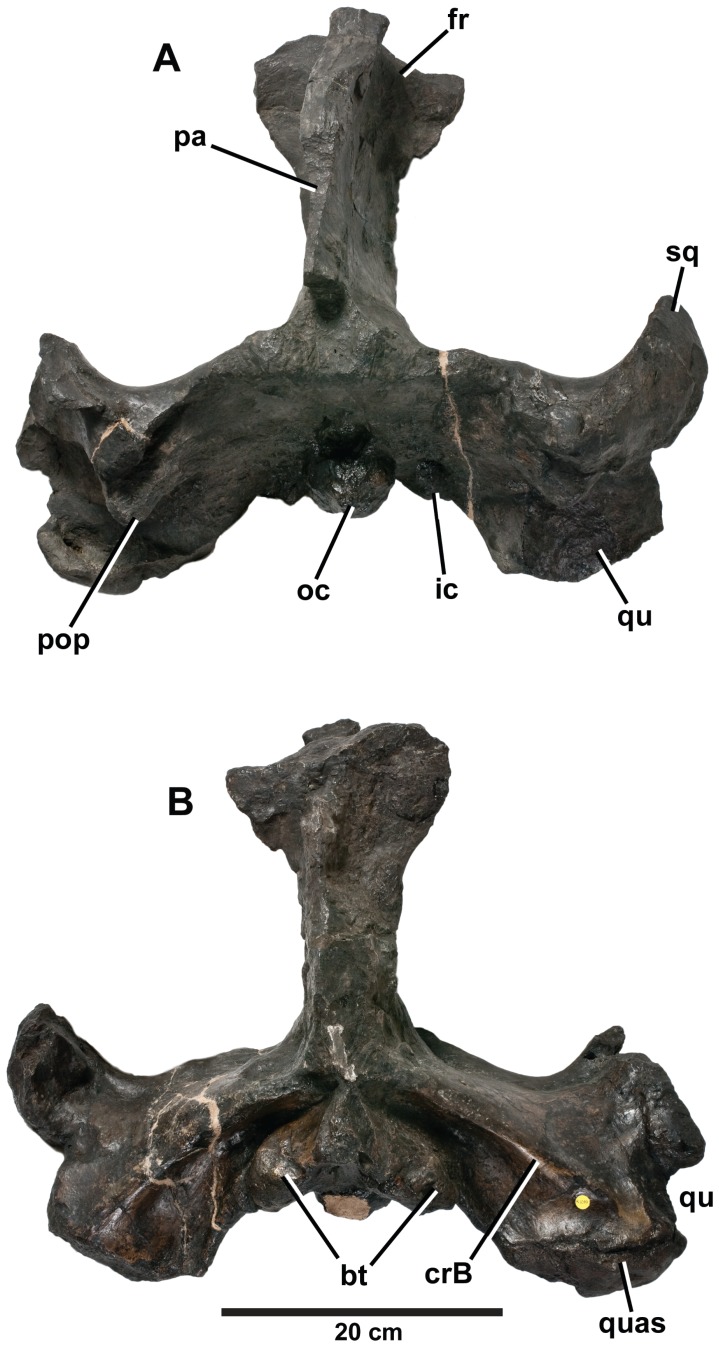
*Plesiosuchus manselii*, referred specimen NHMUK PV R1089. Braincase and intertemporal bar in: (A) dorsal view and (B) ventral view. Abbreviations: bt, basal tubera; crB, crest B; fr, frontal; ic, foramen for the internal carotid artery; oc, occipital condyle; pa, parietal; pop, paroccipital process; qu, quadrate; quas, quadrate articular surface; sq, squamosal.

**Figure 15:**
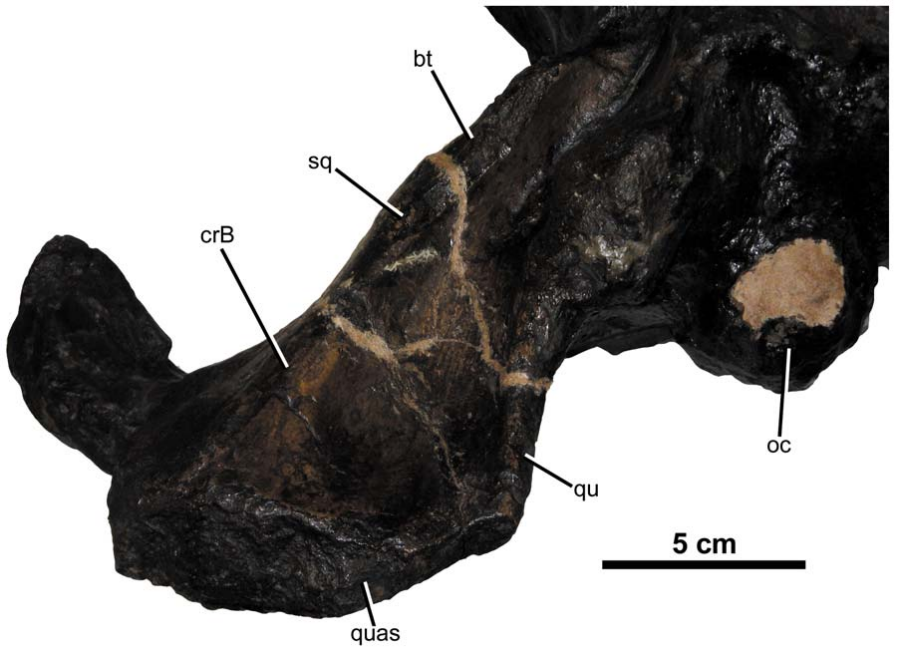
*Plesiosuchus manselii*, referred specimen NHMUK PV R1089. Close-up on the articular surface of the right quadrate. Abbreviations: bt, basal tubera; crB, crest B; oc, occipital condyle; qu, quadrate; quas, quadrate articular surface; sq, squamosal.

**Figure 16:**
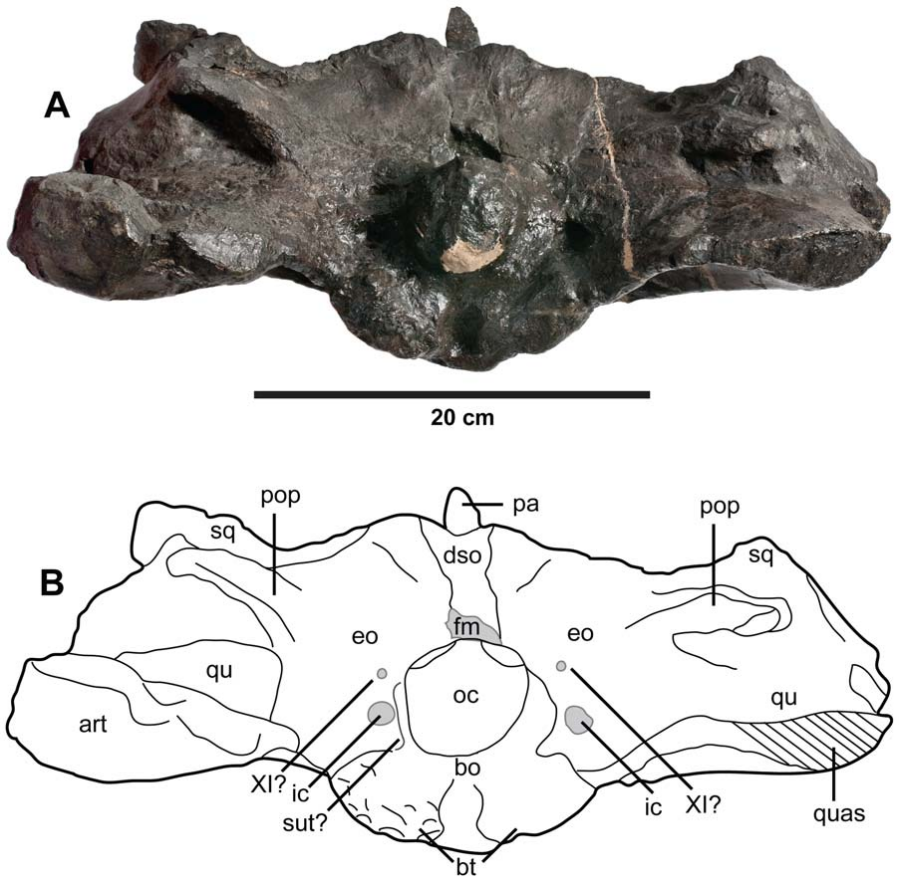
*Plesiosuchus manselii*, referred specimen NHMUK PV R1089. Braincase in occipital view, (A) photograph and (B) line drawing (filled grey areas represent foramina). Abbreviations: XI?, foramen for cranial nerve XI?; art, articular; bo, basioccipital; bt, basal tubera; dso, depression for the supraoccipital; eo, exoccipital; fm, foramen magnum; ic, foramen for the internal carotid artery; oc, occipital condyle; pa, parietal; pop, paroccipital process; qu, quadrate; quas, quadrate articular surface; sq, squamosal; sut?, suture?.

**Figure 17:**
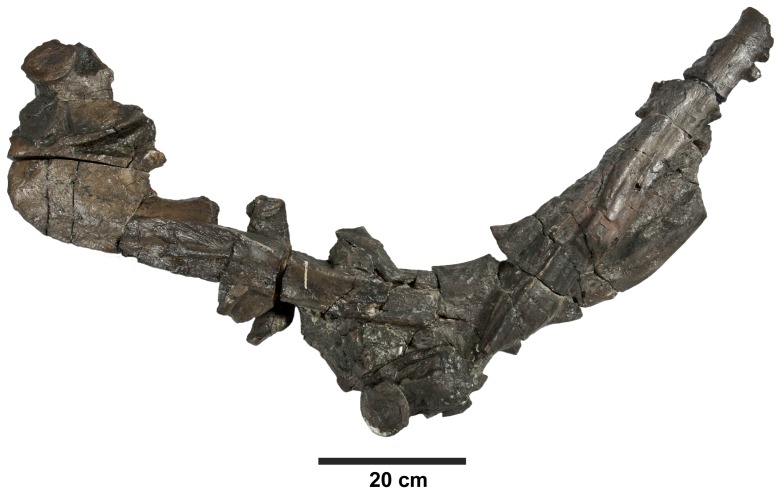
*Plesiosuchus manselii*, holotype NHMUK PV OR40103a. Mandible, with the left ramus is lateral view and the right ramus in medial view.

**Figure 18:**
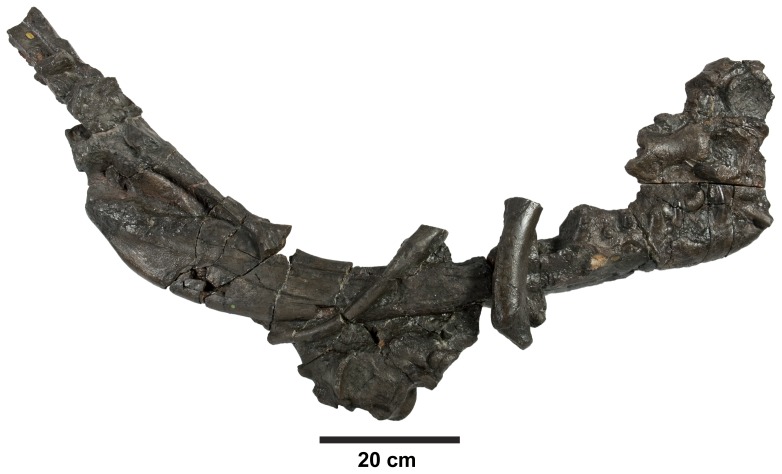
*Plesiosuchus manselii*, holotype NHMUK PV OR40103a. Mandible, right ramus in lateral view.

**Figure 19:**
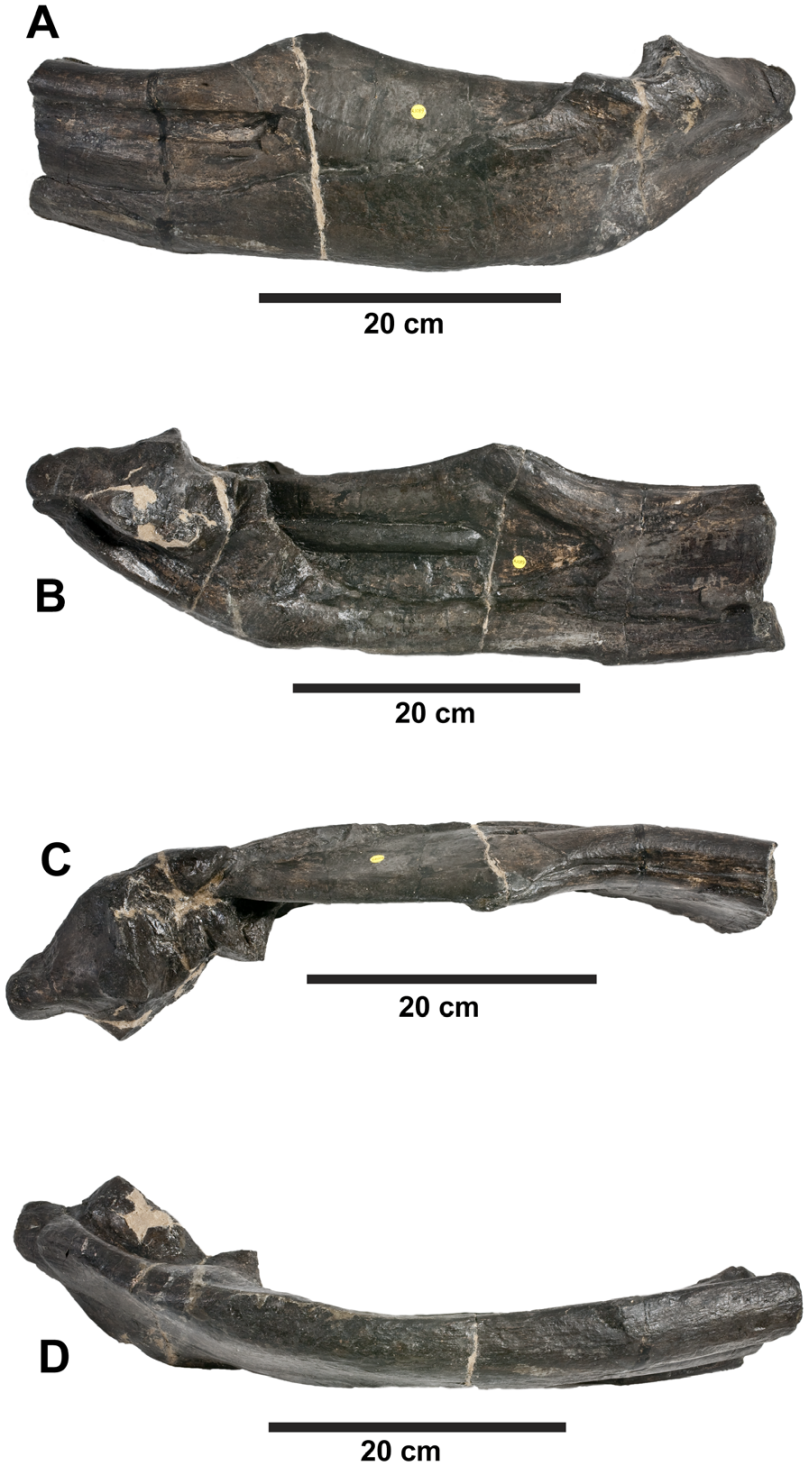
*Plesiosuchus manselii*, referred specimen NHMUK PV R1089. Posterior half of the left mandibular ramus in: (A) lateral view, (B) medial view, (C) dorsal view and (D) ventral view.

**Figure 20:**
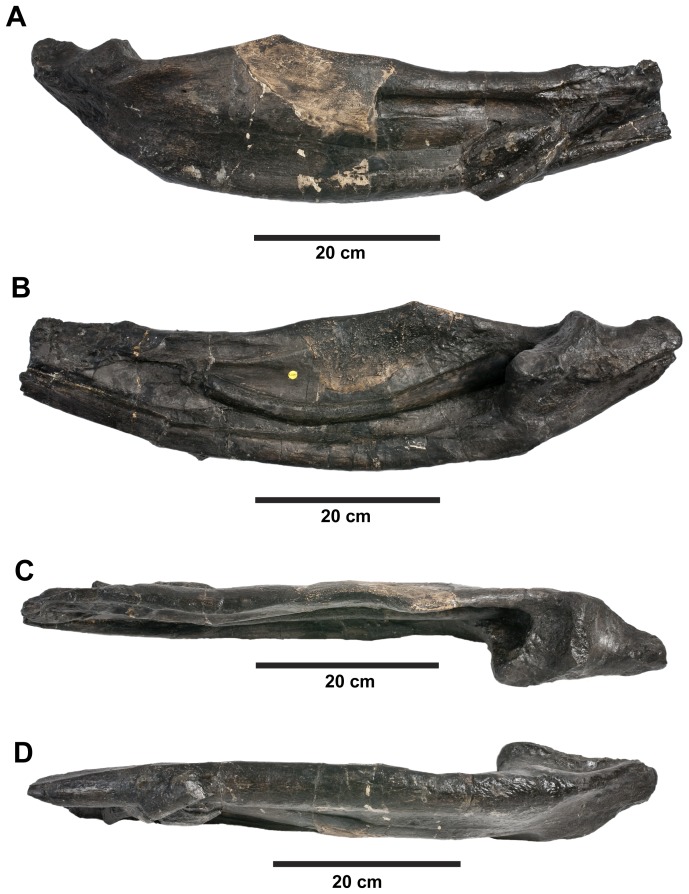
*Plesiosuchus manselii*, referred specimen NHMUK PV R1089. Posterior half of the right mandibular ramus in: (A) lateral view, (B) medial view, (C) dorsal view and (D) ventral view.

**Figure 21:**
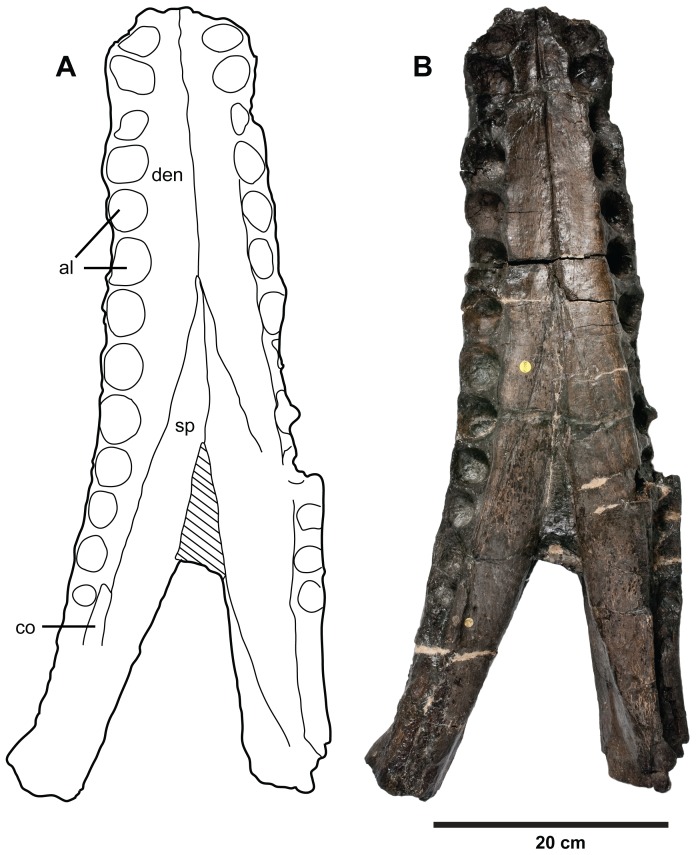
*Plesiosuchus manselii*, referred specimen NHMUK PV R1089. Mandibular symphysis in dorsal view, (A) line drawing and (B) photograph. Abbreviations: al, alveolus; co, coronoid, den, dentary; sp, splenial.

**Figure 22:**
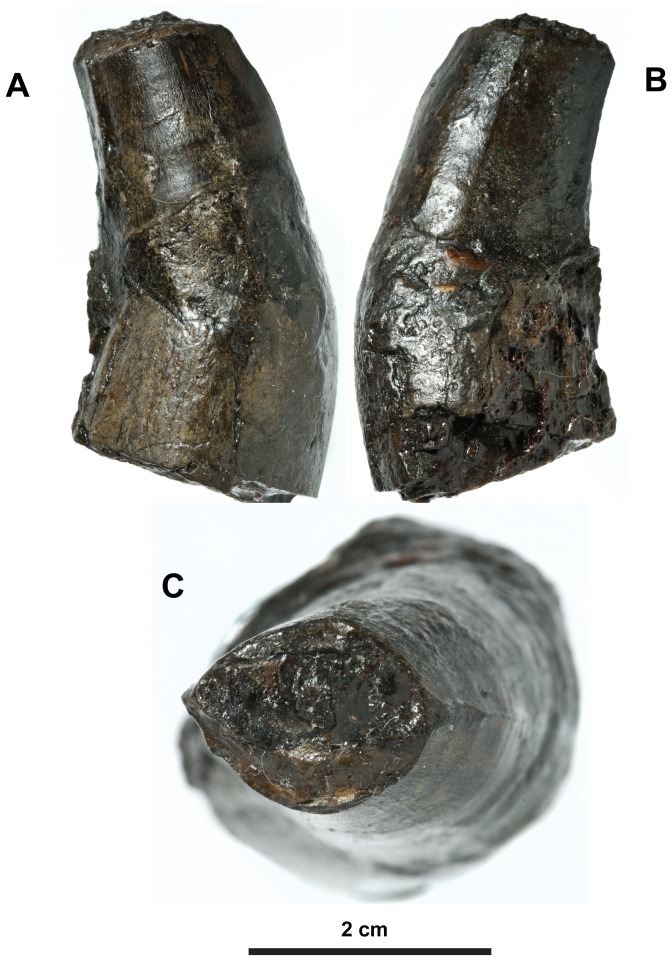
*Plesiosuchus manselii*, holotype NHMUK PV OR40103a. Isolated tooth crown in: (A) right lateral view, (B) left lateral view and (C) dorsal (apical) view.

**Figure 23:**
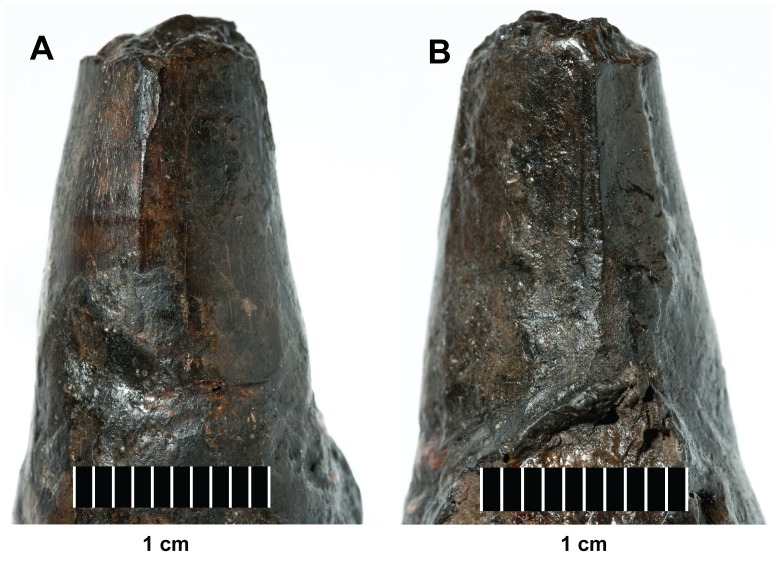
*Plesiosuchus manselii*, holotype NHMUK PV OR40103a. Close-up on the carinae of an isolated tooth crown, (A) anterior carina and (B) posterior carina.

The genus *Dakosaurus* has been known for over 150 years, and it was among the handful of large marine reptiles discovered in early–mid 19^th^ century Europe that helped reveal a hitherto unknown ancient fauna of peculiar, predatory reptiles from the Mesozoic. Since that time numerous new species have been placed in the genus. The recent phylogenetic analysis of Young & Andrade [10] and the taxonomic changes necessitated by that analysis indicated that the genus *Dakosaurus* had four valid species [22]–[25]: *D. andiniensis* (Fig. 1), *D. maximus* [23] (Figs. 2, 3, 4, 5, 6, 7, 8), *D. manselii* [24] (Figs. 9, 10, 11, 12, 13, 14, 15, 16, 17, 18, 19, 20, 21, 22, 23) and *D. nicaeensis* [25]. Furthermore, two species originally assigned to *Dakosaurus* were referred by Young & Andrade [10] to the genus *Geosaurus*: *D. lapparenti* and *D. carpenteri*. However, the latter was recently given its own genus, *Torvoneustes* [11]. Most of the Callovian–Oxfordian teeth from England, France and Poland that previously were referred to *Dakosaurus* are now considered as belonging to a new genus (still in press [2]), whereas another intriguing specimen, NHMUK PV R486, is considered Geosaurinae indeterminate [2]. Furthermore, incomplete material from the Kimmeridgian of Mexico may represent a fifth species of *Dakosaurus* [26], [27] but this is currently unclear (see discussion below). As is clear, *Dakosaurus* had a wide geographic range, with specimens known from Argentina, England, France, Germany and Switzerland [1], and possibly also Spain [28]. It may have had a worldwide distribution during the Mesozoic.

Recently, however, it has been suggested that *Dakosaurus manselii* may also not belong within the genus *Dakosaurus*. This contention was first suggested by Young *et al*. [14], based on a subsidiary phylogenetic analysis (presented in their supplementary material and differing from their primary analysis in the use of some ordered characters) that found *D. manselii* to be the sister taxon of the clade *Geosaurus* + *Dakosaurus*. This change in position was solely based on dental characters. Young *et al*. [2] re-iterated in their discussion of metriorhynchid denticle evolution that the taxonomic affinities of *D. manselii* are currently unclear. They noted that *D. manselii* has microscopic denticles (whereas both *D. maximus* and *D. andiniensis* have macroscopic denticles), and apicobasally aligned ridges on both the labial and lingual surfaces (which *D. maximus* and *D. andiniensis* lack). Updated anatomical information, therefore, is necessary for resolving the affinities of *D*. *manselii*. Furthermore, the systematic placement of *D*. *manselii* also has bearing on the systematics, especially the generic placement, of *D*. *nicaeensis*, a poorly-understood species that shares an unusual large dentition with both *D*. *manselii* and *D*. *maximus*. If *D*. *manselii* does not belong to the same subclade as *D*. *maximus*, then this distinctive dentition would be homoplastic and insufficient for assigning *D*. *nicaeensis* to *Dakosaurus*. Resolving the phylogenetic position of *D*. *manselii*, therefore, is currently one of the most pressing issues in metriorhynchid systematics and a keystone upon which rests many wider issues of metriorhynchid classification and phylogeny.

Despite the recent upsurge of interest in metriorhynchid phylogeny and evolution, the original specimens of *Dakosaurus maximus* and “*Dakosaurus*” *manselii* from Europe have received little attention. In fact, they have been only briefly described in the literature (with Fraas’ [4] monograph of *Geosaurus* [*Cricosaurus*] *suevicus* and *Dakosaurus maximus* being the only exception), which makes it difficult to incorporate them into phylogenetic analyses and compare them with newly discovered specimens. This is especially surprising given that several new species and specimens have been assigned to the genus *Dakosaurus* in recent years, including spectacularly preserved material that has revealed the strong bite forces and theropod-like skulls characteristic of the genus [19], [20] (Fig. 1). Here we redescribe the type specimens of *Dakosaurus maximus* and “*D.*” *manselii*, as well as a large partial skull and mandible assigned to “*Pliosaurus trochanterius*” (and later *Machimosaurus mosae*), which we conclusively demonstrate is a metriorhynchid and, for the first time, refer to “*D.*” *manselii*. These redescriptions reveal a number of characters unique to each species, and allow us to present a comprehensive osteology for these important historical taxa. Furthermore, these redescriptions highlight significant craniodental differences between *D. maximus* and “*D.*” *manselii*, and show that there are numerous autapomorphies shared by *D. maximus* and *D. andiniensis* that are absent in “*D.*” *manselii*. We then incorporate new information gleaned from these redescriptions into a revised phylogenetic analysis of Thalattosuchia. Most importantly, this analysis does not recover a distinct grouping of *D*. *maximus*, *D*. *andiniensis*, and “*D*.” *manselii*, which supports the removal of the latter species into its own genus, the resurrected *Plesiosuchus*. Based on our greater understanding of Geosaurini craniodental form we also revise the generic diagnoses for *Geosaurus* and *Torvoneustes*. Finally, based on our craniodental descriptions of *D. maximus* and “*D.*” *manselii* we outline hypothetical feeding behaviours for both species, and hypothesise that trophic specialisation enabled these two species to co-exist in the same ecosystem.

### Institutional Abbreviations

BRSMG, Bristol City Museum and Art Gallery, Bristol, UK; BSPG, Bayerische Staatssammlung für Paläontologie und Geologie, München, Germany; CAMSM, Sedgwick Museum, Cambridge, UK; CM, Carnegie Museum of Natural History, Pittsburgh, PA, USA; GLAHM, Hunterian Museum, Glasgow, UK; JME, Jura Museum, Eichstätt, Germany; MOZ, Museo Profesor J. Olsacher, Zapala, Argentina; MUJA, Museo del Jurásico de Asturias, Colunga, Spain; NHMUK, Natural History Museum, London, UK; NMS, Naturmuseum Solothurn, Switzerland; OUMNH, Oxford University Museum of Natural History, Oxford, UK; SMNS, Staatliches Museum für Naturkunde Stuttgart, Germany.

### Historical Overview of *Dakosaurus maximus*

The first species assigned to the genus *Dakosaurus* was *D*. *maximus*, which was first erected as *Geosaurus maximus* by Plieninger [23], for an isolated tooth found at Schnaitheim, near Heidenheim, Baden-Württemberg, Germany (upper Kimmeridgian; *Hybonoticeras beckeri* Sub-Mediterranean ammonite Zone). Plieninger [29] later referred a partial dentary with six *in situ* teeth preserved in lithographic limestone from Ulm to *Geosaurus maximus*. Quenstedt [30], [31] initially considered isolated teeth from Schnaitheim, very similar to those of figured by Plieninger, as belonging to the theropod dinosaur genus *Megalosaurus*. However, later he erected the name *Dakosaurus* for the Schnaitheim teeth and Plieninger’s species [32], [33]. Furthermore, Quenstedt [33] referred a dentigerous bone (probably a partial maxilla) from Schnaitheim with three *in situ* crowns to *D. maximus*. As the type material of *D*. *maximus* is missing, Young & Andrade [10] suggested a skull and mandible described by Fraas [4] should be the neotype of the species. This specimen (SMNS 8203; Figs. 2, 3, 4) was found at Staufen, Baden-Württemberg, Germany, and was also from the upper Kimmeridgian *H*. *beckeri* Sub-Mediterranean ammonite Zone. Fraas [4] described a second *D. maximus* skull (SMNS 10819a, b; Fig. 5) from the upper Kimmeridgian of Sontheim an der Brenz, Baden-Württemberg, Germany.

During the 19^th^ century there were numerous species assigned to *Dakosaurus*, most of which were erected for isolated teeth. Fraas [4] synonymised all *Dakosaurus* species with the exceptions of *D. manselii* (he considered *D*. *manselii* to be either a junior synonym of, or closely related to, *D. maximus*) and the tooth taxon *D. paradoxus* with *D. maximus*. Central to this argument, Fraas [4] demonstrated that the various morphological differences used to erect these numerous tooth taxa were actually part of a continuum of variation that was normal for a single species. This argument followed an earlier, but long neglected, study by Mason [34], who discussed the variation in mediolateral compression and symmetry in *Dakosaurus* teeth as being related to position in the tooth row, and to which bone the teeth belonged.

Two historic tooth taxa now considered as synonymous with *D*. *maximus*, *Liodon paradoxus* [35] and *Teleosaurus suprajurensis* [36], were erected for isolated teeth discovered in the lower Tithonian *Diceras* Limestones near Kelheim, Bavaria, Germany. *Teleosaurus suprajurensis* was considered to be a subjective junior synonym of *D. maximus* by Lydekker [3], von Zittel [37] and Fraas [4]. *Liodon paradoxus* was referred to the early Tithonian species *Cricosaurus grandis* [38] by von Zittel [37], while Fraas [4] referred the species to *Dakosaurus*, based on a mandible with *in situ* teeth and isolated teeth from Schnaitheim. All teeth referred to *D. paradoxus* are less robust than those that had been referred to *D. maximus* during the mid 1800s, with a narrower labiolingual cross-section (see Fraas [4]: Plate 2, Figs 1, 12, 13, and 14). This is the primary reason why Fraas [4] retained *D. paradoxus* as a separate species. Interestingly, however, the mandible Fraas [4] referred to *D. paradoxus* only preserves the anterior-most dentition. One specimen of *Dakosaurus*, SMNS 10819a, preserves the premaxilla and maxilla with *in situ* crowns (Fig. 5A). It demonstrates that the premaxillary and newly erupted maxillary crowns of *D. maximus* are notably less robust (narrower labiolingual cross-section) than fully-erupted maxillary crowns (confirming Mason [34]). In other words, *Dakosaurus* exhibits heterodonty across the tooth row, and not all of its teeth possess the ‘characteristic’ robust morphology that was assumed by early workers. As such, there are no grounds to retain *D. paradoxus* as a separate species.

Another historic tooth taxon now considered as *D*. *maximus*, *Dakosaurus gracilis* is known from small isolated teeth discovered in lower Tithonian deposits near Steinheim, Baden-Württemberg, Germany [39]. Fraas [4] did not consider *D. gracilis* as a separate species, but regarded it to be at most a variety of *D. maximus*. An incomplete skeleton discovered from an unnamed Lower Cretaceous (early Hauterivian in age) formation in Département du Var, Provence-Alpes-Côte d’Azur, France was referred to as *Dacosaurus maximus* var. *gracilis* (sic) [40]. Subsequently [41], another incomplete skeleton from a nearby locality (late Valanginian in age) was named *Dacosaurus lapparenti* (sic), and is now known as *Geosaurus lapparenti* [10].

Yet another historic taxon, *Dakosaurus lissocephalus*, is known from a poorly preserved and dorsoventrally crushed skull (CAMSM J29419) discovered in the upper Kimmeridgian (*Aulacostephanus eudoxus* Sub-Boreal ammonite Zone) Lower Kimmeridge Clay Formation of Ely, Cambridgeshire, England [42]. Lydekker [3] considered this species to be a subjective junior synonym of *D. maximus*, while Fraas [4] provisionally synonymised the two. Young & Andrade ([10]: appendix) considered *D. lissocephalus* and *“Dakosaurus” manselii* not to be conspecific, due to the shape of the supratemporal fenestra, squamosal, and parietal in *D. lissocephalus* being more reminiscent of *D. andiniensis* and *D. maximus*. Furthermore, they considered the synonymy between *D. maximus* and *D. lissocephalus* provisional. Here, we formally synonymise *D*. *lissocephalus* and *D*. *maximus*. This is due to two similarities that they share, unique to other *Dakosaurus* species. First, *D. lissocephalus* like other metriorhynchids has a quadrate distal articular surface separated into two protuberances (condyles) by a sulcus [5]. *Plesiosuchus manselii*, on the other hand, lacks this sulcus (Fig. 15). Secondly, isolated teeth from the *D. lissocephalus* type locality (NHMUK PV OR20283) share the same suite of characters as the German *D. maximus* teeth (no apicobasally aligned ridges, large conspicuous denticles; Fig. 8). The *P. manselii* teeth, however, possess apicobasal ridges (of low-relief) and small denticles (Figs. 22, 23). As the type locality (a quarry in Ely) has subsequently been flooded, discovering more material to confirm these observations is difficult.

Finally, another historical taxon, *Leiodon primaevum* is known from isolated teeth discovered in the upper Kimmeridgian (*A*. *autissiodorensis* Sub-Boreal ammonite Zone) Argiles de Châtillon Formation of Boulogne-sur-Mer, Pas-de-Calais, France [43]. Sauvage [44] later placed *L. primaevum* in *Dacosaurus* (sic) as *D. primaevus*. Lydekker [3], von Zittel [37] and Fraas [4] all considered this species to be a subjective junior synonym of *D. maximus*. We agree, as isolated teeth from the type locality (NHMUK PV OR32414; SMNS 57210) share the same suite of characters as the German *D. maximus* and the English teeth from Ely (see above). A partial left maxilla (SMNS 56999) from the locality also shares the same distinctive maxillary ornamentation as *D. maximus* (see description below; Fig. 7).

### Historical Overview of *Plesiosuchus manselii*

The holotype of *Plesiosuchus manselii* is a broken and incomplete skull (NHMUK PV OR40103; Figs. 9, 10, 11, 12, 13) with a mandible and isolated post-cranial remains (NHMUK PV OR40103a; Figs. 17, 18, 22, 23) from a large individual discovered in the upper Kimmeridgian (*A. autissiodorensis* Sub-Boreal ammonite Zone) Lower Kimmeridge Clay Formation of Kimmeridge Bay, Dorset, England. The specific epithet *manselii* is frequently misspelt in the literature, generally as manseli or mansellii [3], [45]–[47]. The type and referred specimens were given to the British Museum (now in the Natural History Museum, London) by John Clavell Mansel-Pleydell in 1866. During the 1860s Mansel-Pleydell discovered the remains of several large-bodied marine reptiles along the coast of Dorset, most especially at Kimmeridge Bay. He discovered the remains of *P. manselii* in a reef at Kimmeridge Bay, exposed at low tide [48].

The holotype of *Plesiosuchus manselii* was described by Hulke in two papers. In the first, Hulke [48] described the right mandibular ramus, isolated vertebrae, an isolated premaxilla, a femur and a dentigerous bone that he tentatively referred to as the “upper maxilla” (these specimens are now curated as NHMUK PV OR40103a, although the isolated premaxilla is now part of NHMUK PV OR40103). He referred the specimens to Geoffroy’s [49] *Steneosaurus rostro-minor*. Hulke [48] posited that NHMUK PV OR40103a was probably identical to Cuvier’s [50] second Honfleur gavial “tête à museau plus court”, that the dentition of NHMUK PV OR40103a was identical to *Dakosaurus maximus*, and that all these species/specimens could be referred to *Steneosaurus rostro-minor*. It must be noted that metriorhynchids were poorly known at this time, with *Dakosaurus maximus* known only from isolated teeth and a fragments of dentigerous bones with *in situ* crowns, while Cuvier’s [50] “tête à museau plus court” was a chimera of two metriorhynchid species (*Metriorhynchus superciliosus* and *M. geoffroyii* [1], [51]). The characteristics Hulke [48] used to unite these specimens are now known to be either metriorhynchid apomorphies (e.g. oval, “spoon-shaped” external nares; three premaxillary alveoli; absence of external mandibular fenestrae; distinct coronoid process on the mandible) or Geosaurini apomorphies (e.g. bicarinate serrated dentition). Other characteristics used were subsequently found to have been widespread in Mesozoic crocodylomorphs (e.g. teeth with apicobasal ‘striations’; teeth that are unequally convex, that have some degree of mediolateral compression and recurve lingually; amphicoelous vertebrae). A note was added to the end of the first publication stating a “considerable part of the skull” had been discovered through further examination of the material presented to the British Museum.

In the second paper, Hulke [24] described a skull, which is preserved in two sections: the rostrum and the occiput (the latter with partial supratemporal arches preserved; now curated as NHMUK PV OR40103; Fig. 9). Initially, the then matrix-encased skull was believed to pliosaurian and set aside; it was the preparator Mr Davies that realised the skull material was in fact crocodylian in nature [24]. It was here that the specific epithet *manselii* was erected as *Steneosaurus Manselii* (sic). The “head” (NHMUK PV OR40103) and the “lower jaw and associated post-crania” (NHMUK PV OR40103a, the specimen described by Hulke [48]) have been considered to be from the same individual. Hulke ([24]:167) stated: “The general agreement of their dimensions, and their discovery near together (in a reef exposed at low water in Kimmeridge Bay), make it highly probable that this head and the lower jaw both belonged to one individual”. We agree that the two specimens are most likely from the same individual, especially as the size of the two specimens is comparable.

Interestingly, the isolated bone fragment referred to as the “upper maxilla” has never been figured and cannot be located. Hulke [48] describes the specimen as being a fragment of 14 cm in length, containing five alveoli of which four still have portions of teeth remaining *in situ*. Furthermore, the bone is not mentioned in the latter publication (unlike the mandible and premaxilla). There is a possibility as to why this specimen cannot be located. A note in the NHMUK specimen register beside NHMUK PV OR40103 states that some of the material was destroyed, with a date of 1 August 1931. Unfortunately, what was destroyed is not stated. As both NHMUK PV OR40103 and NHMUK PV OR40103a suffer pyrite decay and require periodic conservation, it is possible that the “upper maxilla” was destroyed after extensive decay. A box of NHMUK PV OR40103 fragments was discovered by one of us (LS), and it could represent some of the destroyed material.

Owen [52] erected the genus *Plesiosuchus* for *Steneosaurus manselii* as he considered it to be more similar, in a morphological sense, to extant crocodylians than to *Steneosaurus*. Woodward [53] referred the species to the genus *Dakosaurus*. Lydekker ([3]:92) saw no reason to separate *P. manselii* from *D. maximus*, considering the former to be a subjective junior synonym of the latter. It appears as if this decision was based on dental characteristics, as previous authors noticed the similarity between the dentition of *P. manselii* and *D. maximus* [24], [48], [52], [53]. Woodward’s [53] taxonomic decision could not have been based on craniomandibular morphology, as the first *D. maximus* skull was not described until several years later [4]. Fraas [4] regarded *P. manselii* either as a junior synonym of, or closely related to, *D. maximus*; interestingly, however, he did not include *P. manselii* in his synonymy list of *D. maximus*. The phylogenetic analysis of Young & Andrade [10] supports the hypothesis that the two are separate species. As stated above, there has been a growing realisation that *Dakosaurus*/*Plesiosuchus manselii* may not belong within *Dakosaurus* and that its taxonomic affinities are unclear [2], [14].

### The Lost ‘*Pliosaurus trochanterius*’ Skull and Mandible

In 1866, Mansel-Pleydell presented numerous marine reptile fossils he discovered at Kimmeridge Bay to the British Museum. One of these specimens, a mandible and an incomplete skull (braincase with part of the supratemporal arches), was from an individual even larger than the “*D*.” *manselii* holotype (NHMUK PV OR40103). As with NHMUK PV OR40103, this specimen (NHMUK PV R1089; Figs. 14, 15, 16, 19, 20, 21) was initially considered to belong to a pliosaurid plesiosaur. It was originally described by Owen [54] within his species *Pliosaurus trochanterius*. However, Owen never provided evidence to show that NHMUK PV R1089 belonged within the species. There are no overlapping elements, as Owen [55], [56] erected *Pliosaurus trochanterius* based on an isolated femur (according to Brown [57] it is actually a humerus). Additionally, they come from different localities: *Pliosaurus trochanterius* is from the early Tithonian of Shotover Hill, Oxfordshire, England, whereas NHMUK PV R1089 is from the early Tithonian of Kimmeridge Bay, Dorset. Eudes-Deslongchamps ([58]:329) quickly demonstrated the crocodylian affinities of NHMUK PV R1089, and referred it to the metriorhynchid genus *Metriorhynchus*, as did Woodward ([53]:502). Lydekker ([3]:104), however, believed the specimen belonged to the teleosaurid species *Machimosaurus mosae*, although he noted that it lacked the anterior transverse expansion of the mandibular symphysis seen in teleosaurids. Tarlo [59] considered the specimen to be crocodylian, while Buffetaut [60] considered it to be a large metriorhynchid, probably *Dakosaurus*. More recently, Benton & Spencer [61] figured NHMUK PV R1089 as the plesiosaur *Colymbosaurus trochanterius*, while Vignaud [51] referred the specimen to the teleosaurid species *Machimosaurus mosae*.

Following Hua *et al*. [62], there are two valid species of *Machimosaurus* in the Late Jurassic of Europe: the type species *Machimosaurus hugii* (early–late Kimmeridgian of France, Portugal and Switzerland) and *M. mosae* (latest Kimmeridgian of France). Comparing NHMUK PV R1089 to the mandibles of *M. hugii* [63] and *M. mosae* [62] clearly shows it does not belong to *Machimosaurus*. Both species of *Machimosaurus* possess external mandibular fenestrae and an anterior transverse expansion of the mandibular symphysis, whereas NHMUK PV R1089 lacks both features. Additionally, both species of *Machimosaurus* lack the prearticular, which is present in NHMUK PV R1089. The absence of the external mandibular fenestrae is a metriorhynchid apomorphy, while the anterior transverse expansion of the mandibular symphysis and loss of the prearticular are teleosaurid apomorphies [5], [10] (Hua pers. comm. 2011). In addition, NHMUK PV R1089 has far fewer dentary alveoli than either *Machimosaurus* species: 13 compared to their 19–25. This extreme reduction in dentition is observed in geosaurine metriorhynchids (Table 1).

**Table 1:** List of characters to differentiate between the various Geosaurini genera present in the Late Jurassic of Europe.

		*Torvoneustes*: BRSMG Cd7203, BRSMG Ce17365		*Geosaurus*: BSPG AS-VI-1, NHMUK PV OR37020, NHMUK PV R1229, NHMUK PV R1230		*Plesiosuchus*: NHMUK PV OR40103, NHMUK PV OR40103a, NHMUK PV R1089		*Dakosaurus*: CAMSM J29419, NHMUK PV OR20283, SMNS 8203, SMNS 82043, SMNS 10819
**Dentition**								
Premaxillary alveoli count			3		3		3	3
Maxillary alveoli count			14 a		14		14	13
Dentary alveoli count			?		13 a		13	12
Dentary alveoli adjacent to symphysis			?		8 a		9	4
Enamel ornamentation			Intense ornamentation on both labial and lingual surfaces. Basal half has apicobasally aligned ridges, apical half has pronounced anastomosed pattern		No conspicuous enamel ornamentation		Ornamentation largely inconspicuous, apicobasal ridges on labial and lingual surfaces but of low relief	No conspicuous enamel ornamentation
Facetting on labial surface			No		Yes, three apicobasal facets		No	No
Crown shape			Conical		Laminar/strongly compressed		Robust, some compression	Robust, some compression
Wear facets on mesial and distal margins			No		No		No	Yes
Denticles			Microscopic, but in apical half the denticles are hard to distinguish from enamel ornamentation		Microscopic		Microscopic	Macroscopic
**Cranium**								
Premaxillary lateral plates			No		No		No	Yes
Distance between premaxilla and nasal			∼ Equal to the midline length of the premaxilla		Less than half the midline length of the premaxilla		∼ Equal to the midline length of the premaxilla	Less than half the midline length of the premaxilla
Rostrum-to-skull roof dorsal margin			Concave		Concave		Concave	Convex
Prefrontals in dorsal view: angle between the inflexion point on the lateral margin and the anteroposterior axis of the skull			∼70 degrees		∼70 degrees		∼70 degrees	∼50 degrees
Intratemporal flange anterior development			Does not reach minimum interorbital distance		Does not reach minimum interorbital distance		?	Reaches minimum interorbital distance
Palatine anterior development along the midline			Maxillopalatine suture reaches as far anteriorly as the 9th maxillary alveoli		Comparison between the specimens suggests palatine reached level to either the 8th or 9th maxillary alveoli		Level to 4th maxillary alveoli	Palatine not preserved, maxillae form the secondary palate up to at least the 8th maxillary alveoli
**Mandible**								
Dentary lateral and medial plates	?				No		No	Two ridges either side of the dentary alveoli (prominent between D6 to D12)
Mandibular lateral groove ( = surangulodentary groove)	No large foramen at dentary terminus				No large foramen at dentary terminus		No large foramen at dentary terminus	Large foramen present at dentary terminus
Reception pits along the dentigerous bones	No				Yes, on the lateral margin of the dentary and medial surface of maxilla		No	Yes, between the alveoli of the upper and lower jaw dentigerous bones
**Postcrania**								
Humerus deltopectoral crest shape	Prominent convex curve that is continuous with the proximal articular surface				?		Prominent and subtriangular, easily distinguished from proximal articular surface	Prominent and subtriangular, easily distinguished from proximal articular surface
Femur posteromedial tuber enlargement	Does not encompass the entire medial femoral surface in dorsal view				Does not encompass the entire medial femoral surface in dorsal view		Encompasses the entire medial femoral surface in dorsal view, creating a pronounced medial deflection of femoral head	?
Ischium blade	Posterior-edge is a convex curve				Posterior-edge is a convex curve		?	Posterior-edge is subrectangular

The hunmerus of Geosaurus in the Kimmeridgian-Tithonian is unknown, but the Valanginian-Hauterivian species *Geosaurus lapparenti* has a peculiar humerus morphology that does not conform with either the *Torvoneustes*, *Plesiosuchus* or *Dakosaurus* humeral shapes [40], [41].

a Estimate.

Furthermore, the dentary interalveolar spaces of NHMUK PV R1089 are very small, far smaller than in both *Machimosaurus* species, and most thalattosuchians. In teleosaurids [5], [62]–[64], metriorhynchine metriorhynchids [5], [64], [65] and basal geosaurine metriorhynchids (“*Metriorhynchus*” *brachyrhynchus* and “Mr Leeds’ specimen” [2], [5]) the dentary symphyseal interalveolar spaces are variable in size, ranging from being larger than the proceeding and preceding alveolus, to being half the size. The extreme reduction in dentary symphyseal interalveolar distances in NHMUK PV R1089 (always being less than a quarter the length of the immediate alveoli, and usually even smaller; Fig. 21) is characteristic of Geosaurini metriorhynchids (*Dakosaurus maximus*: Figs. 2, 3; *Torvoneustes carpenteri* [18]). This reduction in symphyseal interalveolar spaces means the typical thalattosuchian ‘diastema’ between dentary alveoli 4 and 5 is absent [2], [5], [58], [62], [64], [65]. Curiously, both the extreme reduction in symphyseal interalveolar spaces and the absence of the D4–D5 ‘diastema’ are observed in the holotype of the geosaurine metriorhynchid *Suchodus durobrivensis* (NHMUK PV R1994: a mandibular symphysis). These unusual two characteristics were first highlighted by Lydekker ([66]:287); moreover, he noted there was “marked resemblance between” the *Suchodus durobrivensis* holotype and NHMUK PV R1089. This has led two of us (MTY and LS) to begin re-examining the NHMUK Callovian metriorhynchids to determine whether the synonymy of *Suchodus durobrivensis* and “*Metriorhynchus*” *brachyrhynchus* is valid [2], [14]; as such, herein we do not follow Young *et al.* [1] in referring “*Metriorhynchus*” *brachyrhynchus* to the genus *Suchodus*.

The occiput/braincase of NHMUK PV R1089 exhibits two metriorhynchid autapomorphies: 1) enlarged carotid artery foramina ventrolateral to the foramen magnum (apomorphy was confirmed through computed tomography scanning of an Oxfordian braincase [67]); and 2) the trigeminal fossa is developed mainly posterior to the trigeminal foramen [67]. As such, we can conclusively refer NHMUK PV R1089 to Metriorhynchidae, and by extension remove it from *Machimosaurus mosae*.

Although we can refer NHMUK PV R1089 to Metriorhynchidae, can we refer it to *Dakosaurus*/*Plesiosuchus manselii*? The surangulodentary groove in NHMUK PV R1089 and NHMUK PV OR40103a is deeply excavated and strongly developed on both elements (Figs. 17, 18, 19, 20). This morphology is only observed in *D. maximus* (SMNS 8203, SMNS 82043; Figs. 2, 6) and *D. andiniensis* [19], [20] among metriorhynchids (and to some extent in the more poorly preserved specimens of *Geosaurus giganteus* [10]). This therefore allows us to assign it to Geosaurini. We can exclude both the holotype of *D*./*P. manselii* and NHMUK PV R1089 from *D. maximus* because they lack the sharp dorsal inclination of the ventral margin of dentary and the raised alveolar margins of the posterior dentary alveoli that are characteristic of this species (see below; compare Fig. 2 with Figs. 17, 18, 21). Furthermore, both the holotype of *D*./*P*. *manselii* and NHMUK PV R1089 share a cranial apomorphy: distal articular surface of the quadrate is not divided into two condyles by a sulcus (Fig. 15), which is an unusual feature among archosaurs. This suite of shared derived characters (see Table 2) allows us to refer NHMUK PV R1089 to *Dakosaurus*/*Plesiosuchus manselii*.

**Table 2:** Table of diagnostic characters for Metriorhynchidae, and various subclades, for the three Kimmeridge Bay NHMUK PV specimens.

Clades	Diagnostic characters	OR40103	OR40103a	R1089
Metriorhynchidae	Three teeth per premaxilla	Yes	?	?
	Enlarged carotid artery foramina	Yes	?	Yes
	Trigeminal fossa developed mainly posterior to the trigeminal foramen	?	?	Yes
	No external mandibular fenestrae	?	Yes	Yes
	Coronoid process on mandible	?	Yes	Yes
	Humerus flattened, shaft contributes less than 40% total humeral length	?	Yes	?
“Mr Leeds’ specimen” + Geosaurini	Ventral displacement of the dentary tooth row, such that the coronoid process is located considerably above the plane of the tooth row	?	Yes	Yes
	Coronoid process ventral to both the retroarticular process and glenoid fossa	?	Yes	Yes
	Fourteen or fewer teeth per dentary ramus	?	Yes	Yes
Geosaurini (but characters unknown in “Mr Leeds’ specimen”)	Fourteen or fewer teeth per maxilla	Yes	?	?
	Posterior expansion of supratemporal fenestrae in dorsal view (reaching at least the supraoccipital, but can even exceed the occipital condyle)	Yes	?	Yes
Geosaurini	Denticulated bicarinate dentition	Yes	Yes	?
	Dentary symphyseal interalveolar spaces are very small (less than half the size of the immediate alveoli)	?	?	Yes
	Deeply excavated surangulodentary groove	?	Yes	Yes
	Humerus is short and stocky, deltopectoral crest contacts proximal articular surface	?	Yes	?
*Plesiosuchus manselii*	Tooth enamel ornamentation: apico-basally aligned ridges of low-relief	Yes	Yes	?
	Quadrate distal articular surface not separated into two protuberances by a sulcus	Yes	?	Yes

Note that both NHMUK PV OR40103 and NHMUK PV OR40103a are the holotype of *Plesiosuchus manselii*. Note: the “Mr Leeds’ specimen” is a new genus and species; however the paper establishing these names is still in press [2].

## Methods

### Ethics Statement

We had permission to look at, and photograph, the relevant collections in the MUJA, NHMUK and SMNS. The curators whose remit includes fossil crocodylians from the MUJA (JIR-O), NHMUK (LS) and SMNS (RS) are co-authors on this manuscript. None of these specimens were purchased, donated or loaned as part of this study.

### Phylogenetic Analyses

We undertook two phylogenetic analyses to assess the evolutionary relationships of *Dakosaurus* and *Plesiosuchus* within Thalattosuchia. This analysis is the latest in a series of iterative analyses, beginning with the publication of Young & Andrade [10], in which our research group (led by MTY) has added new character data and newly-described taxa to a growing discrete character dataset. The analysis presented here is a revised version of the most recent analysis by our group, that published by Young *et al*. [2]. See the online supplementary material for sources of character coding and the character list (Text S1) and the character scores (Text S2). Here, craniomandibular and dental characters make up 73% (175/240) of the character list, while the post-cranial characters contribute 27% (65/240). The analysis presented here differs from that of Young *et al*. [2] in that:

1. 39 new or revised characters have been added.
2. We have revised the character codings of *Dakosaurus maximus* and *Plesiosuchus manselii* based on first-hand examination and the anatomical revisions presented in this monograph.
3. *Erpetosuchus granti* is no longer included in the analysis, with the outgroup taxon now being *Postosuchus kirkpatricki*. Recent comprehensive phylogenetic work on the relationships of basal archosaurs [68], [69] strongly supports the close relationship of *Postosuchus* (and related rauisuchians) with crocodylomorphs but does not corroborate previous hypotheses [70], [71] that *Erpetosuchus*, a taxon known only from highly incomplete material, is a close crocodylomorph outgroup. Additionally, the fragmentary nature of known *Erpetosuchus* specimens results in a high amount of missing data when this taxon is scored in phylogenetic analyses, which is not a desired characteristic of an outgroup taxon used to root phylogenetic trees.
4. We have expanded the non-metriorhynchid taxon selection substantially, with eight more teleosaurids and 16 non-thalattosuchians, resulting in 73 total taxa.
5. The putative *Dakosaurus* specimens from Mexico were removed due to their poor preservation and the fact that we cannot be sure they belong to the same taxon.
6. The metriorhynchid *Purranisaurus potens* was removed, as its type specimen is currently under re-description by one of us (MF) with colleagues. This redescription will result in a more confident set of character scores for this taxon.
7. Finally, we recoded “*Metriorhynchus*” *brachyrhynchus* due to the uncertainty of whether the *Suchodus durobrivensis* holotype is a junior synonym of the former (see above discussion regarding dentary interalveolar spaces). This is currently being investigated by two of us (MTY and LS).

The two phylogenetic analyses were carried out using TNT v1.1 (Willi Hennig Society Edition) [72]. They differed in that: 1) the first analysis had all characters treated as unordered, while 2) in the second analysis 40 multi-state characters were treated as ordered (transformational sequences). The 1^st^, 7^th^, 8^th^, 10^th^, 13^th^, 25^th^, 38^th^, 39^th^, 42^nd^, 43^rd^, 47^th^, 50^th^, 56^th^, 58^th^, 69^th^, 86^th^, 87^th^, 96^th^, 126^th^, 132^nd^, 133^rd^, 151^st^, 152^nd^, 154^th^, 156^th^, 166^th^, 179^th^, 181^st^, 182^nd^, 183^rd^, 184^th^, 198^th^, 202^nd^, 214^th^, 218^th^, 225^th^, 228^th^, 230^th^, 231^st^ and 237^th^ characters are ordered in the second analysis. Other than the ordering of those 40 characters the analyses were identical.

Tree-space was searched using the advanced search methods in TNT, namely: sectorial search, tree fusion, ratchet and drift, for 1,000 random addition replicates. The default settings for the advanced search methods were changed to increase the iterations of each method per replicate: now 100 sectorial search drifting cycles, 100 ratchet iterations, 100 drift cycles and 100 rounds of tree fusion per replicate. This tree-space search procedure was repeated for five different random start seeds. All characters were treated with equal weight. Character polarity was determined with reference to a pre-defined non-crocodylomorph outgroup taxon (*Postosuchus kirkpatricki*). Nodal support was evaluated using non-parametric bootstrapping [73] with 1000 replicates, using TBR searching.

## Results

### Systematic Palaeontology

Superorder Crocodylomorpha Hay, 1930 [74] (*sensu* Walker, 1970) [75].

Infraorder Thalattosuchia Fraas, 1901 [76] (*sensu* Young & Andrade, 2009) [10].

Family Metriorhynchidae Fitzinger, 1843 [77] (*sensu* Young & Andrade, 2009) [10].

Subfamily Geosaurinae Lydekker, 1889 [78] (*sensu* Young & Andrade, 2009) [10].

Tribe Geosaurini Lydekker, 1889 [78] (*sensu* Cau & Fanti, 2011) [12].

#### Type genus

*Geosaurus* Cuvier, 1824 [50].

#### Diagnosis

Metriorhynchid crocodylomorphs with the following unique combination of characters (autapomorphic characters are indicated by an asterisk): high absolute tooth-crown apicobasal length (in some species exceeding 12 centimetres)*; contiguous row of true denticles along the mesial and distal carinae of the teeth*; bicarinate serrated dentition; the inflexion point of the prefrontal lateral margin (in dorsal view) is directed posteriorly at an angle less of 70 degrees or less from the anteroposterior axis of the skull*; acute angle between the medial and the posterolateral processes of the frontal; supratemporal fenestrae enlarged, in dorsal view the posterolateral corner extends further posterior to the intertemporal bar*; all dentary interalveolar distances are very small (always less than a quarter the length of the immediate alveoli)*; dentary tooth-row is ventrally displaced relative to the jaw joint; humerus shaft greatly reduced, contributing less than 25% of total humeral length.

#### Phylogenetic definition

The least inclusive clade consisting of *Geosaurus giganteus*, *Dakosaurus maximus* and *Torvoneustes carpenteri* (*sensu* Cau & Fanti [12]).

*Dakosaurus* Quenstedt, 1856 [32].

#### Type species

*Geosaurus maximus* Plieninger, 1846 [23] (following Recommendation 67B of the ICZN Code).

#### Referred species

*D. andiniensis* Vignaud & Gasparini, 1996 [22].

#### Etymology

“Biter lizard”. *Dakos* (δάχος from Quenstedt [33]:785) is derived from the Ancient Greek ‘to bite’ (δάχυω). Furthermore, Quenstedt ([39]:182) places δάχος in parentheses beside Beisser, the German for biter. While –*saurus* is the latinised version of *sauros*, the Ancient Greek for lizard. Note that Wilkinson *et al*. [18] incorrectly translated *Dakos* as meaning “tearing”.

#### Geological range

Upper Kimmeridgian to lower Berriasian.

#### Geographical range

Europe (England, France, Germany and Switzerland) and South America (Argentina).

#### Geographical note

Possible *Dakosaurus* remains have been found at Khoroshevskii Island, in the Volga region of Russia [79]. They consist of a vertebra and metatarsal from upper Tithonian or lower Berriasian deposits. The possible referral to *Dakosaurus* is presumably due to their large size. However, as there are currently no vertebral or metatarsal *Dakosaurus* autapomorphies this referral cannot be substantiated. The taxonomic affinities of the two fragmentary skull specimens from the Kimmeridgian of Mexico are in question due to newly discovered metriorhynchine specimens from the early Tithonian of Mexico (see discussion; [80]).

#### Spelling

Quenstedt [31], [32] used the spelling, *Dakosaurus* for the genus. However, there has been a question around the transliteration of the Greek letter *χ* into the Latin letters *c* and *k*. During the latter half of the 19^th^ century and the first half of the 20^th^ century *Dacosaurus* was the predominant spelling [3]–[5], [36], [40], [41], [44], [81], [82]. From the latter half of the 20^th^ century onwards the original spelling, *Dakosaurus*, became dominant [1], [2], [10]–[20], [22], [26]–[28], [46], [60]. The first use of the “*c*” spelling was by Sauvage ([44]:380), while Lydekker ([3]:92) was the first to explicitly state that the original spelling had been amended to *Dacosaurus*. However, under the ICZN Code (Article 32.5) an incorrect original spelling cannot be corrected solely on the grounds that it was incorrectly transliterated or latinized. As such, the genus is properly spelt *Dakosaurus*.

#### Emended diagnosis

Metriorhynchid crocodylomorph with the following unique combination of characters (autapomorphic characters are indicated by an asterisk): large robust teeth, with moderate to strong mediolateral compression; carinae formed by a keel and true macroscopic denticles (macroziphodonty, all dimensions exceed 300 µm)*; tooth enamel ornamentation is inconspicuous, visible under SEM and comprising an anastomosed pattern; rostrum proportionately short (brevirostrine, less than 55% of basicranial length), dorsoventrally tall with a convex dorsal margin (oreinirostral)*, and in dorsal view has a distinctly wide and blunt, “bullet” shape (amblygnathous)*; separation between premaxilla and nasal half, or less than half, the midline length of the premaxilla; aligned set of large neurovascular foramina on the maxilla extending posteroventrally from the preorbital fossa (not homologous to the archosaurian antorbital fenestra [8], [9]) *; in dorsal view, the lateral margins of the prefrontals have an inflexion point directed posteriorly at an angle of approximately 50 degrees from the anteroposterior axis of the skull*; acute angle (between 60 and 45 degrees depending on species) between the medial and the posterolateral processes of the frontal; the supratemporal fossae (intratemporal flange) reach the minimum interorbital distance; ventral margin of dentary sharply rises dorsally at the anterior tip*; very short mandibular symphysis (only one third of dentary tooth-row adjacent to the symphysis)*; surangulodentary groove has a well-developed foramen at the dentary terminus*; surangular anteroposteriorly short, terminates posterior to the anterior margin of the orbit.

*Dakosaurus maximus* (Plieninger, 1846) [23] Quenstedt, 1856 [32].

1843 *Megalosaurus* sp. –Quenstedt, p. 493. [30]

v* 1846 *Geosaurus maximus* sp. nov. – Plieninger, p. 150, Taf. 3 Figure 2. [23]

v 1849 *Geosaurus maximus* Plieninger – Plieninger, p. 252, Taf. 1 Figure 7. [29]

v 1852 *Megalosaurus* sp. –Quenstedt, p. 112, Taf. 8 Figure 4 [31]

v* 1853 *Liodon paradoxus* sp. nov. – Wagner, p.263, Taf. 3 Figures 9–13. [35]

v 1856 *Dakosaurus maximus* (Plieninger) gen. et comb. nov. –Quenstedt, p. 131. [32]

v 1858 *Dakosaurus maximus* (Plieninger) –Quenstedt, p. 785. [33]

v* 1869 *Dakosaurus lissocephalus* sp. nov. – Seeley, p. 92. [42]

* 1871 *Leiodon primaevum* sp. nov. – Sauvage, p. 141. [43]

1873 *Dacosaurus primaevus* (Sauvage) comb. nov. et unjust.emend. – Sauvage, p. 380, pl. 7 Fig. 3–5. (*sic*) [44]

* 1881 *Teleosaurus suprajurensis* sp. nov. – Schlosser. [36]

v 1885 *Dakosaurus maximus* (Plieninger) –Quenstedt, p. 182. [39]

v* 1885 *Dakosaurus gracilis* sp. nov. –Quenstedt, p. 184. [39]

v 1888 *Dacosaurus maximus* (Plieninger) – Lydekker, p. 92–94, Figure 13. (*sic*) [3]

v 1902 *Dacosaurus maximus* (Plieninger) – Fraas, p. 7, Fig. 1–2 Taf. 1, Taf. 2 Fig. 2–11, Taf 3–4 (*sic*) [4]

v 1902 *Dacosaurus paradoxus* (Wagner) comb. nov. – Fraas, Tafel 2, Fig. 1, 12–13 (*sic*) [4]

v 1902 *Dacosaurus suprajurensis* (Schlosser) comb. nov. – Fraas, p. 20 (*sic*) [4]

1925 *Dakosaurus maximus* (Plieninger) – Huene, p. 600, plate 26 Figure 57. [83]

v 1973 *Dakosaurus maximus* (Plieninger) – Steel, p. 42, Figure 18 (6, 11). [46]

v 2009 *Dakosaurus maximus* (Plieninger) – Young & Andrade, p. 555, Fig. 5. [10]

v *2009 Dakosaurus lissocephalus* (Seeley) – Young & Andrade, p. 579. [10]

v 2010 *Dakosaurus maximus* (Plieninger) – Young *et al*., p. 804, Fig. 4, 6. [1]

v *2010 Dakosaurus lissocephalus* (Seeley) – Young *et al*., p. 859. [1]

v 2010 *Dakosaurus maximus* (Plieninger) – Andrade *et al*., p., Fig. 4. [11]

#### Holotype

Isolated tooth, the location of which is unknown and is presumed lost.

#### Etymology

‘Greatest biter lizard’. From the Latin *maximus*, meaning largest/greatest.

#### Holotype locality and horizon

Schnaitheim, Baden-Württemberg, Germany. Mergelstätten Formation. *Hybonoticeras beckeri* Sub-Mediterranean ammonite Zone, upper Kimmeridgian, Upper Jurassic.

#### Neotype

SMNS 8203– incomplete skull and mandible (first suggested by Young & Andrade [10]).

#### Neotype locality and horizon

Staufen bei Giengen, Baden-Württemberg, Germany. Mergelstätten Formation. *Hybonoticeras beckeri* Sub-Mediterranean ammonite Zone, upper Kimmeridgian, Upper Jurassic.

#### Designation of neotype

Herein we formally designate SMNS 8203 as the neotype of *Dakosaurus maximus*. In order to be in full accordance of Article 75 of the ICZN Code, in particular Article 75.3, we make the following statements:

1. This designation is made with the express purpose of clarifying the taxonomic status of *Dakosaurus maximus*.
2. Our statement of the characters that we regard as differentiating *Dakosaurus maximus* from other taxa is given by the species diagnosis below.
3. The neotype can be recognised through both the description below and Figs. 2, 3, and 4.
4. The holotype (an isolated tooth) cannot be located and is presumed lost. The type was described in the 1846 [23], and there is no known documentation to suggest which institution the holotype was given to, assuming the specimen was curated in a scientific institution.
5. The holotype is an isolated tooth, from the description and figure given by Plieninger [23] show it was both robust and macroziphodont. As such, the neotype is consistent with what is known of the former name-bearing type.
6. While the neotype is not from the same locality as the holotype, both are from the same Sub-Mediterranean ammonite Zone. The two localities are little over 10 km from one another.
7. The neotype is the property of a recognized scientific institution, SMNS, which maintains a research collection with proper facilities for preserving name-bearing types, and is accessible for study.

#### Geological range

Upper Kimmeridgian (*A*. *eudoxus* Sub-Boreal ammonite Zone) to lower Tithonian (*H*. *hybonotum* Sub-Mediterranean ammonite Zone).

#### Geographical range

Cambridgeshire, England; Pas-de-Calais, France; Baden-Württemberg & Bayern, Germany; Canton Solothurn, Switzerland.

#### Referred specimens

NHMUK PV OR33186, NHMUK PV OR35766, NHMUK PV OR35835, NHMUK PV OR35836, NHMUK PV OR35837, SMNS 51494, SMNS 55420, SMNS 80148: isolated teeth from Schnaitheim (*H. beckeri* Sub-Mediterranean Zone); SMNS 81793: isolated tooth from Nusplingen, Baden-Württemberg, Germany (*H. beckeri* Sub-Mediterranean Zone); SMNS 10819a, b: broken and dorsoventrally compressed skull from Sontheim an der Brenz, Baden-Württemberg, Germany; SMNS 82043: right mandibular ramus in lithographic limestone, from Painten, Bayern, Germany; CAMSM J29419: incomplete dorsoventrally crushed skull (holotype of *D. lissocephalus*) from Ely, Cambridgeshire, England, Lower Kimmeridge Clay Formation (*A. eudoxus* Sub-Boreal Zone); NHMUK PV OR20283: an isolated tooth, also from Ely, England; NHMUK PV OR32414, SMNS 57210: isolated teeth from Boulogne-sur-Mer, Pas-de-Calais, France, Argiles de Châtillon Formation (*A. autissiodorensis* Sub-Boreal Zone); SMNS 56999: partial maxilla also from Boulogne-sur-Mer, France; NMS 7009: isolated tooth from Canton Solothurn, Switzerland, Reuchenette Formation (upper Kimmeridgian); JME-SOS4577, JME-SOS2535: isolated teeth from Schernfeld, Bayern, Germany, Solnhofen Formation (lower Tithonian; *H. hybonotum* Sub-Mediterranean Zone).

#### Diagnosis

Metriorhynchid crocodylomorph within the genus *Dakosaurus* with four autapomorphic characters: 1) wear facets on the mesial and distal edges of the crown that obliterate the carinae; 2) thin lamina of bone projecting from the lateral alveolar margin of the premaxilla (“premaxillary lateral plates”); 3) maxilla is strongly ornamented, with most of the element covered in long deep grooves and long raised ridges orientated to the long axis of the skull, but with the alveolar margin largely smooth; 4) in the posterior half of the dentary, there are laminae of bone projecting from the lateral and medial dentary alveolar margins (“dentary lateral and medial plates”). Note that the preservation of *Dakosaurus andiniensis* makes it difficult to assess whether it also possesses characteristics one and two.

#### Body length estimate

The largest known specimen of *Dakosaurus maximus* is the isolated mandible SMNS 82043 (Fig. 6), which is 87.5 cm in length. Using the ratio of basicranial length to mandibular length in “*Metriorhynchus*” *brachyrhynchus* as a guide (NHMUK PV R3804: the most three-dimensionally preserved NHMUK specimen with a complete skull and mandible; mandible length = 80.9 cm, basicranial length = 76.8 cm) and assuming that the basicranium and mandibles of *D*. *maximus* scale in the same proportions, SMNS 82043 is estimated as having a basicranial length of 83.1 cm. This gives a total body length estimate of 4.49 m, using the Young *et al*. [14] equations. This is slightly greater than the body length given in Young *et al*. [14], however that was based on an estimated length of the neotype SMNS 8203, which they found to be 4.28 m long.

### Description and Comparisons

#### Skull: general comments

Many cranial and mandibular bones are preserved in the neotype (SMNS 8203; Figs. 2, 3, 4), referred cranial elements (SMNS 10819, Fig. 5; SMNS 56999, Fig. 7) and the referred mandible (SMNS 82043, Fig. 6), but several other bones are not preserved and are thus unknown in *D*. *maximus*. These include: jugals, lacrimals, frontal, parietal, postorbitals, squamosals, quadrates, braincase, occiput, pterygoids and ectopterygoids. Overall, the skull has a shape very similar to that of *D. andiniensis*: they both have a short, broad “bullet”-shaped snout (amblygnathous), which is very robust and has a convex upper margin (oreinirostral) (see Fig. 3).

#### Premaxilla and external nares

The premaxilla bears three alveoli, as with all other metriorhynchids [4], [5], [10]. The ornamentation on the lateral surface of the premaxilla is composed of numerous large elliptic pits, and the bone is slightly convex laterally (SMNS 8203, Figs. 2, 3, 4; SMNS 10819a, Fig. 5A). The premaxillae completely enclose the external nares, as in all thalattosuchians with the exception of *Cricosaurus macrospondylus* (in which the maxilla also contributes [84]). Along the posterior margin of the premaxilla, the posterodorsal process contacts the anterior margin of the maxilla. This suture forms a broad ‘U’-shape in dorsal view (much like *D. andiniensis* [19], [20]), rather than a posteriorly pointed ‘V’-shape (such as in *Metriorhynchus superciliosus*, *Gracilineustes leedsi* and “*Metriorhynchus*” *brachyrhynchus* [5]). As with almost all thalattosuchians, there is no premaxilla-nasal contact [5], as these bones are separated by the maxilla. The palatal surface of the premaxilla in *D. maximus* is unknown.

Along the lateral margin of the premaxilla there is a thin lamina of bone that covers the basal portion of the teeth (SMNS 8203; Fig. 4A, 4B). This morphology is somewhat similar to the ‘lateral plates’ observed in sauropod dinosaurs (e.g. *Diplodocus longus* CM 11161). Finite element analysis modelling of this skull by Young *et al.* [85] found that, regardless of the feeding behaviour simulated, high stresses occurred at the tooth bases and the ‘lateral plates’ during feeding. These results support the hypothesis that ‘lateral plates’ help to dissipate feeding-induced stresses acting on the bases of adjacent teeth [85], which we hypothesise was their function in *D. maximus*. *Dakosaurus*/*Plesiosuchus manselii* (NHMUK PV OR40103) lacks these structures (as do all other known metriorhynchids [5], [64]), while the state of preservation makes determining this morphology difficult in *Dakosaurus andiniensis* [20]. Therefore, we regard them as an autapomorphy of *D*. *maximus*, but note that future discoveries may reveal that they are more widely distributed among *Dakosaurus* species.

In *Dakosaurus maximus* there is a single, anterodorsally facing external naris (Figs. 2, 3, 5). This condition is also seen in most other metriorhynchids, such as *Dakosaurus andiniensis* [19], [20], *Metriorhynchus superciliosus* (e.g. NHMUK PV R6859, NHMUK PV R6860), *Gracilineustes leedsi* (e.g. NHMUK PV R3014, NHMUK PV R3015) and “*Metriorhynchus*” *brachyrhynchus* (NHMUK PV R3804). The members of the subclade Rhacheosaurini have a different morphology, in which the naris is divided by a premaxillary septum and is anterodorsally and laterally oriented (e.g. *Rhacheosaurus gracilis* NHMUK PV R3948; *Cricosaurus suevicus* SMNS 9808).

#### Maxilla

The maxillae are similar to those of *Dakosaurus andiniensis*, as they are noticeably short, high and subtriangular in lateral view [19], [20]. One difference is that the maxillae of *Dakosaurus maximus* are not as high dorsoventrally (compare Figs. 1, 2). Gasparini *et al*. [19] compared the ratio of snout height to snout length among various crocodylomorphs, and they found that *D. maximus* had a ratio of 0.15, whereas *D. andiniensis* had an even greater ratio of 0.36. This was in marked contrast to other thalattosuchians, as longirostrine species had a ratio of 0.04–0.05 (e.g. *Steneosaurus bollensis*, *Pelagosaurus typus* and *Cricosaurus araucanensis*) while mesorostrine metriorhynchids had a ratio of 0.08–0.09 (*Metriorhynchus superciliosus* and “*Metriorhynchus*” *casamiquelai*).

The maxillae of *D. maximus* bear 13 alveoli (SMNS 8203, Fig. 2) [4]. Like the premaxillae, the maxillae are slightly convex laterally. The ornamentation of the lateral surface in *Dakosaurus maximus* is distinctive, as it noticeably differs across the element (SMNS 8203, Figs. 2, 3, 4; SMNS 10819a, Fig. 5; SMNS 56999, Fig. 7). Near the premaxilla-maxilla suture, the ornamentation is very similar to that on the premaxilla (numerous large elliptical pits). On most of the element, and in particular closer to the maxillary midline and maxillonasal suture, the surface is covered in long deep grooves and long raised ridges orientated parallel to the long axis of the skull. Approaching the alveolar margin, the ornamentation becomes more subtle, composed of ridges of low-relief arranged in an anastomosed pattern, creating a fabric of crests over the surface. Almost all of the maxillary foramina exit out on to the anastomosed region of the maxilla. The maxillae of *D. andiniensis* [20], *Torvoneustes carpenteri* [18] *Cricosaurus schroederi* and *C. araucanensis* (see Figure 5 in [10]), and *Geosaurus giganteus* [10] are largely smooth, with elliptical pits that are shallow and fairly indistinct.

Along the dorsal midline of the skull the left and right maxillae meet at a long suture, and terminate at the anterior margin of the nasal. The maxillonasal suture begins at the skull midline and forms an anteriorly pointed ‘V’-shape, as is also the case in *D*. *andiniensis* and other metriorhynchids [5], [10], [19], [20], [64]. With the jugals and lacrimals either missing or not preserved in all specimens of *D. maximus*, the nature of their contact with the maxilla cannot be determined. Similarly, the contribution the maxilla made to the preorbital fossa is unknown.

The alveolar margin of the maxilla is poorly preserved in the neotype (SMNS 8203, Fig. 4B). As such, the presence or absence of ‘lateral plates’, like those seen on the premaxilla, is unknown. In the referred specimen SMNS 10819a (Fig. 5), the alveolar margin is also partially damaged, although the medial section of the maxilla does not seem to exhibit the ‘plates’. In palatal view, the maxillae of the neotype (SMNS 8203) are very poorly preserved. However, in SMNS 10819a the maxillae suture along the midline forming part of the secondary palate (Fig. 5B, 5C). The maxillopalatine suture is not preserved in any specimen. However, the midline terminus of the maxillopalatine suture must have been posterior to the eight anterior maxillary alveoli; as those teeth are preserved in SMNS 10819. This is comparable to other Geosaurini genera, except *Plesiosuchus* (i.e. *Dakosaurus/Plesiosuchus manselii*), where the maxillopalatine suture terminates level to the fourth maxillary alveolus (Table 1).

#### Nasals

The nasals are large, paired, unfused elements (Figs. 2, 3). In dorsal view they are subtriangular in shape and broad, like in all thalattosuchians [5]. Along the midline the dorsal surface of the nasals is deeply trenched, with a steep longitudinal depression (Fig. 3B), a characteristic shared by all metriorhynchoids [5], [64] (e.g. *Pelagosaurus typus* NHMUK PV OR32599; *Teleidosaurus calvadosii* NHMUK PV R2681; *Eoneustes gaudryi* NHMUK PV R3353; *Metriorhynchus superciliosus* NHMUK PV R6859, NHMUK PV R6860; *Gracilineustes leedsi* NHMUK PV R3014, NHMUK PV R3015; “*Metriorhynchus*” *brachyrhynchus* NHMUK PV R3804). The anterior margin forms an acute angle along its border with the maxilla. Most of the dorsal and lateral surfaces of the nasals are well ornamented, with a pitted pattern. This is in contrast with other species in Geosaurini, which have nasals that are largely smooth (*D. andiniensis* [19], [20]; *Torvoneustes carpenteri* [18]; *Geosaurus giganteus* and *G. grandis* [10]; *Plesiosuchus manselii* NHMUK PV OR40103).

Although the frontal and lacrimals are missing, and the prefrontals are poorly preserved, it is possible to determine where these bones would have contacted the nasals by using the well preserved skull of *D. andiniensis* as a guide [19], [20]. Along its posterior margin, the nasals would have contacted the frontal and prefrontals. The two dorsoposterior processes would have contacted the frontal medially, and the prefrontals laterally. Between the dorsoposterior and lateroposterior processes, the nasal would have contacted the prefrontals. Ventral to the lateroposterior processes the nasal would have contacted the lacrimal and contributed to the preorbital fossa margin. The presence of distinct nasal lateroposterior processes is a metriorhynchid apomorphy (see Young *et al*. [1]: Figs 4A, 6 for a reconstruction of *Teleidosaurus calvadosii* and a photograph of *Eoneustes gaudryi* respectively, as these basal metriorhynchoids lack these processes).

#### Prefrontal

Only the left prefrontal is present in SMNS 8203, and it is poorly preserved (Figs. 2, 3). As with other metriorhynchids, the left prefrontal has an enlarged, expanded dorsal surface, and therefore it is widely visible on the skull roof in dorsal view [1], [4], [5]. Furthermore, enough of the prefrontal is preserved to show that this bone would have been expanded laterally to overhang the orbits. The prefrontal would have extended onto the lateral surface of the snout, between the orbit and the preorbital fenestra. However, this region of the prefrontal (the descending process) is also poorly preserved.

#### Fenestrae and fossae

Due to the poor preservation of the neotype and referred specimens, the various cranial fossae and fenestrae characteristic of metriorhynchids are difficult to recognise. No specimen preserves the preorbital fossae (not homologous to the archosaurian antorbital fossae [8], [9]). These are typically elliptical and obliquely orientated in metriorhynchids, and much longer than higher [4], [5].

The exit for the post-temporal openings ( =  post-temporal fenestra; post-temporal foramen) on the occipital surface of the skull cannot be determined for SMNS 10819 due to its preservation.

Other fenestrae are not preserved. Nevertheless, most thalattosuchians either lack or have a highly reduced naso-oral fenestra ( = incisive foramen, *foramen incisivum*); and metriorhynchids lack external mandibular fenestrae [4], [5]. Neither the infratemporal fenestrae ( = laterotemporal fenestrae) nor the lacrimal-prefrontal fossae [10] are preserved.

#### Mandible: general comments

The preservation of the left mandible is poor in the neotype (SMNS 8203; Fig. 2). The referred specimen SMNS 82043 is a much better preserved left ramus embedded in lithographic limestone (Fig. 6). The mandible of SMNS 8203 has twelve alveoli [4]. The symphysis cannot be discerned in SMNS 82043 (because the medial surface of this specimen is obscured by matrix), while in SMNS 8203 it is very short, extending as far posteriorly as the 4^th^ dentary alveolus. Both specimens preserve the lateral mandibular groove ( =  surangulodentary groove) on the lateral surface of dentary and surangular. Assuming the preorbital fenestra was in the same position as in *D. andiniensis*; then the surangulodentary groove would have extended further anteriorly than it. This is in contrast to *Geosaurus giganteus*, in which the surangulodentary groove and the preorbital fenestra reach the same relative position [10]. The groove is deeply excavated, and in SMNS 8203 a large foramen is present at the dentary terminus. The deep excavation of the surangulodentary groove is also present in *Dakosaurus*/*Plesiosuchus manselii* (see below), and although the mandible is more poorly preserved in *Geosaurus giganteus* it too seems to have a deeply excavated groove [10]. This groove is present, but shallower, in all thalattosuchians, but can be easily obscured by post-mortem deformation (e.g. “*Metriorhynchus*” *brachyrhynchus* NHMUK PV R3804; *Gracilineustes* cf. *acutus* CAMSM J29475). As mentioned above, no external mandibular fenestra is evident.

As discussed by Young *et al*. [2], the mandible within Geosaurinae undergoes a characteristic shape change related to an increase in gape. All metriorhynchids exhibit some ventral displacement of the dentary tooth row, such that the coronoid process is located above the plane of the tooth row and is on the same plane as both the retroarticular process and the glenoid fossa (*Metriorhynchus superciliosus*: GLAHM V1141; *Gracilineustes leedsi*: NHMUK PV R3014, NHMUK PV R3015; “*Metriorhynchus*” *brachyrhynchus*: GLAHM V995, NHMUK PV R3804). The coronoid process is a metriorhynchid apomorphy, and basal non-metriorhynchid metriorhynchoids such as *Teleidosaurus calvadosii* lack this structure (NHMUK PV R2681, Figure 6 in Young *et al*. [1]). However, in the derived metriorhynchid clade of the “Mr Leeds’ specimen” (a new genus and species described by [2]) and Geosaurini, there is further ventral displacement of the dentary tooth row [2]. In this subclade, the coronoid process is no longer on the same plane as the jaw joint, but is ventrally displaced. In addition, the angular continues to rise dorsally posterior to the coronoid process. The isolated mandible SMNS 82043 shows that *D. maximus* also has this characteristic mandibular shape. *Dakosaurus andiniensis* is unique in having a greatly expanded coronoid process (see Figure 2 in Pol & Gasparini [20]).

#### Dentary

In the neotype only the dentary is well-preserved, but not at its articulation with the surangular and angular (Fig. 2). However, in SMNS 82043 the sutures between the dentary and both elements are easily identified (Fig. 6; although some of the original surface texture has been eroded). The dentary is heavily pitted, especially at the anterior end. Along the dorsal margin of the dentary there is no evidence of festooning. The ventral margin is also straight, except for its anteriormost part where the margin rises anterodorsally, although not as sharply as in *D. andiniensis* [19], [20].

The symphysis is very short in this species. The neotype and only specimen which has the symphyseal articulation facet preserved, has only four dentary teeth adjacent (Fig. 3). This is in marked contrast to other geosaurins, *Geosaurus* has at most eight, while *Plesiosuchus* has nine (Table 1). This results in only one third of the dentary teeth being adjacent to the symphysis in *D. maximus*, whereas in other geosaurins it ranges from 61–71% (Table 1).

#### Angular and surangular

The angular and surangular are strongly sutured along their entire border, with the angular forming the ventral half of the posterior mandible and the surangular the dorsal half (Fig. 6). The angular ventral margin is gently concave, curving dorsally towards the jaw joint. The angular terminates significantly higher than the dentary tooth row. As with the angular the surangular gentle curves dorsally, and possesses a well-developed coronoid process. The dentary–surangular suture projects anteroventrally. As the medial side of the mandible is not exposed, it was not possible to verify the actual extension of the medial ramus of the surangular.

#### Dentition: tooth morphology

The most commonly discovered elements of *Dakosaurus maximus* are isolated tooth crowns (e.g., Fig. 8). These can be identified as belonging to *D*. *maximus* because the *in situ* teeth of the neotype and referred specimens have distinctive, autapomorphic morphologies (e.g. SMNS 8203, Fig. 4; SMNS 10819a, Fig. 5; SMNS 82043, Fig. 6). Each tooth shows a caniniform morphology: they are single cusped and mediolaterally compressed. No constriction is present at the crown/root junction, but the boundary is evident through colour and texture. The crowns are curved lingually, but do not curve throughout, only at the middle and apical sections. The basal sections are wider labiolingually, creating a more sub-circular to slightly ovoid cross-section. The teeth lack the distinctive apicobasal faceting observed on the labial surface of contemporaneous *Geosaurus* species [10], [11]. The crowns are robust and large in comparison to the teeth of other thalattosuchians (e.g. *Steneosaurus leedsi*: NHMUK PV R3806; *Metriorhynchus superciliosus*: GLAHM V1141; “*Metriorhynchus*” *brachyrhynchus*: NHMUK PV R3804) [5]. Cingula and accessory cusps are absent, as in all thalattosuchians (e.g. *Steneosaurus leedsi*: NHMUK PV R3806; *Metriorhynchus superciliosus*: GLAHM V1141; “*Metriorhynchus*” *brachyrhynchus*: NHMUK PV R3804) [5], [64]). Based on the neotype, this species has a dental formula per ramus of three premaxillary, 13 maxillary and 12 dentary teeth. This tooth count is very slightly lower than that of other Geosaurini genera, which have 14 maxillary and 13 dentary teeth (see Table 1). *Dakosaurus andiniensis* has a slightly shorter tooth row than *D. maximus* (10/11 maxillary teeth and 12 dentary teeth), giving it the shortest tooth row of any thalattosuchian. Most mesorostrine/longirostrine thalattosuchians have an upper or lower jaw tooth count of between 24 and 45 teeth [5], [51], [64].

#### Dentition: ornamentation and carinae

Surface ornamentation is light, composed of microscopic ridges in an arranged an anastomosed pattern, creating a fabric of crests over the surface [11]. Given the small size of these ridges, the overall appearance of the tooth surface is reasonably smooth. This morphology is similar to that observed in *Geosaurus giganteus* [10], [11].

The teeth have carinae comprised of both denticles and a keel, as in true ziphodont teeth [11]. The carinae are well-defined, extending from the base to apex of the crown on both the mesial and distal margins. Denticles extend contiguously along the entire length of the preserved carinae. Overall, the denticles have a fairly consistent height (isometric), but their shape varies (poorly isomorphic, rounded and never rectangular or square). The individual denticles of NHMUK PV OR35766 are large, with maximum measurements of 425 µm × 330 µm × 675 µm (apicobasal length, height, and transverse width respectively); these dimensions are reasonably similar to those of *D. andiniensis* (see Table 2 in Andrade *et al*. [11]). The profile of the denticles is rounded in lingual view, but the serrations bear a sharp cutting edge (the keel) on the distal and mesial margins. This morphology is also observed in other members of Geosaurini [2], [11], [20]. Metriorhynchines lack any carinal serrations, whereas basal geosaurines (i.e. those not in the subclade Geosaurini) possess incipient microdenticles that do not proceed contiguously along the entire carina [2].

#### Dentition: wear and occlusion

The macroscopic and microscopic wear of *Dakosaurus maximus* teeth were described in detail by Young *et al*. [17]. In summary, isolated (NHMUK and SMNS specimens) and *in-situ* (SMNS 8203, Figs. 2, 3, 4; SMNS 10819a, Fig. 5; and SMNS 82043, Fig. 6) *D. maximus* teeth, of different size and position, exhibit three distinct types of macroscopic wear features. The first is spalled enamel near the apex; second, occlusal wear along the mesial and distal margins (i.e. along the carinae) and third, a wear facet at the base of the crown which is semi-circular.

At the apex, enamel spalling is frequently observed. It can be present on either the labial or lingual surface, and can be extensive (e.g. SMNS 9808; Fig. 8A). The spalled surface begins at the crown apex and proceeds basally, generally forming an ovoid or triangular facet. The teeth of tyrannosaurid dinosaurs, which are reminiscent in size and shape to those of *Dakosaurus*, also exhibit enamel spalling that is interpreted as tooth-food abrasion [86]. Interestingly, extreme spalling and complete tooth breakage patterns are observed in extant aquatic amniotes, in particular the killer whale *Orcinus orca* [87], [88]. This pattern spalling and crown breakage is observed in killer whale populations that are associated with the consumption of large prey items (macrophagy), specifically predation of sharks [88].

The second type of macroscopic wear proceeds along the edges of the mesial and distal surfaces of the teeth (Fig. 8) [17]. The mesial/distal macrowear extends from the apex and terminates at a variable distance towards the base, and in some isolated crowns the wear can extend along the entire length of the carinae (SMNS 9808, Fig. 8A). Interestingly, this type of wear obliterates the carina (keel and denticles). Similar wear facets, which as in *Dakosaurus* are elongated, elliptical, and follow the long axis of the tooth, have been observed in tyrannosaurid dinosaurs and interpreted as representing tooth-on-tooth attritional wear [86]. Young *et al*. [17] hypothesized that these facets in were formed as a result of tooth-on-tooth occlusion, namely that during occlusion the upper and lower jaw teeth would have met each other mesiodistally with carinae-to-carinae contact (i.e. the teeth would have fit in between each other when the jaws closed; much like extant false killer whales *Pseudorca crassidens* [17]). Examination of these carinal wear facets under scanning electron microscopy reveals the presence of striations that are regularly oriented, large, and restricted to the wear facet itself [17]. Similar striations are also present on the elliptical wear facets of tyrannosaurid teeth, as well as those regions on the teeth of lions that make contact with the teeth of the opposing tooth row during shearing [86].

The hypothesis that the teeth of the upper and lower jaws contacted one another mesiodistally along their carinae during occlusion is supported by the arrangement of the teeth. The complete and articulated skull and mandible of *Dakosaurus andiniensis* exhibits *in situ* vertically oriented tooth crowns which are closely packed (see Fig. 1) [19], [20]. As of yet, there is no complete *Dakosaurus maximus* cranial material comparable to that of the well-preserved skull of *D. andiniensis*, which prevents direct observation of occlusion in this species. However, the presence of reception pits on the premaxilla, maxilla and dentary (SMNS 8203, Fig. 4A; SMNS 10819a, Fig. 5A; and SMNS 82043, Fig. 6C, 6D) indicates that the teeth were indeed tightly packed, oriented vertically, and would have repeatedly contacted the opposing jaw bone during occlusion [17].

*Plesiosuchus* Owen, 1884 [52].

#### Type species

*Steneosaurus manselii* Hulke, 1870 [24] (following Recommendation 67B of the ICZN Code).

#### Etymology

“Near crocodile”. *Plesios* (πλεσιος) Greek for near/close to, while *suchus* (συχος) means ‘crocodile’, and is the Latinised form of the Ancient Greek for an Egyptian species (according to Owen [45]). Owen [52] considered *Plesiosuchus* to be nearer to, morphologically, extant crocodylians than *Steneosaurus*; hence the name.

#### Geological range

Upper Kimmeridgian to lower Tithonian.

#### Geographical range

England, and possibly also Spain [28].

#### Diagnosis

Same as the only known species.

*Plesiosuchus manselii* (Hulke, 1870) [24] Owen, 1884 [52].

v 1869 *Pliosaurus trochanterius* (Owen) – Owen, p.7, pl. 3 [54]

v 1867–69 *Metriorhynchus* (von Meyer) – Eudes-Deslongchamps, p. 329 [58]

v 1869 *Steneosaurus rostro-minor* (Geoffroy Saint-Hilaire) – Hulke, p. 390, pl. 17–18 [48]

v* 1870 *Steneosaurus manselii* sp. nov. – Hulke, p. 167, pl. 9 [24]

v 1884 *Plesiosuchus manselii* (Hulke) gen. et comb. nov. – Owen, p. 153 Figure 3 (2) [52]

v 1849–84 *Plesiosuchus mansellii* (Hulke) – Owen, p. 146, pl. 20 Figures 1–4 (*sic*) [45]

v *1885 Metriorhynchus* sp. (von Meyer) – Woodward, p. 502 [53]

v *1885 Dakosaurus manselii* (Hulke) comb. nov. – Woodward, p. 503 [53]

v 1888 *Dacosaurus maximus* (Plieninger) – Lydekker, p. 93 (*sic*) [3]

v 1888 *Machimosaurus mosae* (Sauvage & Lienard) – Lydekker, p. 104 [3]

v *1890 Machimosaurus mosae* (Sauvage & Lienard) – Lydekker, p. 286 [66]

v 1902 *Dacosaurus Manselii* (Hulke) – Fraas, p. 20, Fig. 3–6 (*sic*) [4]

v 1973 *Dakosaurus mansellii* (Hulke) – Steel, p. 42 (*sic*) [46]

v 1995 *Colymbosaurus trochanterius* (Owen) – Benton & Spencer, p. 189, Figure 7.5 [61]

v 1996 *Dakosaurus manseli* (Hulke) – Grange & Benton, p. 509 [47]

v *2009 Dakosaurus manselii* (Hulke) – Young & Andrade, p. 560 [10]

v *2010 Dakosaurus manselii* (Hulke) – Young *et al*., p. 819 [1]

#### Holotype

NHMUK PV OR40103– incomplete skull (snout and occiput – lacking occipital condyle – with fragments of the supratemporal arches) and isolated right articular. NHMUK PV OR40103a – the right mandibular ramus, some isolated teeth, a humerus, and numerous ribs and vertebrae that are partially or completely imbedded in matrix. Two further specimens may belong to the *P. manselii* holotype: NHMUK PV OR40104– an occipital condyle, and NHMUK PV OR40105– carpal and tarsal bones. Both specimens were donated to the University of Toronto through Professor Ramsay Wright in 1900. From examining the relevant register in the NHMUK Earth Sciences Department, it is possible both specimens were part of NHMUK PV OR40103 (as they are all part of the same acquisition, NHMUK PV OR40103 lacks the occipital condyle and NHMUK PV OR40103a does not include carpal or tarsal bones). Unfortunately, as neither specimen could be located at the Royal Ontario Museum (K. Seymour, 2011 pers. com.) it is impossible to assess whether they too belong to the holotype.

#### Etymology

“Mansel’s near crocodile”. Named after its discoverer, JC Mansel-Pleydell.

#### Type locality and horizon

Kimmeridge Bay, Dorset, England. Lower Kimmeridge Clay Formation. *Aulacostephanus autissiodorensis* Sub-Boreal ammonite Zone, upper Kimmeridgian, Upper Jurassic.

#### Referred specimens

NHMUK PV R1089: incomplete skull (braincase with part of the supratemporal arches) and mandible. Kimmeridge, Dorset, England. Upper Kimmeridge Clay Formation. *Pectinatites wheatleyensis* Sub-Boreal ammonite Zone, lower Tithonian, Upper Jurassic (S. Etches 2011 pers. com.). Two further specimens (K181: isolated teeth, partial maxilla?, partial left mandible, ribs, vertebrae, femur, and K434: right dentary) in the Museum of Jurassic Marine Life (Kimmeridge, England; the Etches Collection is in the process of becoming a museum) are referable to *Plesiosuchus manselii*. The isolated Spanish tooth crown described by Ruiz-Omeñaca *et al*. [28] as *Dakosaurus* sp. shares the same enamel ornamentation pattern, denticles size and lack of wear observed on the teeth of the holotype (MUJA-1004, now referred to cf. *Plesiosuchus manselii*, see below).

#### Specimen note

The specimen NHMUK PV OR40103b, a short series of cervicodorsal vertebrae preserved in matrix, is clearly a thalattosuchian due to its possession of several apomorphies of the group (amphicoelous centra, well developed diapophyseal and parapophyseal processes, no hypapophyses). It does not, however, belong to the same individual as NHMUK PV OR40103/NHMUK PV OR40103a. The vertebrae are much smaller than those of NHMUK PV OR40103a, and the matrix is of a different composition. As such, it is unclear whether NHMUK PV OR40103b belongs to *Plesiosuchus* or another metriorhynchid, and it is here considered Thalattosuchia indeterminate.

#### Diagnosis

Metriorhynchid crocodylomorph with the following unique combination of characters (autapomorphic characters are indicated by an asterisk): large robust teeth, with moderate to strong mediolateral compression; carinae formed by a keel and true microscopic denticles (microziphodonty, dimensions do not exceed 30 0µm); denticles are rectangular-shaped in lingual view*; tooth enamel ornamentation is largely inconspicuous, but there are apicobasally aligned ridges of low-relief*; the mesial margin of some teeth have a pronounced distal curvature*; separation between premaxilla and nasal approximately subequal to the midline length of the premaxilla; in dorsal view, the lateral margins of the prefrontals have an inflexion point directed posteriorly at an angle of approximately 70 degrees from the anteroposterior axis of the skull; palatines are strongly convex with a pronounced ridge along the midline*; in palatal view, the palatine width narrows anteriorly from the suborbital fenestrae to the midline (a distinct elongate triangular shape)*; the maxillopalatine suture midline terminus is level to the fourth maxillary alveolus*; quadrate distal articular surface is not separated into two condyles by a sulcus, and has only a very shallow depression at the centre*; mandibular symphysis long (9 out of 13 dentary teeth are adjacent to the symphysis).

#### Taxonomic Note

As discussed above, this species has a long and complicated taxonomic history, and it has been referred to both its own genus (*Plesiosuchus*) and to *Dakosaurus*. Our phylogenetic analysis, which is reported below, does not find compelling evidence for a monophyletic *Dakosaurus* clade including both *D*./*P*. *manselii* and the *Dakosaurus* type species, *D*. *maximus*. Therefore, we resurrect the genus name *Plesiosuchus* and refer to this species as *P*. *manselii* from here onwards in this monograph.

#### Ontogenetic stage and body length estimate

None of the vertebrae in NHMUK PV OR40103a are well enough preserved to determine the nature of the neurocentral sutures. Amongst crocodylomorphs the fusion of the neurocentral sutures proceeds from the caudal to the cervical vertebrae during ontogeny, with fusion of the cervicals occurring in morphologically mature specimens [89], [90]. This caudal-cervical fusion pattern has been confirmed as occurring in thalattosuchians [2], [91]. Therefore, it is uncertain whether the holotype and referred specimens belong to adults or subadults. Using the body estimation method outlined by Young *et al*. [14], NHMUK PV OR40103 would have been approximately 5.42 m in total length (based upon the above 100 cm basicranial length estimate). While it is difficult to estimate the length of the skull due to the non-continuous nature of the rostrum and occiput pieces, the snout length is 58 cm long. The mandible is approximately 111 cm in total length.

The mandible of NHMUK PV R1089 is 132.2 cm in length. Using the ratio of basicranial length to mandibular length in “*Metriorhynchus*” *brachyrhynchus* NHMUK PV R3804 (as we did for SMNS 82043, see above), NHMUK PV R1089 is estimated as having a basicranial length of 125.5 cm. This gives a total body length estimate of 6.83 m, using the Young *et al*. [14] equations. This is greater than the body length given reported by Young *et al*. [14], however that estimate was based on material we now know is NHMUK PV OR40103 and NHMUK PV R1089. Prior to this study there was confusion surrounding which mandible went with which occiput (due to NHMUK PV R1089 not being labelled). The earlier body length estimate of 5.97 m [14] can be disregarded as an error. This means the body length of *Plesiosuchus manselii* likely falls within the size range of the top predator of the Oxford Clay Sea, the pliosaur *Liopleurodon ferox*. An adult specimen of *L*. *ferox* with a cranial length of ∼126 cm has been estimated to have a total body length of 6.39 m [92], although the largest known *L. ferox* skull has a length of 154 cm (NHMUK PV R3536).

### Description and Comparisons

#### Skull: general comments

Unfortunately there are no complete or nearly-complete skulls of this species. As such, few bones are known, but the rostrum, braincase, and mandible are present (Figs. 9, 10, 11, 12, 13, 14, 15, 16, 17, 18, 19, 20, 21, 22,23).

The surface ornamentation is composed of elliptical pits (Figs. 9, 10,11), rather than the subpolygonal pits and deep grooves usually seen in neosuchians, peirosaurids and *Araripesuchus* [93]–[95], or the irregular pattern of shallow sulci found in most notosuchians [96], [97]. The pits found in *Plesiosuchus manselii* are similar to those observed in *Geosaurus giganteus*: faintly indistinct, loosely packed and much shallower than those observed on extant crocodylians. Elliptical pits can be identified on the premaxilla of *P. manselii* (Fig. 11), but due to the poor preservation we cannot evaluate the extent of the development of this ornamentation in other elements. However, the pitted pattern is absent from the nasal, as in several metriorhynchids (e.g. *Geosaurus giganteus* [10]). This is in contrast to the ornamented pattern observed in *D. maximus*, in which many elements have large elliptical pits, and the maxillae also had elongate grooves and ridges (compare Fig. 11 to Figs. 2, 7).

#### Frontal, prefrontal, lacrimal and jugal

Due to the poor state of preservation in NHMUK PV OR40103 we cannot differentiate these elements from the nasals and maxillae (Figs. 9, 11). It is highly likely that parts of these elements are preserved in the holotype, but the sutures cannot be determined. Near to the orbital region there are numerous cracks and breaks, repaired with different adhesives and fillers at various times in the history of the specimen, and this region of the skull has the poorest state of preservation (Fig. 11). This means we cannot differentiate cracks in the fossil from genuine sutures in the region were we expect the frontals, prefrontals, lacrimals and jugals to articulate with the nasals and maxillae. The only sutures which can be verifiably identified on the dorsal surface of the snout are the premaxillary-maxilla suture and the maxillary-nasal suture. The left prefrontal in NHMUK PV OR40103 is better preserved than the right (Figs. 9, 10). Although its sutures to other elements cannot be determined, it does have the characteristic metriorhynchid enlarged, expanded dorsal surface which laterally overhangs the orbit.

#### Premaxilla and external nares

The premaxilla bears three alveoli per ramus (Fig. 12). The ornamentation on the external surface of premaxilla is composed of faint, indistinct elliptical pits, and the bone is slightly convex (NHMUK PV OR40103; Fig. 11). The premaxillae completely enclose the external nares. Along the posterior margin of the premaxilla, the posterodorsal process contacts the anterior margin of the maxilla. The suture is hard to distinguish, but it seems likely that *P*. *manselii* too shared the broad ‘U’-shape of *D. maximus* and *D. andiniensis* in dorsal view. *Contra* Hulke [24] and Owen [52] there is no premaxilla-nasal contact (as previously noted by Fraas [4]). Instead, the intervening maxilla prevents this contact, as is normal for most thalattosuchians (the only known exceptions are *Cricosaurus macrospondylus* [84] and two specimens of “*Metriorhynchus*” *brachyrhynchus* NHMUK PV R3700 and NHMUK PV R4763). In palatal view the premaxillary shelves suture along the midline (Fig. 12). *Plesiosuchus manselii* shares the single anterodorsally orientated naris morphology observed in most metriorhynchids (including *D. maximus*).

#### Maxilla

The maxillae of *Plesiosuchus manselii* are long, low and narrow (rostrum height: length of ∼0.13) (Fig. 11), lacking the deeper proportions observed [19] in *D. maximus* (rostrum height: length of 0.15) and *D. andiniensis* (rostrum height: length of 0.36). The maxillae bear 14 alveoli (NHMUK PV OR40103). Like the premaxillae, the external surfaces of the maxillae are slightly convex. The maxillary ornamentation is noticeably different from that observed in *Dakosaurus maximus*. Here the lateral and dorsal surfaces of the maxilla is covered in grooves and raised ridges orientated to the long axis of the skull. Both the grooves and ridges are shallower than those of *D. maximus*. No elliptical pits were observed on the maxillae. As with *Geosaurus giganteus* (NHMUK PV R1229, NHMUK PV OR27020), there are few neurovascular foramina, positioned dorsally to the alveolar margin (Fig. 11).

Along the dorsal midline the left and right maxillae contact each other across a long suture, which terminates posteriorly at the anterior margin of the nasal. The maxillonasal suture begins at the skull midline and forms an anteriorly pointed ‘V’-shape. With the jugals and lacrimals poorly preserved, the nature of their contact with the maxilla cannot be determined. Similarly, the contribution the maxilla made to the preorbital fossa is unknown. In palatal view, the maxillae suture along the midline to form part of the secondary palate (Fig. 12). Posteriorly and posterolaterally the maxillae contact the palatines. In addition the maxillae form the lateral border of the suborbital fenestrae.

#### Nasals

The nasals are large paired, unfused elements (Figs. 9, 10, 11). In dorsal view they are subtriangular in shape and broad. Along the midline the dorsal surfaces of the nasals are deeply trenched, with a steep longitudinal depression. The anterior margin forms an acute angle along its border with the maxilla. The external surfaces of the nasals are well ornamented, with a grooved pattern.

The nasal dorsoposterior processes cannot be distinguished from the frontal or prefrontals. The lateroposterior process would have curved ventrally to the lateral expansion of the prefrontal, contacting the lacrimal and descending process of the prefrontal. This process would have contributed to the preorbital fossa. However, once again the sutures are indistinct.

##### Squamosals

The left squamosal of the holotype (NHMUK PV OR40103; Fig. 13) is the best preserved squamosal of any known specimen of P. manselii; conversely, both squamosals are present but are incompletely preserved in NHMUK PV R1089 (Figs. 14, 15). The squamosals form the posterolateral border of the supratemporal fossae and the posterior half of the supratemporal arches (i.e. the bar that separates the supratemporal fenestra from the infratemporal fenestra; Figs. 13, 14). Only the left squamosal of NHMUK PV OR40103 preserves the anterior process (Fig. 13). Unfortunately, the squamosal-postorbital suture could not be determined. Along the posteromedial edge (medial process), the squamosal contacts the parietal. Again, the squamosal-parietal suture cannot be determined. The squamosal-parietal bar borders the posterior margin of the supratemporal fossae. The medial process is orientated slightly posterolaterally, and is narrowly exposed on the occipital surface of the skull. The medial and lateral processes of the squamosal meet to form the posterolateral corner of the supratemporal fossa.

#### Postorbital

Only the left postorbital of NHMUK PV OR40103 is preserved (Fig. 9). Unfortunately it is incomplete. The frontal process and the postorbital bar ( =  descending process) are not preserved, while the squamosal process cannot be distinguished from the squamosal itself (i.e. their suture is not clear). The squamosal process of the postorbital forms the anterior part of the supratemporal arch.

#### Parietal

The parietal forms the posterior and medial margin of the supratemporal fenestrae and fossae in dorsal view, and together with the frontal constitutes the intertemporal bar ( = frontoparietal bar) that separates the right and left supratemporal fossae on the dorsal skull midline (Figs. 9, 13, 14). The bar is only completely preserved in NHMUK PV R1089 (Fig. 14); however the suture between the frontal and parietal cannot be determined. The parietal has two lateral processes that contact the squamosals, but the sutures between these bones are difficult to observe. In occipital view, the ventral margins of the parietals contact the supraoccipital. In lateral view (within the supratemporal fenestra), the parietal overlays both the laterosphenoids and the proötics.

#### Quadrate

In all other thalattosuchians (e.g. Steneosaurus leedsi NHMUK PV R3320, Steneosaurus edwardsi NHMUK PV R3701, “Steneosaurus” obtusidens NHMUK PV R3169, Metriorhynchus superciliosus NHMUK PV R2030, Gracilineustes leedsi NHMUK PV R3540, “Metriorhynchus” brachyrhynchus NHMUK PV R3804; Dakosaurus maximus CAMSM J29419) [5], [42] the distal articular surface of the quadrate has medial and lateral convex condyles that are separated by a shallow sulcus which is directed ventromedially. When seen in distal (ventral) view the two protuberances can be clearly distinguished. In Geosaurus grandis (BSPG AS-VI-1) the sulcus is more strongly concave and considerably wider, and therefore the two condyles are particularly discrete. However, when NHMUK PV OR40103 and NHMUK PV R1089 are observed in distal view the posterior margin of the quadrate distal articular region is a continuous convex curve with no evident lateral or medial protuberance (Figs. 13B, 14B, 15). On both specimens (NHMUK PV OR40103 and NHMUK PV R1089) the sulcus is only a very shallow depression, and it does not clearly separate the distal head into two distinct condyles.

On the quadrate ventral surface there is a notable crest (‘crest B’) (Fig. 14B, 15). Compared to other thalattosuchians, the crest of NHMUK PV R1089 is not as well-defined and prominent [67], [98].

#### Laterosphenoid and Proötic

The laterosphenoid and proötic are preserved within the supratemporal fenestrae of NHMUK PV R1089. Unfortunately, the sutures between these elements and the parietal and quadrates are difficult to determine. Following Fernández *et al*. [67] the laterosphenoid would have formed the anterior margin of the trigeminal fenestra, the quadrate the ventral and posterior margin, and the proötic forming part of the dorsal margin. The trigeminal fossa is developed mainly posterior to the trigeminal foramen (a metriorhynchid apomorphy [67]).

#### Supraoccipital

The supraoccipital is exposed on the occipital surface of the skull and its external surface is slightly concave (Figs. 13, 14, 16). The supraoccipital contacts the parietal dorsally and the exoccipital laterally. The supraoccipital participates in the dorsal margin of the foramen magnum in NHMUK PV OR40103 (Fig. 13), much like “*Metriorhynchus*” *westermanni* [67], [99] and *Dakosaurus andiniensis*. Although both Gasparini *et al*. [19] and Pol & Gasparini [20] figure the supraoccipital as not participating in the foramen magnum margin in *D*. *andiniensis*; the supraoccipital of this taxon (Figure 9A of Pol & Gasparini [20]) has a very similar morphology to NHMUK PV OR40103, and the unmarked line ventral to their provisional supraoccipital border in the interpretative drawing of (Pol & Gasparini [20]: Fig. 9B), is what we interpret as being the supraoccipital suture. The supraoccipital does not contribute to the foramen magnum in “*Metriorhynchus*” *brachyrhynchus* (NHMUK PV R2618) “*Metriorhynchus*” cf. *durobrivensis* [64], *Metriorhynchus superciliosus* (AMNH FR997; [64]), *Metriorhynchus* cf. *palpebrosus* [64] and *Cricosaurus schroederi* [100]. In NHMUK PV R1089 the supraoccipital is missing, but the depression for this bone clearly shows it would have participated in the dorsal margin of the foramen magnum.

#### Exoccipital

The right portion of the exoccipital is not preserved in the holotype and has been reconstructed with plaster (Fig. 13). Medially, the exoccipital contacts the supraoccipital and dorsally/laterodorsally, the squamosals. Ventrally/ventromedially, the exoccipital would have contacted the basioccipital. However, due to poor preservation and/or fusion, these sutures are unclear. The exoccipital forms a large portion of the occipital surface of the skull. Dorsal to the paroccipital processes, the posterior surface is slightly convex. The left (and only preserved) paroccipital process of the holotype is poorly preserved. In NHMUK PV R1089 the paroccipital processes are large and pronounced (Figs. 14, 16). Although in both NHMUK PV OR40103 and NHMUK PV R1089 the paroccipital processes are incomplete and poorly preserved, it is clear that they are orientated dorsally (Figs. 13, 14, 16). The exoccipital forms the lateral and ventral margins of the foramen magnum. Ventrolateral to the occipital condyle there are two large foramina (for the internal carotid arteries).

#### Occipital condyle

As with other metriorhynchids [5], [64], the occipital condyle of NHMUK PV R1089 is largely formed by the basioccipital, while the exoccipitals contribute to the dorsal margin (Fig. 16). In the Oxford Clay metriorhynchids (*Metriorhynchus superciliosus* NHMUK PV R6859, NHMUK PV R6860; *Gracilineustes leedsi* NHMUK PV R3014, NHMUK PV R3015; “*Metriorhynchus*” *brachyrhynchus* NHMUK PV R3804) the exoccipital contributes only to the lateral sections of the dorsal margin, leaving a gap between them formed solely by the basioccipital. However, in NHMUK PV R1089 the exoccipital covers the entire dorsal margin of the occipital condyle.

#### Basioccipital

The basioccipital forms the ventromedial part of the occipital region of the skull (Figs. 13, 14, 15, 16). The sutures between the basioccipital and exoccipital are largely unclear. Two processes project ventrolaterally, forming the basal tubera. Medially, between the basal tubera, there is a deep fossa. Within this fossa is the Eustachian foramen ( =  part of the median pharyngeal system) [101].

#### Palatine

**Figure 24:**
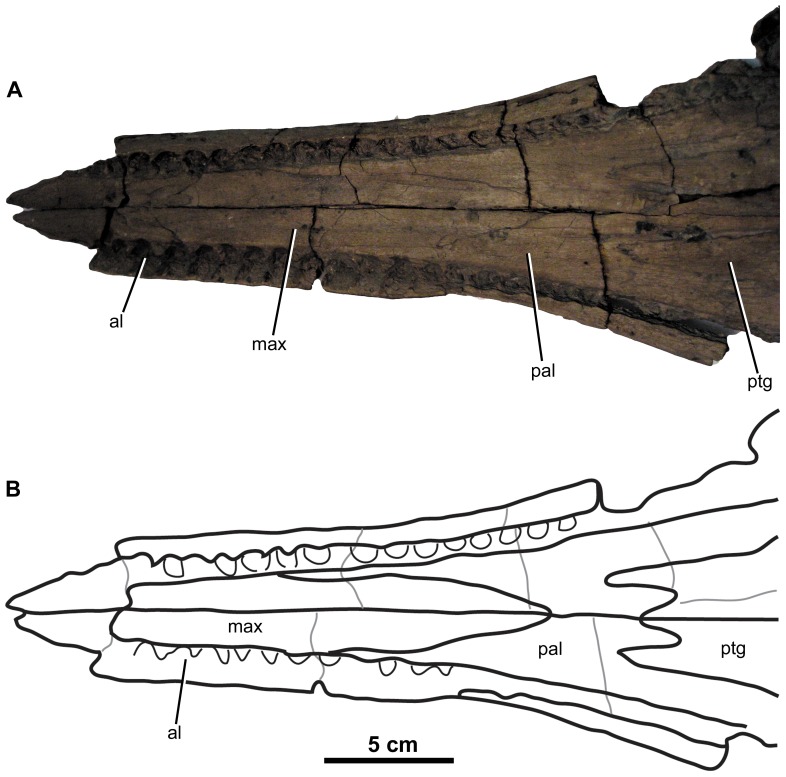
*Gracilineustes leedsi*, holotype NHMUK PV R3540. Snout in ventral (palatal) view, (A) photograph and (B) line drawing (thin grey lines represent breaks). Abbreviations: al, alveolus; max, maxilla; pal, palatine; ptg, pterygoid.

The palatines are exposed on the palatal surface of the skull where they are sutured along the skull midline, much like the palatal shelves of the premaxillae and maxillae (Fig. 12). Together these three pairs of bones form the secondary palate. Anteriorly and anterolaterally the palatines met the palatal branches of the maxillae. In palatal view, the maxillopalatine suture of NHMUK PV OR40103 has a distinct ‘V’-shape, proceeding anteriorly from the suborbital fenestrae to the skull midline (Fig. 12A). This differs from that observed in other geosaurines, as in those species the maxillopalatine suture is approximately parallel to the maxillary tooth row from the suborbital fenestra until both lateral margins are united by a gentle convex curve, e.g. “*Dakosaurus*” sp. in Buchy *et al*. [27]; “*Metriorhynchus*” *brachyrhynchus* (see text-Fig. 58 in Andrews [5]; NHMUK PV R3804, NHMUK PV R3700); in *Torvoneustes carpenteri* BRSMG Cd7203 the palatine is missing, but the suture on the maxilla for the palatine is preserved, and it too follows this usual morphology [18], [47]). In metriorhynchines the maxillopalatine sutural contact is very different in form; these taxa have two non-midline anterior processes (i.e. one on either side of the midline; Fig. 24). In *Metriorhynchus superciliosus* (e.g. GLAHM V1009, SMNS 10115, SMNS 10116) the two anterior processes are separate from both the mid-line and the maxillary alveolar border; however in *Gracilineustes leedsi* (NHMUK PV R3540) the lateral margins of these process merge with the maxillary alveolar border (Fig. 24).

The single midline anterior process of the palatine observed in geosaurines appears to be the basal condition within Thalattosuchia, as teleosaurids [5] and basal metriorhynchoids such as *Teleidosaurus calvadosii* (NHMUK PV R2681) [58] share this morphology. As such, the presence of two non-midline anterior processes in Metriorhynchinae is an autapomorphy of that clade.

In addition to having a unique shape of maxillopalatine suture within Metriorhynchidae, in NHMUK PV OR40103 the anterior extent of the palatine is also unique. In this specimen, the palatine extends anteriorly along the midline so that it is level to the fifth maxillary alveolus. In no other known metriorhynchid does the palatine extend that far anteriorly (Table 1). Furthermore, the shape of the palatine is unique in NHMUK PV OR40103. In all other taxa where the three-dimensional shape of the palatine is preserved, the palatal surface is gently convex (e.g. “*Dakosaurus*” sp. in Buchy *et al*. [27]; “*Metriorhynchus*” *brachyrhynchus* NHMUK PV R3804; *Cricosaurus araucanensis* in Gasparini & Dellapé [65]; *Metriorhynchus superciliosus* GLAHM V1009). However, in NHMUK PV OR40103 the palatal surface of the palatine is strongly convex (Figs. 11, 12), with a pronounced ridge along the skull midline (i.e. where the palatines suture). We must note however, that the natural shape of the palatines is rarely preserved in metriorhynchids (most likely caused by dorsoventral compression of the skull distorting these elements). As such, the variability of palatine convexity is currently unknown.

#### Fenestrae and fossae

The exits for the post-temporal openings on the occipital surface of the skull cannot be determined for either NHMUK PV OR40103 or NHMUK PV R1089, due to poor preservation. However, the exits for the post-temporal openings on the posterior wall of the supratemporal fenestra are very large in NHMUK PV R1089, as they are: 1) larger than the fenestra and fossa for the trigeminal nerve, and 2) wider in mediolateral width than the foramen magnum. Interestingly, the post-temporal opening in other metriorhynchids is either reduced or absent [67], [102]. Fernández *et al*. ([67]:373) state that the: “Obliteration or reduction of the post-temporal foramen can be variable within species, such as *Cricosaurus araucanensis* (MLP 72-IV-7-1; 71-IV-7-2; 71-IV-7-4)”. However, in no other known thalattosuchian species are the post-temporal openings wider than, or as wide as, the foramen magnum.

Other fenestrae are not preserved. As stated above, most thalattosuchians either lack or have a highly reduced naso-oral fenestra, and metriorhynchids lack external mandibular fenestrae. Neither the preorbital fossae, infratemporal fenestrae nor the lacrimal-prefrontal fossae are preserved.

#### Mandible: general comments

The mandibles of NHMUK PV OR40103a and NHMUK PV R1089 are better preserved than the mandibles of *D. maximus* (Figs. 17, 18, 19, 20, 21). The mandibles of NHMUK PV OR40103a have become separated, with the right ramus better preserved (Figs. 17, 18). The mandible of NHMUK PV R1089 has thirteen alveoli (*contra* Owen [54] and Lydekker [66] who stated 14; Lydekker [3] originally considered there to be 13 alveoli, but later [66] considered the anterior end to be missing and with it two alveoli), with nine teeth adjacent to the mandibular symphysis (Fig. 21). It has been subjected to post-mortem dorsoventral compression, with the dentary tooth row flattened such that it is no longer its natural shape (with the three anterior-most alveoli suffering the worse of the compression). The posterior halves of both rami are broken off (consisting of the articular, prearticular, and most of the surangular and angular; see Figs. 19, 20). Both specimens preserved the surangulodentary groove on the lateral surface of dentary and surangular. The groove is deeply excavated, and there is no evidence of a large foramen at the dentary terminus (unlike *D. maximus* and *D. andiniensis*). No external mandibular fenestra is evident.

#### Dentary

In NHMUK PV OR40103a the sutures between the dentary and the surangular and angular are difficult to determine, because disarticulated ribs lie over the region where these bones meet, and because of the poor preservation of the posterior end of the dentary (Figs. 17, 18). However, in NHMUK PV R1089 the sutures between the dentary and both the surangular and angular are easily identified (Figs. 19, 20). The lateral surfaces of the dentaries are gently convex in NHMUK PV OR40103a, while in NHMUK PV R1089 the dorsoventral compression has resulted in the dentaries losing their natural shape. The lateral surface of the dentary is pitted, especially at the anterior end, although not as strongly as *D. maximus*. The dorsal margin of the dentary is straight. The ventral margin is also straight, except for its anteriormost part where the margin gently curves anterodorsally. The dentary alveoli are very large, closely set together and almost circular (NHMUK PV R1089; Fig. 21).

Along the midline, the dentaries contact to form most of the mandibular symphysis (Figs. 17, 21). The splenials contact the dentaries across a length of approximately 60% of the symphyseal midline. They form a wide ‘V’-shaped suture pointed anteriorly in dorsal view. The dentaries continue to contact the splenial ventrally. This suture rises dorsally on the medial surface of the mandible, coming close to the dentary tooth-row, until the coronoid overlies the contact between both elements.

The dentary interalveolar spaces of NHMUK PV R1089 are all very small, being less than a quarter of the length of the immediate alveoli and typically far shorter (Fig. 21). In other genera within Geosaurini this pattern of extreme reduction in interalveolar spaces is also present (*Dakosaurus maximus* Figs. 2, 3; *Torvoneustes carpenteri* [18]), in particular in the region of the symphyseal dentary alveoli. This pattern is also observed in the holotype of the Middle Jurassic geosaurine metriorhynchid *Suchodus durobrivensis* [66]. The enlargement of alveolar diameter, coupled with a reduced alveoli count, in *Suchodus durobrivensis* (NHMUK PV R1994) and Geosaurini results in the loss of the thalattosuchian dentary ‘diastema’ (the large distance between dentary alveoli 4 and 5). What is curious is that this ‘diastema’ is still present in other thalattosuchian clades that have reduced tooth-rows, such as in the brevirostrine teleosaurid *Machimosaurus mosae* [103]. Furthermore, the longirostrine polydont (30+ alveoli) metriorhynchine metriorhynchid *Gracilineustes leedsi* (NHMUK PV R2042) has very small dentary interalveolar spaces but the ‘diastema’ is still present.

#### Splenials

The splenials suture together along the mandible midline to form part of the mandibular symphysis (Figs. 17, 21). In medial view, the splenials cover most of the surface ventral to the coronoids (NHMUK PV R1089; Fig. 21). The medial surface of the splenial can be seen on the right ramus of NHMUK PV OR40103a (Fig. 17). As with other metriorhynchids [5] the splenial begins to thin posteriorly, terminating approximately level to the coronoid process. The dentary-splenial suture rises dorsally on the medial surface of the mandible, coming close to the dentary tooth-row, until the coronoid overlies the contact between both elements.

#### Angular and surangular

The angular and surangular are strongly sutured along their entire border, with the angular forming the ventral half of the posterior mandible and the surangular forming the dorsal half (NHMUK PV OR40103a, NHMUK PV R1089; Figs. 17, 18, 19, 20). The angular ventral margin is gently concave, curving dorsally towards the jaw joint. The angular terminates significantly higher than the dentary tooth row. As with the angular the surangular gently curves dorsally, and possesses a well-developed coronoid process.

#### Coronoids

Only the left coronoid of NHMUK PV R0189 is preserved (Fig. 21). It is a thin, elongate bone that overlies the surangular, dentary and splenial along the dorsomedial surface of the mandible. Posteriorly it forms the medial surface of the coronoid process, and then it proceeds anteriorly along the dorsomedial surface overlying the surangular and splenial. It continues anteriorly until it is level with the last dentary alveolus.

#### Prearticulars

Both of the prearticulars are present on NHMUK PV R1089 (Figs. 19, 20). The prearticular is a small bone, exposed on the medial surface of the mandible. They are bound laterally and ventrally by the surangular and angular, and dorsally by the articular. The prearticulars are posterior to both the splenials and coronoids, but do not contact either. They are orientated posterodorsally in medial view.

#### Articulars

The articulars are preserved in both NHMUK PV OR40103a (Fig. 18) and NHMUK PV R1089 (Figs. 19, 20), although their morphology cannot be viewed in the former. The articular is exposed dorsally and medially, forming the posterior-most portion of the mandible, including the mandibular component of the jaw joint and the retroarticular process. Medially the articular contacts the angular and prearticular, anteriorly it contacts the surangular, posteriorly it contacts the angular, and laterally both the surangular and angular. The articular surface for the reception of the quadrate (glenoid fossa) is very different from that of other metriorhynchids. In basal metriorhynchids from the Oxford Clay Formation (e.g. *Metriorhynchus superciliosus*) the articular surface has two shallow concavities separated by a low oblique ridge [5]. This ridge-and-cavity morphology corresponds to the sulcus-and-condyle morphology of the quadrate condyles in these taxa. In NHMUK PV R1089 (with the right articular being better preserved) no ridge is visible, nor are there two concavities. There is instead a single, deep concavity orientated slightly anteromedially. This matches the modified quadrate distal articular surface. Separating the glenoid fossa and the dorsal surface of the retroarticular process is a high raised ridge that is orientated medially. The dorsal surface of the retroarticular process is concave and triangular in shape. The medially margin is almost straight, but is orientated slightly posterolaterally. The lateral margin is strongly orientated posteromedially.

#### Dentition: tooth morphology

The dentition of *Plesiosuchus manselii* is almost identical to that of *D. maximus*. Each tooth shows a caniniform morphology, as they are single cusped and mediolaterally compressed (Figs. 17, 18, 22, 23). No constriction is present at the crown/root junction, but the boundary is evident through colour and texture. The basal sections are wider mediolaterally, creating a more circular to slightly ovoid cross-section. The teeth lack the distinctive apicobasal faceting observed on the labial surface of contemporaneous *Geosaurus* species [10], [11]. The crowns are robust and large, and cingula and accessory cusps are absent. Based on the holotype and NHMUK PV R1089, this species has a dental formula per ramus of: three premaxillary, 14 maxillary and 13 dentary teeth.

#### Dentition: ornamentation, carinae and wear

The enamel surface ornamentation is composed of numerous apicobasally aligned ridges, which are fairly well-packed but are of low-relief (Figs. 18, 22, 23). This makes them difficult to properly observe without either optical aids, or good lighting. The ornamentation differs considerably from the densely packed and high ridges observed in *Torvoneustes* and *Metriorhynchus* [2], [11], and the light, anastomosed pattern observed in *D. maximus* and *Geosaurus giganteus* [11].

The teeth have carinae comprised of both denticles and a keel, as in true ziphodont teeth. The carinae are well-defined, extending from the base to apex of the crown on both the mesial and distal margins. Denticles run the entire length of the preserved carinae. Due to incomplete preservation of the enamel, individual denticles of NHMUK PV OR40103 are hard to observe. While the teeth of *P. manselii* holotype have poorly preserved enamel, the denticles are rectangular in shape (Fig. 23). All other metriorhynchid species with denticulated teeth have denticles that are rounded in lingual view [2], [11], [20]. This rectangular morphology is also observed in the referred *P. manselii* specimens in the Museum of Jurassic Marine Life. An isolated crown from the Late Jurassic of Spain [28] (described below) that shares the same enamel ornamentation pattern of the *P. manselii* holotype (apicobasal ridges, well-packed but are of low-relief) has microziphodont and rectangular denticles which are substantially smaller than those of *Dakosaurus maximus* and *D. andiniensis* [11], [20]. Denticle density is also noticeably higher.

Spalling of the enamel and the characteristic macroscopic wear observed on the crowns of *D. maximus* are unknown in *P. manselii*. However, this is could be due to the latter having far fewer well-preserved crowns. Two further specimens in the Museum of Jurassic Marine Life are referable to *Plesiosuchus manselii*. These too lack macroscopic wear and enamel spalling, as does the isolated Spanish crown [28].

cf. Plesiosuchus manselii.

v 2010 *Dakosaurus* sp. – Ruiz-Omeñaca *et al*., p.193, (Fig. 1, [28]).

#### Specimen

MUJA-1004– isolated tooth.

#### Locality and horizon

La Griega Beach, Asturias, Northern Spain. Tereñes Formation, Kimmeridgian, Upper Jurassic.

#### Description

**Figure 25:**
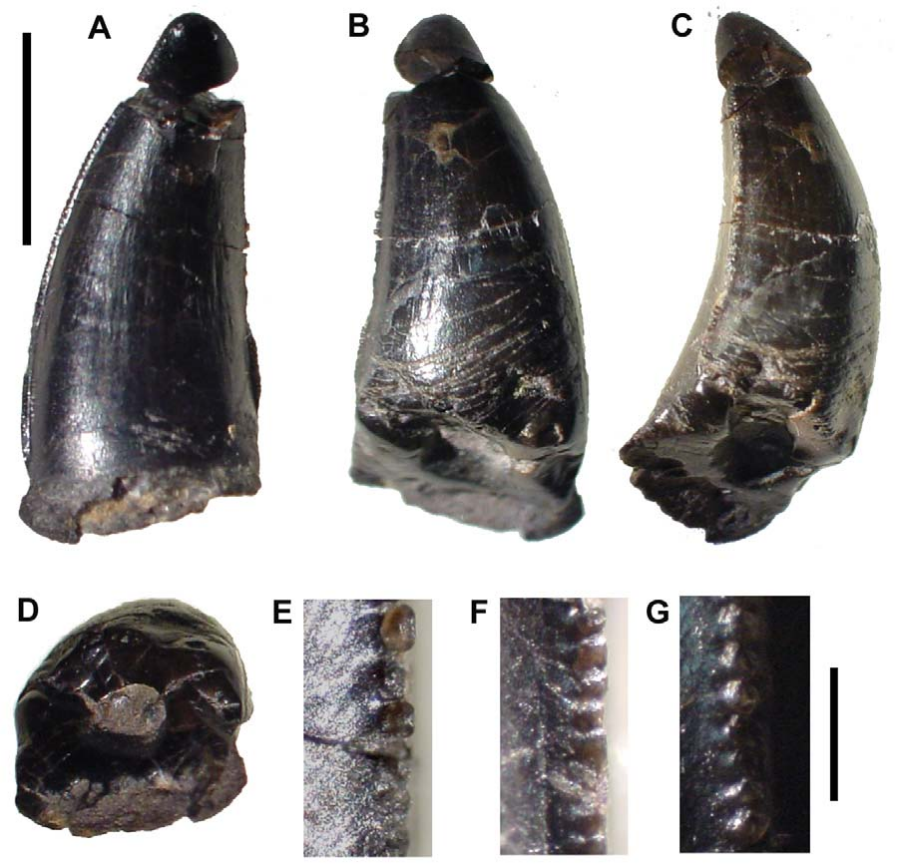
cf. *Plesiosuchus manselii*, MUJA-1004. Isolated tooth in: (A) lingual view, (B) labial view, (C) distal view, and (D) basal view. Close-up on the denticulated carinae, showing: (E) middle of the distal carina, (F) base of the distal carina, and (G) middle of the mesial carina. Scale bar: 5 mm (A–D) and 0.5 mm (E–G). Figure modified from Figure 1 in Ruiz-Omeñaca *et al*. [28].

The tooth MUJA-1004 is an isolated tooth crown that lacks the root (Fig. 25). The apex was damaged during excavation, and is broken (Fig. 25). The crown itself is relatively small, being 10.3 mm in apicobasal length, basal mesiodistal width is 4.9 mm, and the basal labiolingual width is 4.2 mm. The tooth is single cusped with slight mediolateral compression, and curved lingually. The crown also curves distally, with one edge being convex and the other straight (Fig. 25A, 25B); thereby allowing their identification as the mesial and distal edges, respectively. The crown lacks the apicobasal faceting observed on the labial surface of contemporaneous *Geosaurus* teeth [10], [11]. The base of the crown is sub-rounded (Fig. 25D), with a basal mesiodistal to labiolingual width ratio of 1.17.

In MUJA-1004, carinae are comprised of both denticles and a keel. There are only carinae on the mesial and distal edges of the tooth, with no split or supernumerary carinae, or accessory ridges. Contiguous true denticles are present along the mesial and distal borders, creating well-defined carinae. Interestingly, the denticles are larger on the distal carina than on the mesial carina, and larger toward the middle of the carina than nearer the base (Fig. 25E–F). Near the middle of the carinae, the denticle densities are: 6 denticles/mm on the distal carina and 8–10 denticles/mm on the mesial one; and near the base of the crown: 8 denticles/mm on the distal carina and 10–12 denticles/mm on the mesial carina.

Compared to other ziphodont metriorhynchids MUJA-1004 had a similar number of denticles per unit length as *Geosaurus* [11], whereas *Dakosaurus maximus* and *D. andiniensis* have considerably fewer [11], [20]. In MUJA-1004, the carinae extend from the base to apex of the crown. Denticles run the entire length of the preserved carinae (homogenous), differing from basal geosaurines such as “*Metriorhynchus*” *brachyrhynchus* and the “Mr Leeds’ specimen” which had heterogeneous carinae (carinae have numerous short rows of between 2 and 10 denticles) [2]. The well-defined denticles of MUJA-1004 (Fig. 25E–G) differ from basal geosaurines and *Torvoneustes*, as these genera have poorly defined denticles that are difficult to distinguish even under SEM [2]. Overall, the denticles have a fairly consistent height (isometric), but shape and dimensions can vary substantially (poorly isomorphic) (Fig. 25E–G). As with the holotype of *P. manselii*, the denticles are rectangular in shape (Fig. 25E–G).

The labial and lingual surfaces of MUJA-1004 are seemingly smooth when observed without optical aids; however under stereomicroscope the enamel ornamentation is composed of long apicobasally aligned ridges on both surfaces (Fig. 25A–C).

*Geosaurus* Cuvier, 1824 [50].

#### Type species

*Lacerta gigantea* von Sömmerring, 1816 [104] (following Recommendation 67B of the ICZN Code).

#### Etymology

“Gaia lizard”. *Ge-* is Ancient Greek for Gea (or Gaia), a Titan in Greek mythology that was an Earth goddess and mother to many gods. Note that Young & Andrade [10] incorrectly considered *Ge*- as referring to “the earth” (i.e. earth lizard), when in fact Cuvier ([50]:184) stated: “par allusion à la terre mère des géans”.

#### Geological range

Upper Kimmeridgian to lower Hauterivian. Young & Andrade [10] were correct in that the holotype of *Geosaurus lapparenti* is late Valanginian in age; however they were incorrect in stating there are no Hauterivian metriorhynchids. A second specimen described by Debelmas [40] is from the early Hauterivian.

#### Geographical range

France and Germany.

#### Emended diagnosis

Metriorhynchid crocodylomorph with the following unique combination of characters (autapomorphic characters are indicated by an asterisk): triangular teeth in labial/lingual view, with strong mediolateral compression*; carinae formed by a keel and true microscopic denticles (microziphodonty, dimensions do not exceed 300 µm); tooth enamel ornamentation is inconspicuous, only visible using SEM and composed of microscopic ridges arranged an anastomosed pattern; upper and lower jaw dentition arranged as opposing blades (with maxillary overbite)*; reception pits on the lateral margin of the dentary; separation between the premaxilla and the nasal less than half the midline length of the premaxilla; inflexion point on the lateral margin of the prefrontals (in dorsal view) is directed posteriorly at an angle of approximately 70 degrees from the anteroposterior axis of the skull; acute angle (close to 60 degrees) between the medial and the posterolateral processes of the frontal; lacrimal-prefrontal fossa present, with a crest along the sutural contact; large, robust sclerotic ring within the orbit, composed of 12 sclerotic ossicles*; mandibular symphysis moderately long (approximately 8 out of 13 dentary teeth are adjacent to the symphysis)*.

*Torvoneustes* Andrade *et al*., 2010 [11].

#### Type species

*Dakosaurus carpenteri* Wilkinson *et al*., 2008 [18] (following Recommendation 67B of the ICZN Code).

#### Etymology

“Savage swimmer”. *Torvus-* is Latin for savage, while *neustes* is Ancient Greek for swimmer.

#### Geological range

Kimmeridgian.

#### Geographical range

England.

#### Emended diagnosis

Metriorhynchid crocodylomorph with the following unique combination of characters (autapomorphic characters are indicated by an asterisk): robust teeth, mostly conical in shape, with little-to-moderate mediolateral compression and blunt apices; carinae formed by a keel and true microscopic denticles (microziphodonty, dimensions do not exceed 300 µm); denticles form a contiguous row along both the mesial and distal carinae, but are poorly defined, being difficult to observe even under SEM*; superficial enamel ornamentation extends onto the keel at the apical half of the crown (which in non-denticulated teeth is the false-ziphodont condition)*; tooth enamel ornamentation is intense, on the basal third/half of the crown the ornamentation is composed of apicobasally aligned ridges, which become an anastomosed pattern in the apical third/half*; inflexion point on the lateral margin of the prefrontals (in dorsal view) is directed posteriorly at an angle of approximately 70 degrees from the anteroposterior axis of the skull; acute angle (close to 60 degrees) between the medial and the posterolateral processes of the frontal.

### Phylogenetic Results

**Figure 26:**
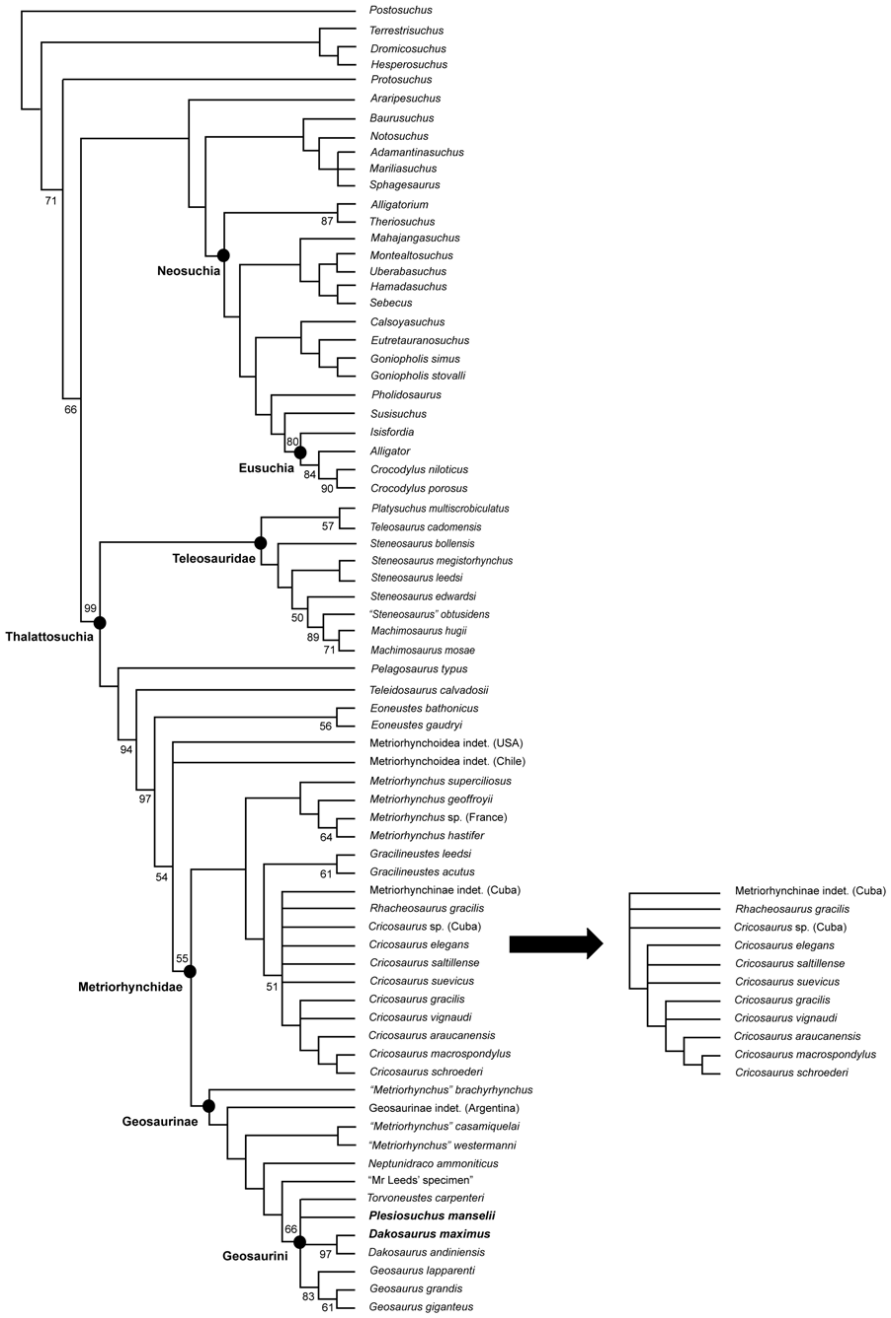
Strict consensus of 22 most parsimonious cladograms, showing the phylogenetic relationships of *Plesiosuchus manselii* and *Dakosaurus maximus* within Metriorhynchidae when all characters are unordered. Note that while both are members of the subclade Geosaurini, *P. manselii* is in an unresolved position with *Torvoneustes*, *Geosaurus* and *Dakosaurus* (*D. andiniensis* + *D. maximus*). Length = 627; ensemble consistency index, *CI* = 0. 506; ensemble retention index, *RI* = 0. 860; rescaled consistency index, *RC* = 0. 435. The black arrow points to the only consistent difference in the 50% majority rule consensus topology: the slightly greater resolution in Rhacheosaurini. Bootstrap-values are given above or below the relevant node.

From the first (unordered) phylogenetic analysis, 22 most parsimonious cladograms were recovered (Length = 627, CI = 0.506; RI = 0.860; RC = 0.435). The topology of the strict consensus of these cladograms is identical to that reported by Young *et al*. [2], except: 1) the base of Rhacheosaurini is now unresolved and 2) *Plesiosuchus manselii* is in an unresolved position within Geosaurini (Fig. 26). In other words, there is no clade (a monophyletic *Dakosaurus*) including *D*. *maximus*, *D*. *andiniensis* and *P*. *manselii* that excludes *Geosaurus* and *Torvoneustes*. This result supports our monographic re-description and our contention that *P*. *manselii* is distinct from *Dakosaurus*, and belongs to its own genus: *Plesiosuchus*.

Even with the expanded number of teleosaurid species, *Pelagosaurus typus* is still found to be the basal-most metriorhynchoid (following the result of Young *et al*. [2]). Within Teleosauridae, *Steneosaurus* is found to be paraphyletic in regards to *Machimosaurus*, with *S. edwardsi* and *S. obtusidens* as successive sister taxa to the clade *M. hugii* + *M. mosae*. This result does not support the contention that the Callovian “*Steneosaurus*” *obtusidens* is a subjective junior synonym of the Kimmeridgian taxon *Machimosaurus hugii* [103].

Very strong support was found for the clades: Thalattosuchia (bootstrap = 99%), metriorhynchoids more derived than *Pelagosaurus* (bootstrap = 94%), metriorhynchoids more derived than *Teleidosaurus* (bootstrap = 97%), *Dakosaurus maximus* + *D. andiniensis* (i.e. the genus *Dakosaurus*: bootstrap = 97%), *Crocodylus* (bootstrap = 90%), *Machimosaurus* including “*Steneosaurus*” *obtusidens* (bootstrap = 89%), Atoposauridae (bootstrap = 87%), crown-group Crocodylia (bootstrap = 84%), *Geosaurus* (bootstrap = 83%), and Eusuchia (bootstrap = 80%). As such, within Geosaurini there is strong support for a monophyletic *Geosaurus*, and a monophyletic *Dakosaurus*, but no support that *Plesiosuchus manselii* is closely related to either clade.

There is strong-to-moderate support for the clades: *Machimosaurus hugii* + *M. mosae* (bootstrap = 71%), Crocodyliformes (bootstrap = 71%), Geosaurini (bootstrap = 66%), Mesoeucrocodylia (bootstrap = 66%), *Metriorhynchus hastifer* + *Metriorhynchus* sp. (bootstrap = 64%), *Gracilineustes* (bootstrap = 61%) and *Geosaurus giganteus* + *G. grandis* (bootstrap = 61%).

The 50% majority-rule consensus topology is identical to the strict consensus topology, except there is now more resolution at the base of Rhacheosaurini, with *Cricosaurus elegans*, *C*. *saltillense* and *C. suevicus* forming a clade (and polytomy) to the exclusion of “*Cricosaurus*” sp., Metriorhynchinae indet. and *Rhacheosaurus gracilis* (Fig. 26). This clade is recovered ∼80–86% of the time, depending on the different start seed used. Sometimes a sister group relationship between *Dakosaurus* and *Geosaurus* (to the exclusion of *Plesiosuchus*) is found in the 50% majority rule consensus topology, again depending on the start seed used. However, altering the start seed generally disrupts this relationship, with Geosaurini being a polytomy of all four genera. As there is no consistent pattern, we consider the interrelationships within Geosaurini unresolved for the unordered analysis.

**Figure 27:**
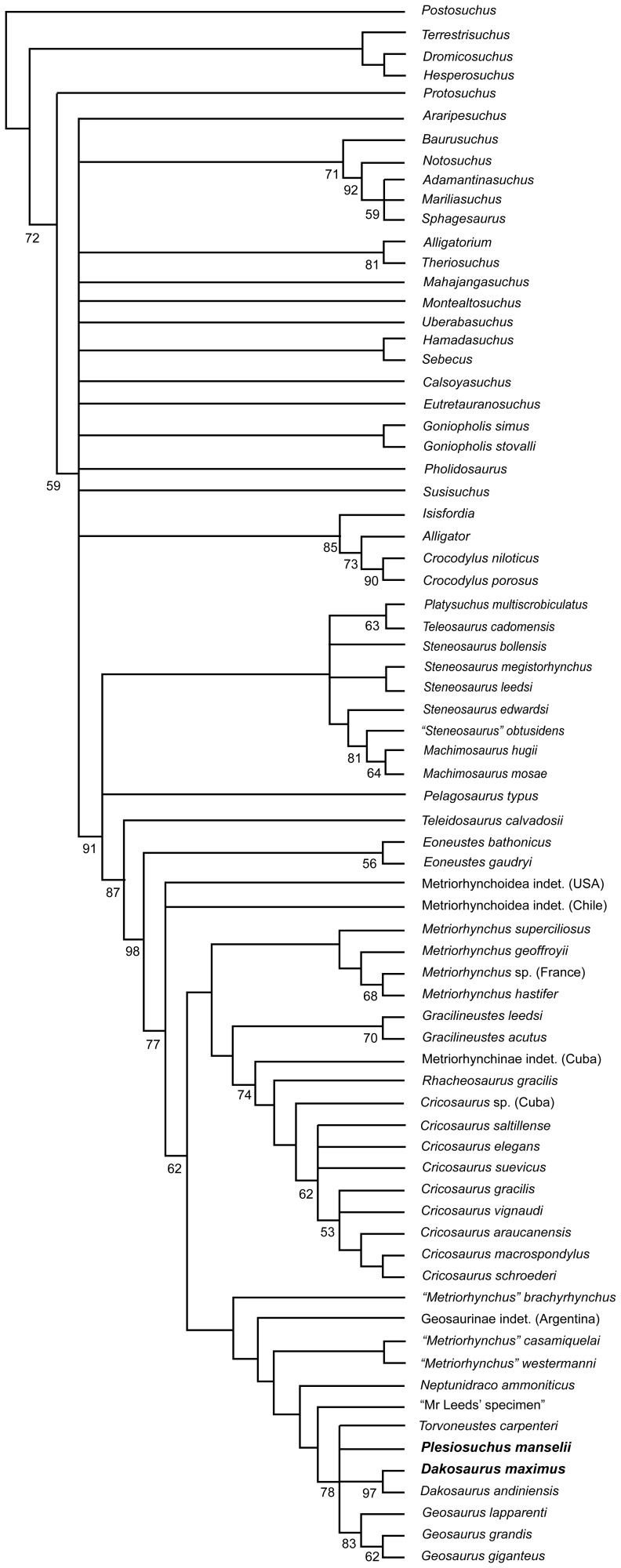
Strict consensus of 195 most parsimonious cladograms, showing the phylogenetic relationships of *Plesiosuchus manselii* and *Dakosaurus maximus* within Metriorhynchidae when 40 characters are ordered. Length = 667; ensemble consistency index, *CI* = 0. 481; ensemble retention index, *RI* = 0. 863; rescaled consistency index, *RC* = 0. 415. Bootstrap-values are given above or below the relevant node.

The second (ordered) phylogenetic analysis returned 195 most parsimonious cladograms (Length = 667, CI = 0.481, RI = 0.863, RC = 0.415). The topology of the strict consensus of these cladograms is highly unresolved (Fig. 27). Overall the relationships between non-metriorhynchid species are generally far less resolved in the ordered analysis than in the unordered analysis. However, the relationships at the base of Rhacheosaurini are now fully resolved (Fig. 27), in stark contrast to the polytomy recovered in the unordered analysis (Fig. 26).

**Figure 28:**
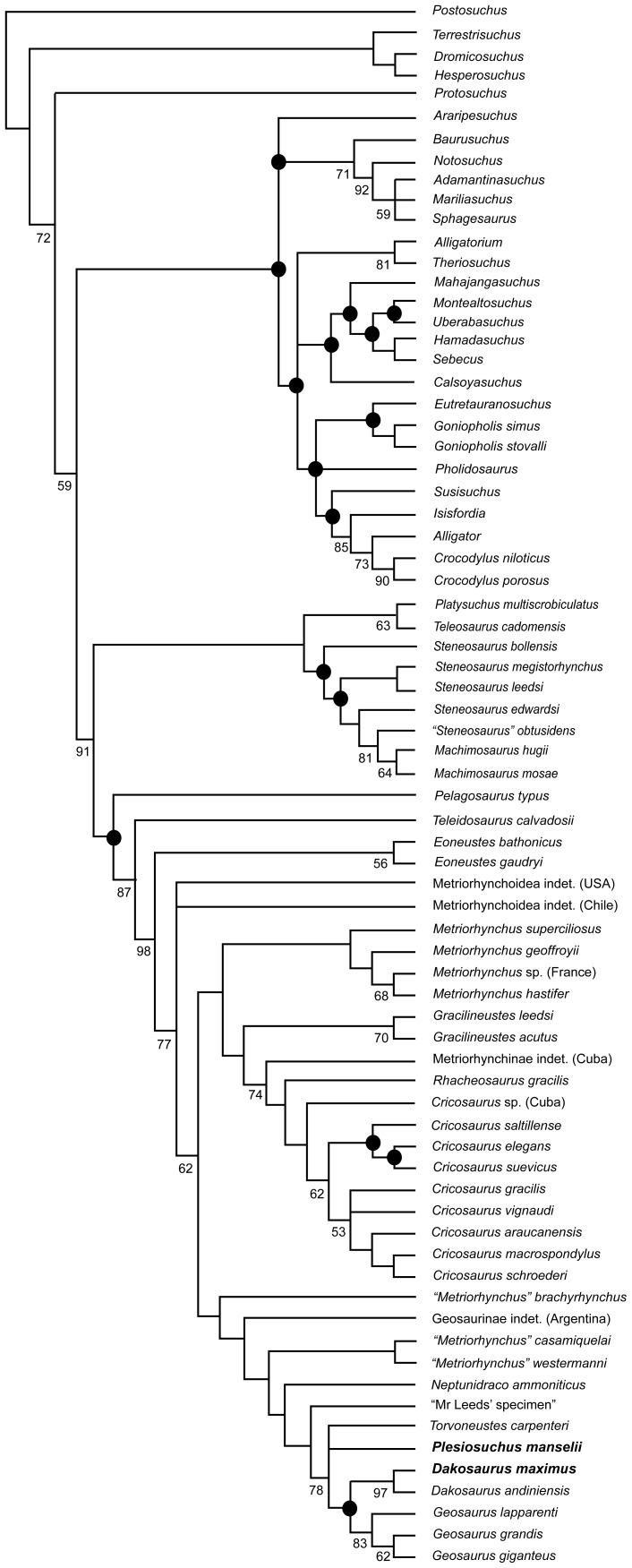
50% majority rule consensus topology of 195 most parsimonious cladograms, showing the phylogenetic relationships of *Plesiosuchus manselii* and *Dakosaurus maximus* within Metriorhynchidae when 40 characters are ordered. Length = 667; ensemble consistency index, *CI* = 0. 481; ensemble retention index, *RI* = 0. 863; rescaled consistency index, *RC* = 0. 415. The black circles at certain nodes denote clades that break in the strict consensus topology. Bootstrap-values are given above or below the relevant node.

As with the unordered analysis, *Plesiosuchus manselii* does not form a clade with *Dakosaurus maximus* and *D*. *andiniensis* (Figs. 27, 28). This result further supports our contention that *P*. *manselii* is distinct from *Dakosaurus*. Interestingly, yet again a sister group relationship between *Dakosaurus* and *Geosaurus* (to the exclusion of *Plesiosuchus*) is found in the 50% majority rule consensus topology (Fig. 28). This clade is recovered ∼73–77% of the time (depending on the start seed used). This result is in agreement with the ordered analysis in the online supplementary material of Young *et al*. [14], as they too found a sister group relationship between *Dakosaurus* and *Geosaurus* which excluded *Plesiosuchus*.

Very strong support was found for the clades: Thalattosuchia (bootstrap = 91%), metriorhynchoids more derived than *Teleidosaurus* (bootstrap = 97%), *Dakosaurus maximus* + *D. andiniensis* (i.e. the genus *Dakosaurus*: bootstrap = 97%), Notosuchidae (bootstrap = 92%), *Crocodylus* (bootstrap = 90%), Atoposauridae (bootstrap = 81%), *Machimosaurus* including “*Steneosaurus*” *obtusidens* (bootstrap = 81%), Eusuchia (bootstrap = 85%) and *Geosaurus* (bootstrap = 83%). As such, within Geosaurini there is strong support for a monophyletic *Geosaurus*, and a monophyletic *Dakosaurus*, but no strong support that *Plesiosuchus manselii* is closely related to either clade.

There is strong-to-moderate support for the clades: Geosaurini (bootstrap = 78%), metriorhynchoids more derived than *Eoneustes* (bootstrap = 77%), crown-group Crocodylia (bootstrap = 73%), Crocodyliformes (bootstrap = 72%), Notosuchia (bootstrap = 71%), *Gracilineustes* (bootstrap = 70%), *Metriorhynchus hastifer* + *Metriorhynchus* sp. (bootstrap = 68%), *Machimosaurus hugii* + *M. mosae* (bootstrap = 64%), *Platysuchus multiscrobiculatus* + *Teleosaurus cadomensis* (bootstrap = 63%), Metriorhynchidae (bootstrap = 62%), *Cricosaurus* (excluding the putative Cuban species, bootstrap = 62%) and *Geosaurus giganteus* + *Ge. grandis* (bootstrap = 62%).

**Figure 29:**
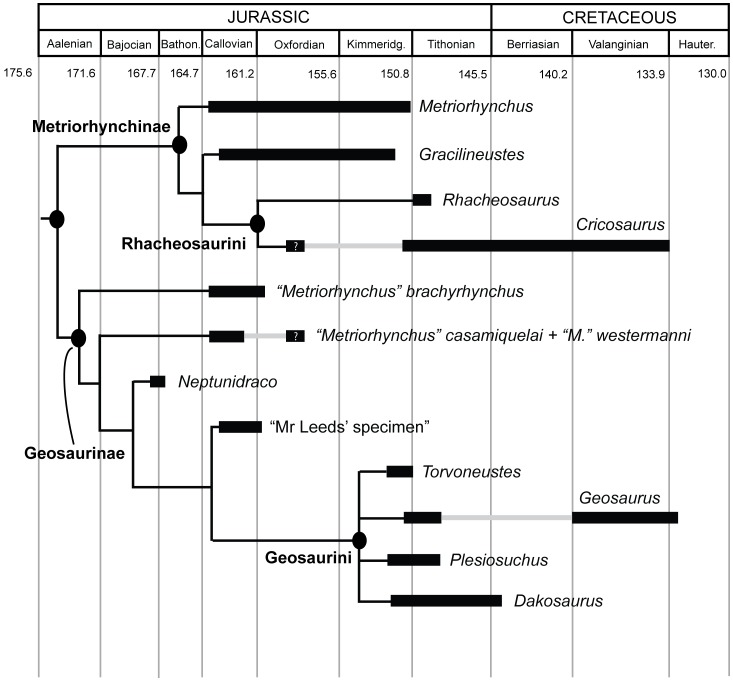
Genus-level evolutionary relationships of Metriorhynchidae, based on the phylogenetic analysis presented herein. The time-span of genera with question marks is uncertain, and the grey bars are range extensions.

**Figure 30:**
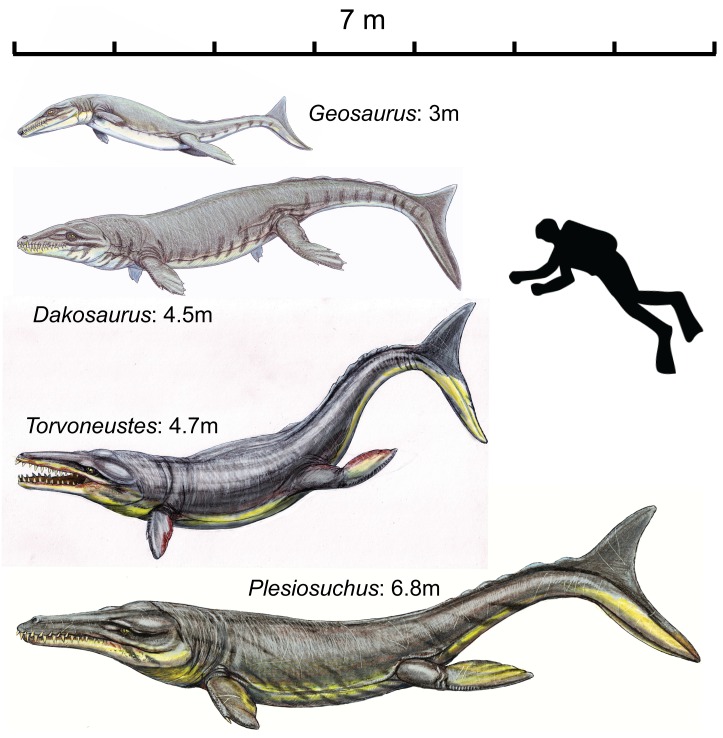
Life reconstructions showing the maximum body lengths for the four Geosaurini genera present in the late Kimmeridgian-early Tithonian of Western Europe. The species from top to bottom are: *Geosaurus giganteus*, *Dakosaurus maximus*, *Torvoneustes carpenteri* and *Plesiosuchus manselii*. The maximum known body lengths of *Torvoneustes* and *Geosaurus* are from Young *et al*. [14], while those of *Dakosaurus* and *Plesiosuchus* are from this paper. The human diver is 1.8 m in height. All metriorhynchid life reconstructions are by Dmitry Bogdanov.

Our results show that the internal relationships within Geosaurini are currently inconsistent, but there is possibly a sister group relationship between *Dakosaurus* and *Geosaurus*, with *Plesiosuchus* and *Torvoneustes* being more basal (Figs 26, 27, 28). Furthermore, the first known occurrence of all four genera is in the late Kimmeridgian of Europe (Figs 29, 30), by which time they were already morphologically distinct (Table 1). Any attempt to further elucidate the evolutionary relationships within this clade must likely await new discoveries, and a critical re-assessment of their post-cranial skeletons.

## Discussion

### The Removal of *Plesiosuchus manselii* from the Genus *Dakosaurus*

Based on our monographic revision of *Dakosaurus maximus* and *Plesiosuchus manselii* we herein remove the latter from the genus *Dakosaurus*. This decision was not solely based on our updated phylogenetic analysis, but our re-description of *D. maximus* and *P. manselii*, which identified numerous apomorphies that *D. maximus* shares with the South American *D. andiniensis* to the exclusion of all other metriorhynchids (including *P. manselii*). We found that the genus *Dakosaurus* (*D. maximus* + *D. andiniensis*) has the following eight autapomorphies that are not seen in other genera:

1. Carinae formed by a keel and true macroscopic denticles (macroziphodonty, all denticle dimensions exceed 300 µm)
2. Rostrum is dorsoventrally tall with a convex dorsal margin (oreinirostral condition)
3. Rostrum in dorsal view has a distinctly wide and blunt, “bullet” shape (amblygnathous condition)
4. Aligned set of large neurovascular foramina on the maxilla extending posteroventrally from the preorbital fossa
5. In dorsal view, the lateral margins of the prefrontals have an inflexion point directed posteriorly at an angle of approximately 50 degrees from the anteroposterior axis of the skull
6. Ventral margin of dentary sharply rises dorsally at the anterior tip
7. Very short mandibular symphysis (only one third of dentary tooth-row adjacent to the symphysis)
8. Surangulodentary groove has a well-developed foramen at the dentary terminus

The type species, *D. maximus*, can be defined by the following four autapomorphies:

1. Wear facets on the mesial and distal edges of the crown that obliterate the carinae
2. Thin lamina of bone projecting from the lateral alveolar margin of the premaxilla (“premaxillary lateral plates”)
3. Maxilla is strongly ornamented, with most of the element covered in long deep grooves and long raised ridges orientated to the long axis of the skull, but with the alveolar margin largely smooth
4. In the posterior half of the dentary, there are laminae of bone projecting from the lateral and medial dentary alveolar margins (“dentary lateral and medial plates”)

As we noted above, the preservation of *D. andiniensis* makes it difficult to assess whether it also shared autapomorphies one and two. *Plesiosuchus manselii*, however, lacks all 12 *Dakosaurus* and *D. maximus* autapomorphies. As such, the long-standing contention that *P. manselii* is a subjective junior synonym of *D. maximus* [3], [46], [53] cannot be supported. Our re-description of *P. manselii* identified six autapomorphies that this species possesses to the exclusion of all other metriorhynchids (including the two *Dakosaurus* species):

1. Tooth enamel ornamentation is largely inconspicuous, but there are apicobally aligned ridges of low-relief
2. The mesial margin of some teeth have a pronounced distal curvature
3. Palatines are strongly convex with a pronounced ridge along the midline
4. In palatal view, the palatine width narrows anteriorly from the suborbital fenestrae to the midline (a distinct elongate triangular shape)
5. The maxillopalatine suture midline terminus is level to the fourth maxillary alveolus
6. Quadrate distal articular surface is not separated into two condyles by a sulcus, and has only a very shallow depression at the centre

As such, this unique character suite precludes us from referring *P. manselii* to any other metriorhynchid genus. Moreover, *P. manselii* lacks the autapomorphies of the other two Geosaurini genera, *Geosaurus* and *Torvoneustes* and their unique character combinations (see Table 1 and revised diagnoses below). Our only remaining position is the resurrection of the genus *Plesiosuchus*. The internal relationships of Geosaurini are herein found to be unresolved, but it is possible that future studies may find that the sister taxon of *Dakosaurus* is *Geosaurus* (which we found weak support for). Nonetheless, new discoveries, especially of the post-cranial skeleton, will help elucidate geosaurin interrelationships.

### Implications for Geosaurini Systematics

Aside from supporting the removal of *P*. *manselii* from *Dakosaurus* into its own genus, the currently monospecific *Plesiosuchus*, our anatomical revisions and phylogenetic analysis also have implications for the identity and systematics of some other geosaurine specimens, in particular, the referral of *Aggiosaurus nicaeensis* to *Dakosaurus* by Young & Andrade [10]. This synonymy was based on the shared presence of unusually large dentition (apicobasal length in excess of 6 cm). In fact *Aggiosaurus nicaeensis* has the largest dentition of any known metriorhynchid (up to 12 cm in apicobasal length) [60]. However, following the removal of *P. manselii* from *Dakosaurus*, this dentition is now considered homoplastic and insufficient for assigning *D*. *nicaeensis* to *Dakosaurus* (or indeed *Plesiosuchus*). As we currently cannot consider *Aggiosaurus* to be a junior synonym of either *Dakosaurus* or *Plesiosuchus*, we must await future discoveries to determine its taxonomic affinities.

Finally, the presence of the genus *Dakosaurus* in Mexico is in question. Two recently discovered, but fragmentary, skulls from the Kimmeridgian of Mexico were referred to *Dakosaurus* based on their overall size and robustness [26], [27]. However, neither specimen is well enough preserved for us to determine with any certainty whether they belong to *Dakosaurus* or *Plesiosuchus*. Moreover, one cranial characteristic (the intratemporal flange extending anteriorly into the minimum interorbital distance) once considered an autapomorphy for *Dakosaurus* (by Young & Andrade [10]) and exhibited by one of the Mexican specimens [26], is also present in recently discovered metriorhynchine specimens from the early Tithonian of Mexico [80] (Buchy pers com., 2012). Until better preserved Mexican material is discovered, attributing these specimens to *Dakosaurus* is considered premature.

### Gape Mechanics

Metriorhynchids exhibit variation in mandibular morphology relating to the relative positions of the dentary tooth row, jaw joint and coronoid process [2]. Substantial change in mandible geometry occurs within the subfamily Geosaurinae, which is linked to the trend towards greater ‘optimum gape’ (defined as the gape at which multiple teeth come into contact with a prey item [2]). This metric was created to serve as a proxy for biomechanically optimal prey size (depth), and is derived from tooth row and mandibular morphology. Young *et al*. [2] derived the ‘optimum gape’ angle by: 1) drawing a straight line across the tips of the dentary teeth; 2) drawing a second line through the jaw joint that is parallel to the first line and 3) measuring the angle between the second line and the tip of the posterior most dentary tooth.

It is important to note that ‘optimum gape’ is not equivalent to maximum gape, which is difficult (and usually impossible) to accurately measure in fossil specimens that do not preserve soft tissues. We consider ‘optimum gape’ a conservative proxy that can be consistently measured in specimens without needing information on soft tissues, and that is biologically reasonable because, no matter the soft tissue morphology or range of gapes employed by an organism, multiple teeth must have come into contact with prey during feeding. Therefore, ‘optimum gape’ most likely represents a gape that the living animal actually employed. Furthermore, ‘optimum gape’ calculations also permit a straightforward comparison between species, by indicating the relative size of the gape (and therefore prey items that could be consumed) when the species open their jaws to an equivalent baseline (i.e. when every species opens its jaws to the point where multiple teeth would have come into contact with prey). It is important to remember, however, that maximum gapes would have almost certainly been larger than ‘optimum gapes’ in most cases, and many extant predators are even known to consume prey larger than their maximum gape through the evolution of sophisticated occlusion mechanics (such as the Great Barracuda *Sphyraena barracuda* [105]).

Basal metriorhynchines (*Metriorhynchus superciliosus*) and geosaurines (“*Metriorhynchus*” *brachyrhynchus*) have a low ‘optimum gape’, with an optimum gape angle of approximately 10–11 degrees and an optimum prey depth of 7–8% of mandibular length [2] (Table 3). These species lack the geometric changes relating to the ventral displacement of the dentary tooth row relative to the jaw joint that are seen in more derived geosaurines [2]. The geosaurine “Mr Leeds’ specimen”, the sister taxon to the highly derived subclade Geosaurini, had an optimum gape angle of approximately 15 degrees and an optimum prey depth of 13% of mandibular length, both of which are relatively greater when compared to basal members of both subfamilies [2] (Table 3). The greater gape is a result of further displacement of the dentary tooth row and an increase in tooth crown apicobasal length. Within Geosaurini ‘optimum gape’ increases further. The highly derived geosaurin *Dakosaurus andiniensis* had an optimum gape angle of approximately 23 degrees, and optimum prey depth of about 19% of the mandibular length [2] (Table 3). Although the “barracuda-mimic” *Geosaurus giganteus* (NHMUK PV OR37020) [2], had a gape comparable with that of the older “Mr Leeds’ specimen”, being approximately 16 degrees with an optimum prey depth of about 13% of the mandibular length (Table 3).

**Table 3:** Table of data derived from the optimum gape calculations.

Species	Optimum gape angle	Optimum prey depth (as % of mandibular length)	Maximum known mandible length	Maximum known optimum prey depth	Optimum prey depth with a 60 cm long mandible
*Metriorhynchus superciliosus* NHMUK PV R3016	11	8%	88 cm	7.04 cm	4.8 cm
“*Metriorhynchus*” *brachyrhynchus* NHMUK PV R3804	10	7%	82.3 cm	5.76 cm	4.2 cm
Mr Leeds’ specimen GLAHM V972	15	13%	67 cm	8.71 cm b	7.8 cm
*Geosaurus giganteus* NHMUK PV OR37020	16	13%	52 cm	6.76 cm	7.8 cm
*Dakosaurus maximus* SMNS 82043	19	15%	87.5 cm	13.13 cm	9 cm
*Dakosaurus andiniensis* Gasparini et al. [19]	23	19%	80 cm	15.2 cm	11.4 cm
*Plesiosuchus manselii* NHMUK PV R1089	24	21%	132.2 cm	27.76 cm	12.6 cm

The final column (comparing all species at a mandibular length of 60 cm) was used to directly compare the influence gape mechanics has on optimum prey depth. Note, data for *Metriorhynchus superciliosus*, “*Metriorhynchus*” *brachyrhynchus*, “Mr Leeds’ specimen” and *Dakosaurus andiniensis* are from Young *et al*. [2]. Note that the only complete mandible of “Mr Leeds’ specimen” is from a sub-adult; therefore the maximum known optimum prey depth is not from an adult. This taxon is a new genus and species; however the paper establishing these names is still in press [2].

**Figure 31:**
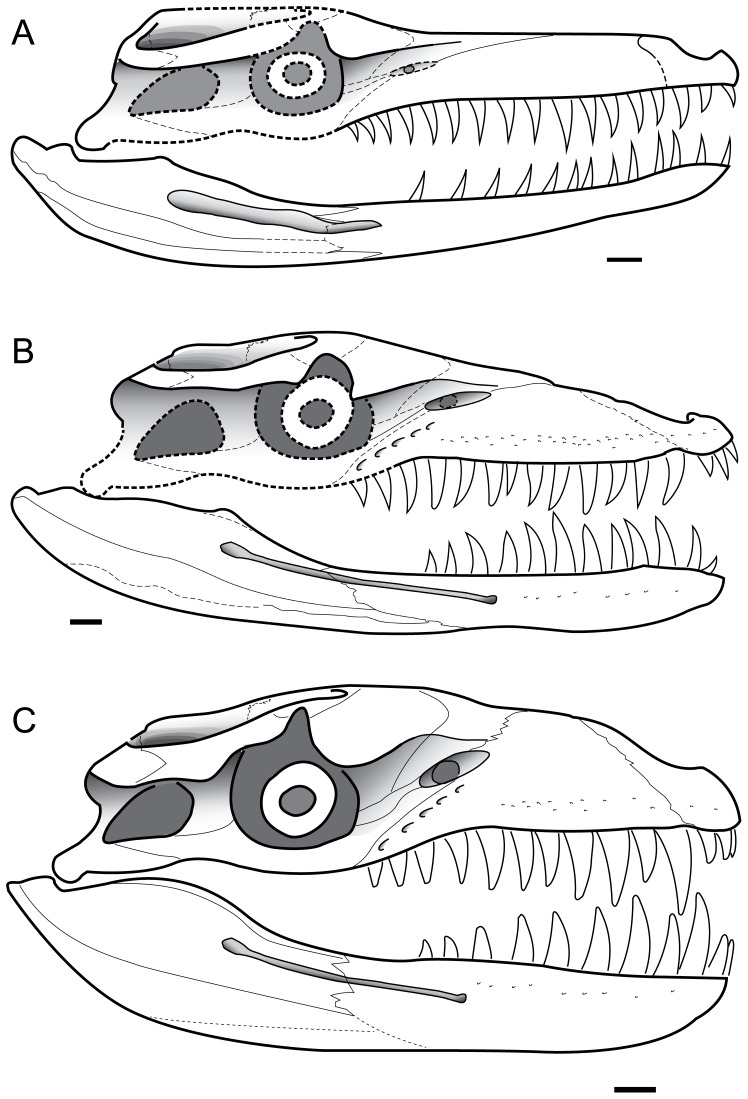
Lateral reconstructions of the skull of *Plesiosuchus manselii*, *Dakosaurus maximus* and *D. andiniensis*. Line drawings: (A) *Plesiosuchus manselii* is an original skull reconstruction based on the mandible of NHMUK PV OR40103a, while the skull is a composite of NHMUK PV OR40103 and *D. andiniensis* (due to the holotype lacking part of the orbital and infratemporal regions of the skull, these regions are shown by broken lines); (B) *Dakosaurus maximus* is an original skull reconstruction based on the mandible of SMNS 8203 and SMNS 82043, while the skull is a composite of SMNS 8203, SMNS 10819b and *D. andiniensis* (due to the neotype and referred specimen lacking the lower orbital and infratemporal regions, these regions are shown by broken lines); (C) *Dakosaurus andiniensis*, redrawn from Pol & Gasparini [20].

Based on our examinations of *Dakosaurus maximus* and *Plesiosuchus manselii* we created new cranial reconstructions, which we used to investigate their gape (Fig. 31). *Dakosaurus maximus* had an ‘optimum gape’ intermediate between “Mr Leeds’ specimen” and *D. andiniensis*, with an optimum gape angle of approximately 19 degrees, and optimum prey depth of about 15% of the mandibular length. This fits with the phylogenetic position of *D. maximus*. Interestingly, *Plesiosuchus manselii* has the greatest ‘optimum gape’ of any known metriorhynchid, with an optimum gape angle of approximately 24 degrees, and optimum prey depth of about 21% of the mandibular length (Table 3). Although the gape of *Plesiosuchus manselii* is largely comparable to that of *D. andiniensis*, there was a temporal gap between these two species of approximately 5 million years. The contemporaneous *Dakosaurus* species that lived at the same time as *P. manselii* (*D. maximus*) had a noticeably smaller ‘optimum gape’ (19 degrees vs 24 degrees respectively). This trend of gape differentiation also occurred in the late Kimmeridgian-early Tithonian of Southern Germany, where the contemporaneous geosaurins [11] also had a noticeable variation in gape (19 degrees for *D. maximus* vs 16 degrees for *Geosaurus giganteus*, see Table 3).

### Feeding Ecology

The craniodental morphologies of *Dakosaurus maximus* and *Plesiosuchus manselii* are distinct. *Dakosaurus maximus* is characterised by: a snout that is amblygnathous (wide and “bullet” shaped in dorsal view) and oreinirostral (tall with a convex dorsal margin), premaxillary ‘lateral plates’, serrated teeth with macroscopic denticles, tooth crown apices that are frequently broken or spalled, occlusal wear facets on the mesial and distal margins of the teeth, and reception pits on the dentigerous bones of both the upper and lower tooth rows (Figs. 2, 3, 4, 5, 6, 7, 8). Based primarily on these dental features, Young *et al*. [17] concluded that *D. maximus* and *D. andiniensis* were Mesozoic analogues of extant killer whales and false killer whales (adapted for increased biting performance at wide gapes, in particular exhibiting adaptations for dismembering large-bodied prey: macrophagy).

There are several additional lines of evidence, gleaned from our monographic redescription, that support this conclusion. First is the sophisticated occlusal pattern, as *Dakosaurus* (*D. maximus* and *D. andiniensis*) had tightly-packed interlocking teeth which created a precise tooth-to-tooth occlusion (see Fig. 1) [17]. Second, biomechanical modelling has shown that oreinirostral snouts (like that of *Dakosaurus*) are more resistant to both torsional and bending stresses than a platyrostral or tubular snout [106]–[108]. Third, biomechanical modelling also confirms that, in other archosaur taxa, ‘lateral plates’ like those of *Dakosaurus* occur on dentigerous bones that experience high localised stress thereby helping to dissipate feeding-induced stresses acting on the bases of adjacent teeth [85]. Fourth, *Dakosaurus* is the only known metriorhynchid to exhibit macroziphodonty [2], [11], [20], and denticulated teeth are known to be efficient at slicing and cutting because they require less energy to penetrate food, thereby making larger and tougher organisms more energetically feasible prey items [11], [109], [110]. Finally, the high incidence of enamel spalling and crown apex breakage in *D. maximus* (Fig. 8) [17] is interesting when compared to recent work on the killer whale, which suggests that high incidences of crown breakage/apical wear may be due to a diet rich in abrasive-skinned chondrichthyans or a generalist diet of predominately suction-feeding whole fish [87], [88]. Differences in how ‘extreme’ tooth wear is between *Dakosaurus maximus* and killer whales can be explained through tooth replacement. Archosaurs, like *Dakosaurus*, have continual tooth replacement whereas odontocetes, like killer whales, are monophylodont (single set of teeth) [111].

In summary, *Dakosaurus* had a unique shearing occlusion pattern, a snout that was optimised for resisting torsional and bending stresses induced during prey capture; tooth crown bases that were, to some degree, protected from high feeding-induced stresses by ‘lateral plates’; macroscopically serrated tooth crowns, and crown apices that are frequently broken and spalled. All of the evidence presented above suggests that *Dakosaurus* used its teeth for cutting pieces small enough to swallow from large prey items, and had a skull that could resist the induced stresses involved with feeding on large and strong prey.

The craniodental morphology of *Plesiosuchus manselii* is in marked contrast to that of *Dakosaurus maximus*. *Plesiosuchus manselii* is distinct in having: a snout that is substantially wider than tall, with a concave dorsal margin; no ‘lateral plates’ on the dentigerous bones; serrated teeth with microscopic denticles; tooth crown apices that do not exhibit spalled surfaces or breaks; tooth crowns that do not exhibit occlusal wear facets on the mesial and distal carinae; and tooth-bearing bones lacking reception pits (Figs. 9, 10, 11, 12, 13, 14, 15, 16, 17, 18, 19, 20, 21, 22, 23; based on NHMUK PV OR40103, NHMUK PV R1089 and the two specimens in the Museum of Jurassic Marine Life, K181 and K434).

The lack of crown breakage and tooth wear (both spalling and carinal wear surfaces) is interesting. The presence of two sympatric macrophagous metriorhynchids that differ so markedly in tooth wear is intriguingly similar to what is observed in North Atlantic killer whales. There are two ‘types’ of North Atlantic killer whales: 1) ‘type 1′ is a small with extreme tooth wear, and 2) ‘type 2′ which is larger (maximum size is 2 m longer than ‘type 1′) and lacks tooth wear [87]. This is exactly what we see with *Dakosaurus maximus* and *Plesiosuchus manselii*: *D. maximus* is smaller with extreme apical wear (see Fig. 3E and 3F in [17]), and *P. manselii* lacks tooth wear and has a maximum size two metres greater than *D. maximus* (see description above, Fig. 30). In the North Atlantic, ‘type 1′ killer whales are generalists that suction-feed on whole fish (mackerel or herrings), although the sub-populations are known to feed on higher trophic levels such as seals; while ‘type 2′ killer whales are specialists that feed on other cetaceans. If the shared suite of morphofunctional characteristics between these killer whales and between *Dakosaurus* and *Plesiosuchus* are indicative of diet, then *Plesiosuchus* would be specialised in feeding on other marine reptiles and *Dakosaurus* would be a generalist and possible suction-feeder (the possibility of which is discussed below).

Furthermore, the difference in ‘optimum gape’ is considerable between *Plesiosuchus manselii* and *Dakosaurus maximus* (Fig. 31, Table 3). This suggests that these two species had distinct feeding ecologies. Unfortunately, without better preserved specimens we cannot attempt to reconstruct the occlusion mechanics of *Plesiosuchus manselii*. Regardless, the *Plesiosuchus* skull was not as well-suited to resist high stresses (in particular torsion) when compared to *D. maximus*, because it lacks an oreinirostral snout and ‘lateral plates’ on dentigerous bones; but coupled with its larger body-size (see above and [14]) and greater ‘optimum gape’ (Table 3) *Plesiosuchus* could have fed upon larger bodied prey. When directly comparing the ‘optimum prey depth’ at the same mandibular length (60 cm, see Table 3), there is a significant difference between *P. manselii* and *D. maximus* (12.6 cm vs 9 cm respectively). Interestingly, extant odontocete species that are sympatric and share a similar diet limit inter-specific competition by predating upon prey items of different size (such as the cephalopod specialists the sperm whale and the pygmy sperm whale, with the former predating upon larger-bodied cephalopod species than the latter) [112]. At the very least, it is clear that *D*. *maximus* and *P*. *manselii* had distinct morphologies and different sets of feeding-related characters, which may help explain why these two large-bodied crocodylomorphs were able to coexist in the same ecosystem.

### Adaptations for Macrophagy and Suction Feeding

Within Thalattosuchia two lineages exhibit adaptations towards macrophagy (feeding on large-bodied prey). Interestingly, these two lineages (the teleosaurid *Machimosaurus* and geosaurine metriorhynchids) share the same suite of morphofunctional adaptations [1], [2], [4], [10], [11], [17], [19], [20], [51], [62], [63], [103], [113], [114]:

1. Foreshortening of the snout (culminating in the brevirostrine condition)
2. Increase in snout width (snout wider than tall)
3. Reduction in dentition count (fewer than 20 teeth per tooth row)
4. Reduction in mandibular symphysis length (under 45% of total mandible length in *Machimosaurus*, under 35% in metriorhynchids)
5. Increase in width between the left and right jaw joints (high ratio of the maximum width from one quadrate to another, to basicranial length)
6. Increase in supratemporal fenestra size
7. Bicarinate teeth with serrated mesial and distal margins
8. Vertically orientated tooth crowns resulting in either interlocking occlusion or a “scissor-like” double-bladed arrangement
9. Increase in ‘optimum gape’ (achieved by ventral displacement of the dentary tooth row and disparity in size between anterior and posterior teeth in metriorhynchids, reduction in crown apicobasal length and disparity in size between anterior and posterior teeth in *Machimosaurus*)

In addition to possessing this suite of morphofunctional adaptations, *Dakosaurus* has a unique snout morphology: its snout is both amblygnathous and oreinirostral, with a very short mandibular symphysis (i.e. only the anterior-most mandibular teeth are adjacent to the symphysis) (Figs. 1, 2, 3). It is intriguing that *Dakosaurus* simultaneously possesses the macrophagous morphofunctional complex, an amblygnathous snout and a very short symphysis. Recent studies on cetacean craniomandibular evolution have discovered a morphofunctional complex for suction feeding–defined by Werth ([115]:580) as: “the creation of negative pressure in the oral or pharyngeal expansion or both to capture, ingest and transport discrete prey items” –in both odontocetes and fossil mysticetes [115], [116], namely:

1. Increase in snout width (the amblygnathous condition)
2. Reduction in dentition count
3. Reduction in mandibular symphysis length (very short, only the anterior-most mandibular teeth adjacent)
4. Increase in width between the left and right jaw joints (high ratio of the maximum width from one quadrate to another, to basicranial length)

Although two clades of thalattosuchians evolved macrophagous adaptations, only *Dakosaurus* evolved amblygnathy and the almost terminal mandibular symphysis seen in suction feeding odontocetes. In cetaceans, amblygnathy, short mandibular symphyses and widely separated jaw joints (potential expansion of the oesophagus) creates a larger oral cavity and a more circular mouth, thus improving water flow for suction feeding [115], [116]. *Dakosaurus maximus* is both amblygnathous and has a very short mandibular symphysis (Fig. 3), which combined with the ventrally displaced tooth-row and widely separated jaw joints ancestral to all geosaurins [1], [2] would have greatly enlarged the oral cavity and resulted in a more circular mouth.

Furthermore, the similarities in craniomandibular form between *Dakosaurus* and the basal mysticete cetacean *Janjucetus* are striking (low tooth count, serrated teeth, amblygnathous and oreinirostral snout, very short mandibular symphysis [115]). This morphology has been considered indicative of a raptorial/suction feeder [116]. We concur, and hypothesize that the species within the genus *Dakosaurus* may also be the first known suction feeding marine crocodylomorphs. This does not contradict *Dakosaurus* having a killer whale-style feeding ecology (see above and [17]), as juvenile killer whales can produce considerable suction, and further work is needed to determine if juvenile killer whales use suction during feeding and if adults do when predating on small prey items [115]. We note that most extant suction feeding cetaceans have their mouths delimited by the characteristic mammalian condition of lips or cheeks, which are important in controlling suction movements and formed by facial muscles. By virtue of their archosaurian ancestry, metriorhynchids most likely did not possess extensive lips and cheeks or facial muscles in general, at least of the mammalian variety. However, not all cetaceans control suction feeding using only lips and cheeks: the extant sperm whale *Physeter macrocephalus* lacks the facial muscles and soft tissues constraining mouth shape, but can generate gular pressure through use of the tongue [115]. Central to this ability is the possession of hyoids that are extremely large and flexible. Hyoids are currently unknown for *Dakosaurus*, but future discoveries will help to determine whether it may have also used a hyoid-driven method for generating negative pressure. Although the evidence for suction feeding in metriorhynchids is weak, due to the lack of facial muscles and information from a hyolingual apparatus, perhaps further studies of underwater feeding and the function of the palate in extant crocodylians will permit further exploration of how marine crocodylomorphs may have dealt with this functional constraint of living in a marine environment. As metriorhynchids have closer affinities to living aquatic taxa than any other fossil marine reptile group, they may be the most optimal group for exploring this functional system.

### Large-bodied Predators of the Kimmeridge Clay Sea

Although *Plesiosuchus manselii* is the largest known metriorhynchid, in the Kimmeridge Clay Formation of England there were numerous other marine reptiles that rivalled it in size. The ophthalmosaurid ichthyosaur genus *Brachypterygius* also attained large body size. The largest specimen had a mandibular length of 123 cm (CAMSM J68516), while a smaller specimen from Kimmeridge had a mandibular length of 82 cm (BRSMG Ce16696). *Brachypterygius* is characterised by: small orbit, long maxilla, robust lower and upper jaws, and large teeth [117]. The morphology of *Brachypterygius* is in contrast to that of a contemporaneous smaller ophthalmosaurid species, *Nannopterygius enthekiodon*, which had a proportionally long snout, large orbits and small teeth [118].

Presently, three giant pliosaur species are considered as present in the Kimmeridge Clay Formation: *Pliosaurus brachydeirus*, *P. portentificus* and *P. macromerus*. However, the taxonomy of these species is still highly uncertain [119], [120]. Confusion surrounds taxonomy at both the generic and specific levels, and about the referral of specimens to species with no overlapping elements. *Pliosaurus brachydeirus* (which may be the senior subjective synonym of *P*. *brachyspondylus* [121]) has the ‘long mandibular symphysis morphology’: 10–12 dentary alveoli adjacent to the symphysis. This morphology is observed in *P. brachydeirus*/*brachyspondylus* specimens (BRSMG Cc332, CAMSM J35991, OUMNH J9245B), the largest of which reached 1.7 m. *Pliosaurus portentificus* is known from three mandibles with eight dentary alveoli adjacent to the symphysis, the largest being two metres in length [119]. However, between *Pliosaurus brachydeirus*/*brachyspondylus* and *P. portentificus* there is continuous variation in the number of dentary alveoli adjacent to the symphysis. Noè *et al*. ([119]:22) mentions three French *P. brachyspondylus* specimens which have nine symphyseal alveoli, one of the key characters used to erect *P. portentificus*. As the *Pliosaurus macromerus* holotype (a large propodial) lacks mandibular material there is currently no justification in assigning the large mandibles with short symphyses to this species [119]. In fact, Noè *et al*. [119] could not dismiss a synonymy between *P. macromerus* and *P. portentificus*, or a synonymy between *P. macromerus* and the ‘long’ mandibular symphyseal *P. brachydeirus*.

Finally, there are the two very large pliosaur mandibles from the Kimmeridge Clay Formation, each with five/six dentary alveoli adjacent to the mandibular symphysis: NHMUK PV OR39510 (the *Pliosaurus grandis* skull and mandible described by Owen [54]) and OUMNH J10454. Although these specimens have been referred to *Pliosaurus macromerus*, this cannot currently be justified [119]. This taxon is possibly the largest predator of the Kimmeridge Clay Sea, with the mandible OUMNH J10454 being an estimated three metres in length when complete.

The Kimmeridge Clay Sea was curiously plentiful in large-bodied marine reptiles, of which pliosaurs were the largest organisms, growing to exceptional size. The large ichthyosaur *Brachypterygius* and *Plesiosuchus manselii* were comparable in size, both with cranial lengths exceeding one metre. Other metriorhynchid species from the Lower Kimmeridge Clay Formation (e.g. *Metriorhynchus geoffroyii/palpebrosus*, *Torvoneustes carpenteri* and *Dakosaurus maximus*) were significantly smaller is size (Fig. 30), with basicranial lengths of approximately 80 cm or less [14], [18], [47]. While the taxonomy of the marine reptiles of the Kimmeridge Clay Formation has yet to be settled, it is intriguing to consider how so many different clades (and species within those clades) co-existed. It is possible that variation in body size and craniodental morphology facilitated differences in resource acquisition, which enabled, and helped maintain, the stratification of available niches (as has been suggested for thalattosuchians [1], [2], [11], [13]–[16]). At the very least, as we argue above, such craniodental differences probably help explain how the two mid-to-large-sized metriorhynchids, *P*. *manselii* and the smaller *D*. *maximus*, were able to coexist.

These contentions are supported by a recent study on nine sympatric deep-diving odontocete species from the Bay of Biscay, which shows that they subdivide available niches on four criteria: 1) position in the water column, 2) prey type (predominately fish vs predominately cephalopod vs cephalopod-rich diet including pelagic crustaceans and/or pelagic tunicates), 3) prey size and 4) potentially prey quality (e.g. prey energy content) [112]. Although nine species are known to be deep-diving, a total of 19 odontocete species are known to be either resident (four confirmed and five suspected) or migratory (ten other species) in the Bay of Biscay [122]. Of the nine deep-diving odontocetes, all but one species is predominately teuthophagous [112]. Interestingly all ten sympatric raptorial shark species in the Bay of Biscay have a diet of ∼ 30% cephalopods [121]. The sharks also subdivide available niches, through position in the water column, body-size, lifestyle and feeding strategy [122].

### Conclusions

The crux of this paper is a systematic and anatomical revision of Late Jurassic European metriorhynchid crocodylomorph specimens that have historically been assigned to the aberrant, macrophagous genus *Dakosaurus*. Our focus is on two taxa in particular, *Dakosaurus maximus* and *Plesiosuchus manselii*, which we show are not particularly closely related (thus necessitating the resurrection of *Plesiosuchus* as a distinct genus). Based on overall morphological observation of cranial bones and teeth, we show that these two species have very different craniodental morphologies and functional ecologies.

*Dakosaurus maximus* has several characteristic features: an amblygnathous, brevirostrine and oreinirostral snout; premaxillary ‘lateral plates’; strongly ornamented maxillae; raised lateral and medial dentary alveolar margins; a very short mandibular symphysis; frequently broken crown apices; wear facets along the mesial and distal edges of the crown; and reception pits on the dentigerous bones of the upper and lower jaws. These features suggest that this species was adapted for dismembering large-bodied prey, had a unique occlusion pattern in which the upper and lower jaw teeth contacted each other mesiodistally in the sagittal plane and had a greatly enlarged oral cavity that may have enabled raptorial/suction feeding.

*Plesiosuchus manselii* is diagnosed by: strongly convex palatines with a pronounced ridge along the midline; a maxillopalatine suture that extends anteriorly from the suborbital fenestrae to the midline, anteriorly extending level to the fourth maxillary alveolus; the articular surface of the quadrate is not separated into two condyles by a sulcus; and tooth enamel ornamentation composed of apicobasal ridges of low relief, with serrations composed of microscopic true denticles. While *Plesiosuchus manselii* lacks many of the features seen in *D. maximus* that may have been the adaptations for dismembering large struggling prey, this species had a greater ‘optimum gape’ and was larger in size, comparable to the large-bodied Middle Jurassic pliosaur *Liopleurodon ferox*. Furthermore, the difference in enamel spalling and crown breakage between these species suggest that *Dakosaurus maximus* fed on abrasive food (such as sharks) or suction-fed, whereas *Plesiosuchus manselii* may have fed on other marine vertebrates. So, while *Plesiosuchus* may have consumed large prey, the prey size of *Plesiosuchus* was more likely to be limited by their own head size, whereas *Dakosaurus* could have fed on prey of sizes larger than its head because it could break them into smaller pieces.

These new observations, along with previous studies on other geosaurins (*Geosaurus* and *Torvoneustes*), indicate that while the genera in this subclade were specialized to feed on large-bodied prey (macrophagy) they were strongly differentiated in feeding style and ecology. The intriguing discovery that *Dakosaurus* may have been a suction feeder, like many extant odontocetes, further highlights that our understanding of Mesozoic marine ecosystems is still incomplete. Further examination of extant marine mammal analogues and crocodylomorphs, as well as equally important long-forgotten museum specimens will further elucidate the evolution of this remarkable group of marine tetrapods.

## Supporting Information

Text S1 **Characters and coding sources used in the phylogenetic analysis.** The document also has a list of supplementary references and a complete list of institutional abbreviations.
(DOC)

Text S2 **Character by taxon matrix used in the phylogenetic analysis (nexus file).**
(TXT)
