# Supplementary material for: The Cranial Osteology and Feeding Ecology of the Metriorhynchid Crocodylomorph Genera Dakosaurus and Plesiosuchus from the Late Jurassic of Europe
Source: PLoS One. 2012 Sep 18;7(9):e44985. doi: 10.1371/journal.pone.0044985 (PMC3445579; doi:10.1371/journal.pone.0044985)
Supplement: Text S1 — Characters and coding sources used in the phylogenetic analysis. The document also has a list of supplementary references and a complete list of institutional abbreviations. (DOC) [file pone.0044985.s001.doc]

# ONLINE SUPPLEMENTARY MATERIAL FOR:

**“The cranial osteology of the metriorhynchid crocodylomorph genera *Dakosaurus* and *Plesiosuchus* from the Late Jurassic of Europe”**

Mark T. Young^1^, Stephen L. Brusatte^2,3^, Marco Brandalise de Andrade^4^, Julia B. Desojo^5^, Brian L. Beatty^6^, Lorna Steel^7^, Marta S. Fernández^8^, Manabu Sakamoto^9^, Jose Ignacio Ruiz-Omeñaca^10,11^, Rainer R. Schoch^12^

^1^ School of Geosciences, University of Edinburgh, Grant Institute, The King’s Buildings, West Mains Road, Edinburgh, EH9 3JW, UK. zoologika@gmail.com

^2^ Division of Paleontology, American Museum of Natural History, Central Park West at 79^th^ Street, New York, NY 10024, U.S.A. sbrusatte@amnh.org

^3^ Department of Earth and Environmental Sciences, Columbia University, New York, New York, U.S.A.

^4^ Departamento de Paleontologia e Estratigrafia, Instituto de Geociências, Universidade Federal do Rio Grande do Sul – UFRGS, Av. Bento Gonçalves 9500, Porto Alegre (RS), Brazil, 91501-970, C.P. 15001. marcobranda@yahoo.com.br

^5^ División de Paleontología, Museo Argentino de Ciencias Naturales ‘Bernardino Rivadavia,’ Angel Gallardo 470, C1405DRJ, Buenos Aires, Argentina, CONICET, julideso@macn.gov.ar

^6^ Department of Anatomy, New York College of Osteopathic Medicine, Northern Boulevard, Old Westbury, NY 11568 U.S.A. bbeatty@nyit.edu

^7^ Department of Earth Sciences, Natural History Museum, Cromwell Road, London, SW7 5BD, UK. l.steel@nhm.ac.uk

^8^ Departamento Paleontología Vertebrados, Facultad de Ciencias Naturales y Museo, Universidad Nacional de La Plata, Paseo del Bosque s/n, La Plata 1900, Argentina. martafer@fcnym.unlp.edu.ar

^9^ School of Earth Sciences, University of Bristol, Wills Memorial Building, Queen’s Road, Bristol, BS8 1RJ, UK. m.sakamoto@bristol.ac.uk

^10^ Museo del Jurásico de Asturias. E-33328 Colunga, Spain. jigruiz@unizar.es

^11^ Departamento de Geología, Universidad de Oviedo, c/Jesús Arias de Velasco s/n, E-33005 Oviedo, Spain.

^12^ Staatliches Museum für Naturkunde Stuttgart, Rosenstein 1, D-70191 Stuttgart, Germany. rainer.schoch@smns-bw.de

1) Coding source for phylogenetic analyses

2) List of osteological characters used in the phylogenetic analyses

3) Supplementary References

4) Complete list of institutional abbreviations

**1) Coding source for phylogenetic analyses**

Table of species of thalattosuchians and other crocodylomorphs used in the phylogenetic analysis, including specimens examined first-hand. Type specimens are in bold, while casts are underlined.

**Outgroup taxon**

*Postosuchus kirkpatricki –* Nesbitt, 2011; Weinbaum, 2011.

**Ingroup taxa**

Sphenosuchia

*Dromicosuchus grallator* – Sues *et al*., 2003.

*Hesperosuchus agilis –* CM 29894; Clark *et al*., 2000.

*Terrestrisuchus gracilis* – Crush, 1984.

Protosuchia

*Protosuchus* – Colbert & Mook, 1951; Gow, 2000; Gasparini *et al*., 2006.

Basal Mesoeucrocodylia/Neosuchia

*Alligatorium* – HMN R.3632, Steel, 1973.

*Araripesuchus patagonicus* – MUCPv–267, MUCPv–268, MUCPv–268b, **MUCPv–269**, MUCPv–270; Ortega *et al.*, 2000.

*Calsoyasuchus valliceps* – Tykoski *et al*., 2002.

*Eutretauranosuchus* *delfsi* – **CM 8028**.

*Goniopholis simus* – NHMUK-PV R5814, **NHMUK PV OR41098**, **NHMUK PV OR41098a**.

*Goniopholis stovalli* – CMC VP7798.

*Pholidosaurus* – HMN R1965, HMN R1966, HMN R1969.1-3, HMN R2006.1-2, HMN R2009.1; Gasparini *et al*., 2006.

*Susisuchus anatoceps* – **SMNK 3804 PAL**, Salisbury *et al*., 2003.

*Theriosuchus guimarotae* – Schwarz & Salisbury, 2005.

Eusuchia

*Alligator mississippiensis* – NHMUK ZD 290, NHMUK ZD 1973-2-21-2, NHMUK ZD 1974-3010, NHMUK ZD 1975-1424, NHMUK ZD II-1-I.

*Crocodylus niloticus* – comparative collection held in the Palaeontology and Zoology departments of NHMUK; and in the Life Sciences Faculty, Ohio University.

*Crocodylus porosus* – comparative collection held in the Palaeontology and Zoology departments of NHMUK; and in the Life Sciences Faculty, Ohio University.

*Isisfordia duncani* – Salisbury *et al*., 2006.

NOTOSUCHIA

*Adamantinasuchus navae* – **UFRJ-DG-107-R**, UFRJ-DG-216-R, Nobre & Carvalho, 2006.

*Baurusuchus* – FEF-R-1/9; Price, 1945; Carvalho *et al*., 2005.

*Mariliasuchus amarali* – MN-6298-V, MN-6756-V, **UFRJ-DG-50-R**,UFRJ-DG-105-R, UFRJ-DG-106-R, UFRJ-DG-115-R, URC R.67, URC R.68, URC R.69, URC R.74, URC R.75; Carvalho & Bertini, 1999; Andrade, 2005; Vasconcellos & Carvalho, 2005.

*Notosuchus terrestris* – MACN–Pv–N–22, MACN–Pv–N–23, MACN–Pv–N–24, MACN–Pv–N–43, MACN–Pv–N–107, MACN–Pv–RN–1015, MACN–Pv–RN–1037, MACN–Pv–RN–1038, MACN–Pv–RN–1039, MACN–Pv–RN–1040, MACN–Pv–RN–1041, MACN–Pv–RN–1043, MACN–Pv–RN–1044, MACN–Pv–RN–1045, MACN–Pv–RN–1046, MACN–Pv–RN–1047, MACN–Pv–RN–1048, MACN–Pv–RN–1118, MACN–Pv–RN–1119, MLP–64–IV–16–1, **MLP–64–IV–16–5(253)**, MLP–64–IV–16–6(203), MLP–64–IV–16–7(219), MLP–64–IV–16–8(209), MLP–64–IV–16–10(221), MLP–64–IV–16–11, MLP–64–IV–16–12, MLP–64–IV–16–13, MLP–64–IV–16–14, MLP–64–IV–16–15, MLP–64–IV–16–16, MLP–64–IV–16–17, MLP–64–IV–16–18, MLP–64–IV–16–20, MLP–64–IV–16–21, MLP–64–IV–16–22, MLP–64–IV–16–23, MLP–64–IV–16–24, MLP–64–IV–16–25, MLP–64–IV–16–28, MLP–64–IV–16–30, MLP–64–IV–16–31(206); Woodward, 1896; Bonaparte, 1991; Andrade, 2005.

*Sphagesaurus* – DGM-1411-R, RCL-100, URC R.015, MPMA 15-001/90; Price, 1950; Pol, 2003; Andrade, 2005; Andrade & Bertini, 2008.

SEBECIA

*Hamadasuchus rebouli* – Larsson & Sues, 2007.

*Mahajangasuchus insignis* – Buckley & Brochu, 1999; Turner & Buckley, 2008.

*Montealtosuchus arrudacamposi* – Carvalho *et al*., 2007.

*Sebecus icaeorhinus* – AMNH 3160; Larsson & Sues, 2007.

*Uberabasuchus terreficus* – Carvalho *et al*., 2004.

Thalattosuchia: Teleosauroidea

*Machimosaurus hugii* – Buffetaut, 1982; Lepage et al., 2008

*Machimosaurus mosae* – Hua et al., 1993; Hua, 1999

*Platysuchus multiscrobiculatus* – **SMNS 9930**.

*Steneosaurus bollensis* – SMNS 849, SMNS 9427, SMNS 9428, SMNS 17484, SMNS 20280, SMNS 53422.

*Steneosaurus edwardsi* – NHMUK PV R2074, NHMUK PV R 2865, NHMUK PV R3701; Eudes-Deslongchamps, 1867-69.

*Steneosaurus leedsi* – NHMUK PV R2619, **NHMUK PV R3320**, NHMUK PV R3806.

*Steneosaurus megistorhynchus* – Eudes-Deslongchamps, 1867-69.

“*Steneosaurus*” *obtusidens* – **NHMUK PV R3168**.

*Teleosaurus cadomensis* – NHMUK PV OR119, NHMUK PV OR32588, NHMUK PV OR32657, NHMUK PV OR32680, NHMUK PV R880, NHMUK PV R880a; Jouve, 2009.

Thalattosuchia: Basal Metriorhynchoids

*Eoneustes bathonicus* – Mercier, 1933.

*Eoneustes gaudryi* – **NHMUK PV R3353**.

Metriorhynchoidea indeterminate (Chile) – Gasparini *et al*., 2000.

Metriorhynchoidea sp. (USA) – University of Iowa unnumbered.

*Pelagosaurus typus* – NHMUK PV OR19735, NHMUK PV OR32599, SMNS 8666, SMNS 17758, SMNS 50374, SMNS 80066.

*Teleidosaurus calvadosii* – NHMUK PV R2619; Eudes-Deslongchamps, 1867-69.

Thalattosuchia: Metriorhynchidae

*Cricosaurus araucanensis* – **MLP–72–IV–7–1**, MLP–72–IV–7–2; Herrera *et al*., 2009.

*Cricosaurus elegans* – **BSPG AS I 504**.

*Cricosaurus gracilis* – **OXFUM J1431**.

*Cricosaurus macrospondylus* – **HMN R3636. 1-6**, Hua *et al*., 2000.

*Cricosaurus saltillense* – Buchy *et al*., 2006.

*Cricosaurus schroederi* – Karl *et al*., 2006.

*Cricosaurus suevicus* – **SMNS 9808**, SMNS 90513.

*Cricosaurus* sp. (Cuba) – Gasparini & Iturralde-Vinent, 2001.

*Cricosaurus vignaudi* – Frey *et al*., 2002.

*Dakosaurus andiniensis* – Gasparini *et al*., 2006; Pol & Gasparini, 2009.

*Dakosaurus maximus* – NHMUK PV OR33186, NHMUK PV OR35766, NHMUK PV OR35835-7, **SMNS 8203**, SMNS 80148, SMNS 82043; Plieninger, 1846

Geosaurinae indeterminate (Argentina) – Gasparini *et al*., 2005.

*Geosaurus giganteus* – **NHMUK PV R1229**, NHMUK PV R1230, NHMUK PV OR37016, NHMUK PV OR37020.

*Geosaurus grandis* – **BSPG AS VI-1**.

*Geosaurus lapparenti* – Debelmas 1952, 1958; Debelmas & Strannoloubsky, 1957.

*Gracilineustes acutus* – Lennier, 1887.

*Gracilineustes leedsi* – CAMSM J64297, GLAHM V973, GLAHM V974, GLAHM V975, PETMG R24, PETMG R72, NHMUK PV R2031, NHMUK PV R2042, NHMUK PV R3014, NHMUK PV R3015, **NHMUK PV R3540**, NHMUK PV R3899, NHMUK PV R5793.

Metriorhynchinae indeterminate (Cuba) – USNM 419640.

*Metriorhynchus geoffroyii* – OXFUM J29823, OXFUM J55476-9.

*Metriorhynchus hastifer* – Eudes-Deslongchamps, 1867-69; Lepage *et al*., 2008.

*Metriorhynchus* sp. (France) – Buffetaut, 1977.

*Metriorhynchus superciliosus* – AMNH 997, GLAHM V942, GLAHM V963, GLAH V964, GLAHM V965, GLAHM V966, GLAHM V971, GLAHM V982, GLAHM V983, GLAHM V984, GLAHM V985, GLAHM V987, GLAHM V988, GLAHM V989, GLAHM V996, GLAHM V1004, GLAHM V1015, GLAHM V1027, GLAHM V1140, GLAHM V1142, GLAHM V1143, NHMUK PV R1666, NHMUK PV R2030, NHMUK PV R2032, NHMUK PV R2036, NHMUK PV R2044, NHMUK PV R2051, NHMUK PV R2053, NHMUK PV R2054, NHMUK PV R2055, NHMUK PV R2058, NHMUK PV R2067, NHMUK PV R3900, NHMUK PV R6859, NHMUK PV R6860, PETMG R10, PETMG R17, PETMG R18, PETMG R20, PETMG R42, PETMG R180, RMS M150, SMNS 10115, SMNS 10116, SMNS 81689.

*“Metriorhynchus” brachyrhynchus* – GLAHM V978, GLAHM V995, NHMUK PV R1711, , NHMUK PV R2618, NHMUK PV R3051, NHMUK PV R3321, NHMUK PV R3699, **NHMUK PV R3700**, NHMUK PV R3804, NHMUK PV R3939, NHMUK PV R4763, PETMG R19.

*“Metriorhynchus” casamiquelai* – Gasparini & Chong, 1977.

*“Metriorhynchus” westermanni* – Gasparini *et al*., 2008; Fernández *et al*., 2011.

“Mr Leeds’ specimen” – **GLAHM V972**, Young *et al*., in press.

*Neptunidraco ammoniticus* – Cau & Fanti, 2011.

*Plesiosuchus manselii* – **NHMUK PV OR40103, NHMUK PV OR40103a**, NHMUK PV R1089.

*Rhacheosaurus gracilis* – AMNH 4804, NHMUK PV R3961, NHMUK PV R3948.

*Suchodus durobrivensis* – **NHMUK PV R1994**, NHMUK PV R2039.

*Torvoneustes* *carpenteri* – **BRSMG Ce17365**, BRSMG Cd 7203.

**2) List of osteological characters used in the phylogenetic analyses**

List of the 240 osteological characters used in the phylogenetic analysis presented herein. The characters are organised in anatomical order: rostrum; skull roof; orbit and temporal region; palate and perichoanal structures; occipital; braincase, basicranium and suspensorium; mandible; dentition; appendicular skeleton; axial skeleton; and then osteoderms. Cranial, mandibular and dental characters make up 73% (175/240) of the character list, while the post-cranial characters contribute 27% (65/240).

40 multi-state characters can be treated as ordered (transformational sequences), they are: 1^st^, 7^th^, 8^th^, 10^th^, 13^th^, 25^th^, 38^th^, 39^th^, 42^nd^, 43^rd^, 47^th^, 50^th^, 56^th^, 58^th^, 69^th^, 86^th^, 87^th^, 96^th^, 126^th^, 132^nd^, 133^rd^, 151^st^, 152^nd^, 154^th^, 156^th^, 166^th^, 179^th^, 181^st^, 182^nd^, 183^rd^, 184^th^, 198^th^, 202^nd^, 214^th^, 218^th^, 225^th^, 228^th^, 230^th^, 231^st^ and 237^th^ characters.

**Rostrum**

| Character | Description |
| --- | --- |
| 1 | **Skull width to length ratio (NEW) (ORDERED):**  *= maximum width between the lateral-most points of the quadrates : basicranial length*  0. 0.26 or lower  1. between 0.27 and 0.4  2. 0.4 or greater |
| 2 | **Rostrum cross-section:**  0. nearly tubular (lateromedial & dorsoventral axes subequal ±5%)  1. wider than tall (dorsoventral axis 120% or more of the lateromedial axis)  2. taller than wide (dorsvoventral axis greater than lateromedial axis) |
| 3 | **Rostrum, in dorsal view – amblygnathy (“bullet-shaped”, with the rostrum retaining its width along almost all its length) (NEW):**  *State (1) is an apomorphy of* Dakosaurus.  0. no  1. yes |
| 4 | **Sculpture on external surface of rostrum (premaxilla and maxilla):**  0. no conspicuous ornamentation, or ornamented with an irregular pattern of ridges rugosities and anastomosing grooves  1. conspicuous pitted (circular-to-polygonal) pattern  2. conspicuous grooved pattern |
| 5 | **Tooth row, premaxillary alveoli and posterior maxillary alveoli:**  0. entire upper tooth row in the same plane  1. posterior maxillary alveoli ventral to all other alveoli |
| 6 | **External nares, shape in dorsal view:**  0. subcircular (diameter in any direction does not vary by more than ± 10%)  1. oval (dorsal width >10% longer than antero-posterior length)  2. ‘D-shaped’, with posterior edge straight  3. spoon-shaped elongate ellipse (dorsal width <40% of antero-posterior length)  4. external nares not exposed in dorsal view |
| 7 | **External nares, posterodorsal retraction in relation to the tooth-row (ORDERED):**  *This character was designed to quantify the degree of posterodorsal retraction of the external nares in Metriorhynchidae. Its level relative to the tooth-row is used in this regard.*  0. at the tip of the snout, with its posterior-margin not exceeding the first premaxillary alveolus  1. at the tip of the snout, but its posterior-margin does exceed the last premaxillary alveolus  2. the posterior-margin reaches to the beginning of the 1st maxillary alveolus  3. anterior-margin starts just after the 1st premaxillary alveolus while the posterior-margin does not exceed the 1st maxillary alveolus  4. posterior-margin reaches to approximately the end of the 2nd maxillary alveolus  5. anterior-margin begins from the start of the 2nd premaxillary alveolus with the posterior-margin terminating ventral to midway through the 3rd maxillary alveolus  6. posterodorsally displaced, with the external nares anterior-margin beginning posterior to the 3rd premaxillary with the posterior-margin terminating beyond the 3rd maxillary alveolus |
| 8 | **Premaxilla, proportion of total length posterior to the external nares (ORDERED):**  0. greater than 67% of premaxilla total length is posterior to the external nares  1. between 50-65%  2. between 36-45%  3. 28%, or less |
| 9 | **Premaxilla, development of premaxillary septum (MODIFIED):**  *Character changed from multistate to binary*.  0. no septum, with a single undivided nasal cavity  1. nasal cavity bifurcated by a premaxillary septum |
| 10 | **Distance between premaxilla and nasal (NEW) (ORDERED):**  0. none, premaxilla and nasal contact  1. small, less than half the midline length of the premaxilla  2. large, ~ 80% to more than 100% of the midline length of the premaxilla |
| 11 | **Nasal contribution to the margin of the external nares :**  0. present  1. absent |
| 12 | **Nasals, outline in dorsal view:**  *State (1) is an apomorphy of both Thalattosuchia and Notosuchia.*  0. rectangular, with lateral margins mostly parallel  1. clearly triangular |
| 13 | **Nasals, dorsal surface close to the posterior end (Andrade 2010, ch.75) (ORDERED):**  *State (2) is an apomorphy of* Peipehsuchus + “Steneosaurus” brevior + Pelagosaurus *+ Metriorhynchidae.*  0. continuous, lacking a medial groove or trench  1. slightly concave, forming a shallow and poorly defined depression, anteroposteriorly oriented  2. deeply trenched, with a steep longitudinal depression at midline (the trench is formed as both nasals are separately convex) |
| 14 | **Nasal contact with the prefrontal, in dorsal view:**  *State (1) is an apomorphy of the* Cricosaurus *subclade: (*C. araucanensis *+ (*C. schroederi *+* C. macrospondylus*).*  0. irregular  1. smooth curve with a concavity directed posterolaterally |
| 15 | **Nasal-prefrontal contact:**  0. Absent  1. Present |
| 16 | **Premaxilla–maxilla lateral fossa excavating alveolus of last premaxillary tooth:**  0. no  1. yes |
| 17 | **Maxilla, ventrolateral edge:**  0. straight  1. single convexity  2. double convexity (‘festooned’) |
| 18 | **Maxilla, depression on the lateral surface:**  *State (1) is an apomorphy of Goniopholididae*  0. no  1. yes |
| 19 | **Maxilla, aligned set of large foramina extending posteroventrally from the antorbital/preorbital fossa, interconnected through a shallow groove:**  0. no  1. yes |
| 20 | **Maxilla-lacrimal, contact:**  0. partially included in antorbital/preorbital fossa  1. completely included |
| 21 | **Lacrimal, contact with the nasal:**  0. dorsal edge of lacrimal only  1. primarily the anterior edge of the lacrimal  2. no contact |
| 22 | **Nasal-lacrimal suture, length compared to nasal-prefrontal suture (in dorsal view):**  *State (0) of the original formation removed, as it is redundant due to state (2) of the previous character.*  0. long, subequal or longer than naso-prefrontal suture  1. short, 60% or less than naso-prefrontal suture |
| 23 | **Lacrimal, dorsal exposure:**  0. present, can be observed in both dorsal and lateral view  1. absent, only visible in lateral view (lacrimal vertically orientated) |
| 24 | **Lacrimal, size:**  0. large, in lateral view at least 45% of orbit height  1. small, less than 40% of orbit height |
| 25 | **Antorbital fenestra, size and presence (ORDERED):**  *The absence of the antorbital fenestra (state 2) occurs independently numerous times in the evolution of Crocodylomorpha. Within Thalattosuchia, all early Jurassic taxa possess antorbital fenestrae. By the Middle Jurassic no taxon retain these fenestrae, the only currently known exception is* Teleosaurus cadomensis*.*  0. at least half the diameter of the orbit  1. much smaller than the orbit  2. absent |
| 26 | **Antorbital fenestra, bones enclosing (nasal): (NEW)**  *Modified as the metriorhynchid character states relating to the antorbital fenestra/fossa have been excluded. This is due to hypothesis 2 of Fernández & Herrera (2009), in which the antorbital cavity is internalised in metriorhynchids. The opening classically referred as the “*antorbital fenestra*” in this clade is in fact a neomorphic preorbital opening for the excretion of salt.*  0. nasal does not contribute to the antorbital fenestra  1. nasal does contribute to the antorbital fenestra |
| 27 | **Antorbital fenestra, bones enclosing (jugal): (NEW)**  *Similar to the previous character, except it codes for the jugal participation in the antorbital fenestrae rather than the nasal.*  0. jugal does not contribute to the antorbital fenestra  1. jugal does contribute to the antorbital fenestrae |
| 28 | **Antorbital fossa, shape:**  *Modified as the metriorhynchid character states relating to the antorbital fenestra/fossa have been excluded. This is due to hypothesis 2 of Fernández & Herrera (2009), in which the antorbital cavity is internalised in metriorhynchids. The opening classically referred as the “*antorbital fenestra*” in this clade is in fact a neomorphic preorbital opening for the excretion of salt.*  0. subcircular or subtriangular  1. elongated |
| 29 | **Antorbital fossa, bones enclosing (nasal): (NEW)**  0. nasal does not contribute to the antorbital fossa  1. nasal does contribute to the antorbital fossa |
| 30 | **Antorbital fossa, bones enclosing (jugal): (NEW)**  *Similar to the previous character, except it codes for the jugal participation in the antorbital fossa rather than the nasal.*  0. jugal does not contribute to the antorbital fossa  1. jugal does contribute to the antorbital fossa |
| 31 | **Preorbital fenestra (not homologous to archosaurian antorbital fenestra), presence:**  *Herein we follow hypothesis 2 of Fernández & Herrera (2009), in which the antorbital cavity is internalised in metriorhynchids. The opening classically referred as the “*antorbital fenestra*” in this clade is in fact a neomorphic preorbital opening for the excretion of salt. This opening is connected via ducts to a chamber which housed large salt-glands (see Fernández & Herrera, 2009). This fenestra is bound by an elongate, narrow and obliquely orientated fossa bound by the lacrimal, nasal and maxilla.*  0. absent  1. present |
| 32 | **Antorbital fenestra, height:**  *Character re-phrased as referring to the antorbital fenestra, and is therefore cannot be coded for metriorhynchids.*  0. approximately as tall as the height between the tooth row to the ventral rim of the fenestra (±10%)  1. less than the height between the tooth row to the ventral rim of the fenestra |
| 33 | **Prefrontal-lacrimal fossae:**  *The prefrontal-lacrimal fossa (sensu Young & Andrade, 2009) refers to a shallow depression immediately anterior to the orbit, present on both the prefrontal and lacrimal. It is situated posterior to the preorbital fenestra, and never contacts the preorbital fossa. There is a crest within this fossa that is present along the prefrontal-lacrimal contact. State (1) is an apomorphy of Metriorhynchidae.*  0. absent  1. present, with ridge following the sutural contact between these elements |

Skull roof

| Character | Description |
| --- | --- |
| 34 | **Skull roof:**  0. complex  1. dorsally flat ‘skull table’ developed |
| 35 | **Posterior skull table:**  *Note that* Sphagesaurus *codes differently in this character, and for the preceding character.*  0. non-planar (squamosal ventral to horizontal level of postorbital and parietal)  1. planar (postorbital, squamosal, and parietal on same horizontal plane) |
| 36 | **Cranial table width relative to ventral portion of skull:**  0. nearly as wide  1. narrower |
| 37 | **Mature skull table, with broad lateral curvature:**  0. short caudolateral process of the squamosal  1. mature skull table with nearly horizontal sides; significant caudolateral process of the squamosal |
| 38 | **Supratemporal fossa, anterior margin in dorsal view (ORDERED):**  *This character was designed to quantify the rostral extent of the supratemporal fossae. In Metriorhynchidae, the fossae begin to invade the dorsal surface of the orbital region. In both* Dakosaurus *and* Cricosaurus sp. (Mexico)*, the intratemporal flanges of the supratemporal fossae extend as far rostrally as the interorbital minimum distance (state 3).*  0. anterior margin posterior to the postorbital  1. anterior margin reaches between the anterior and posterior points of the frontal-postorbital suture  2. reaches at least as anteriorly as the postorbital  3. projects more anteriorly than the postorbital and reaches the interorbital minimum distance |
| 39 | **Supratemporal fossae, shape, anteroposterior and lateromedial axes: (ORDERED) (NEW)**  *In Thalattosuchia, state (1) is an apomorphy of* Teleosaurus cadomensis  0. longitudinal ellipsoid/sub-rectangular (anteroposterior axis more than 10% longer than the lateromedial axis)  1. sub-square/sub-circular (anteroposterior and lateromedial axes subequal, ± 5%)  2. transverse ellipsoid/sub-rectangular (lateromedial axis more than 10% longer than the anteroposterior axis) |
| 40 | **Supratemporal fossae, teardrop-shape (lateral and posterior margins of the fossae form a continuous curve, i.e. no distinct angle where the two margins meet): (NEW)**  *State (1) is an apomorphy of derived species of* Cricosaurus *(e.g.,* C. araucanensis*)*  0. no  1. yes |
| 41 | **Supratemporal fossae, shape, parallelogram (lateral and medial margins, and anterior and posterior margins are sub-parallel): (NEW)**  *State (1) is an apomorphy of* Machimosaurus  0. no  1. yes |
| 42 | **Supratemporal fenestra, in dorsal view, size relative to orbits (ORDERED):**  0. longer in length than the orbit (supratemporal length 110% or more of orbit length)  1. subequal in length as the orbit (± 5%)  2. smaller than the orbits (supratemporal length less than 90% of orbit length) |
| 43 | **Supratemporal fenestra, in dorsal view, posterior limit (ORDERED):**  *State (2) is an apomorphy of Geosaurini.*  0. terminates well before the posterior-most point of the parietal  1. either terminates near the posterior-most of the parietal or exceeds it, but never reaches the supraoccipital  2. more posterior than intertemporal bar |
| 44 | **Supratemporal fenestrae, intratemporal flange presence:**  *Intratemporal flange is the rostral component of the supratemporal fossa that is a “shelf”. Namely, anterior to the fenestra itself and invades the rostral part of the skull roof/orbital region.*  *In Thalattosuchia, state (1) is an apomorphy of* Eoneustes *+ Metriorhynchidae.*  0. absent  1. present |
| 45 | **Supratemporal arch, medial margin in dorsal view:**  *State (1) is an apomorphy of* Metriorhynchus *sp. +* Metriorhynchus hastifer*, and of a subclade within* Cricosaurus *(all cricosaurs other than the Cuban indeterminate specimen from the Oxfordian).*  0. not convex  1. convex |
| 46 | **Supratemporal arch, dorsal margin in lateral view:**  0. concave  1. straight  2. convex |
| 47 | **Prefrontal, lateral development (ORDERED):**  *The transverse development of the prefrontal is a classic characteristic of Metriorhynchidae.*  *State (1) is an apomorphy of* Eoneustes*.*  *State (2) is an apomorphy of Metriorhynchidae.*  0. reduced, flush with the rim of the orbit  1. incipient enlargement (extending laterally over the orbit by approximately 5% of its width)  2. enlarged (extending laterally over the orbit by >15% of its width) |
| 48 | **Prefrontal, lateral development relative to the posterolateral corner of the supratemporal fossa in dorsal view:**  0. Prefrontal does not expand laterally so that it is in the same plane as the posterolateral corner of the supratemporal fossa  1. Prefrontal expands further laterally than the posterolateral corner of the supratemporal fossa |
| 49 | **Prefrontal, shape in dorsal view: (NEW)**  *Sstate (1) is an apomorphy of Metriorhynchidae.*  0. quadrilateral with irregular outline  1. teardrop-shaped |
| 50 | **Prefrontal, morphology of the lateral border in dorsal view (ORDERED): (NEW)**  *This character describes the shape of the prefrontal in Metriorhynchidae.* Plesiosuchus*,* Geosaurus *and* Torvoneustes *code as state (1). State (2) is an apomorphy of* Dakosaurus*.*  0. continuous convex curve, inflexion point approximately 80-90 degree angle from the anteroposterior axis of the skull  1. continuous convex curve, inflexion point approximately70 degree angle from the anteroposterior axis of the skull  2. continuous convex curve, inflexion point approximately 50 degree angle from the anteroposterior axis of the skull |
| 51 | **Prefrontal, dimensions in dorsal view:**  0. longer than wide  1. length/width is subequal (± 5%) |
| 52 | **Prefrontal, anterior to the orbits:**  0. elongate, oriented parallel to antero-posterior axis of the skull  1. short and broad |
| 53 | **Prefrontal, nasal-prefrontal suture has a pronounced, rectangular ‘concavity’ (directed posteriorly):**  *State (1) is an apomorphy of* Eoneustes*.*  0. absent  1. present |
| 54 | **Prefrontal, nasal-prefrontal suture has a posteriorly directed ‘V’-shape:**  *State (1) is an apomorphy of* Cricosaurus macrospondylus *and* C. schroederi*.*  0. absent  1. present |
| 55 | **Frontal, ornamented:**  0. yes, shallow to deep elliptical pits  1. yes, shallow grooves aligned radially  2. no |
| 56 | **Frontal, angle between medial and lateral posterior processes (ORDERED):**  0. approximately 90 degree angle, or obtuse  1. approximately 70-60 degree angle  2. approximately 45 degree angle, or more acute |
| 57 | **Frontal, minimum width between orbits in dorsal view compared to the supratemporal fossa:**  0. greater than the width of one supratemporal fossa and the intertemporal bar  1. subequal to width of one supratemporal fossa |
| 58 | **Frontal, minimum width between orbits in dorsal view compared to the orbits (ORDERED):**  0. broader than orbital width  1. subequal with orbital width  2. narrower than orbital width |
| 59 | **Frontal-parietal between supratemporal fossa in dorsal view:**  0. frontal and parietal subequal in width (± 5%)  1. parietal width is greater than 75% of frontal width |
| 60 | **Frontal-postorbital suture:**  0. level with the intertemporal bar  1. lower than the intertemporal bar |
| 61 | **Frontal-postorbital suture in dorsal view:**  *State (1) is a metriorhynchid apomorphy.*  0. irregular and straight or gently curved  1. frontal splitting the postorbital in a ‘V’-shape directed posteriorly |
| 62 | **Postorbital, shape in dorsal view:**  0. the outer margin is convex where the postorbital curves posteriorly forming the supratemporal arch  1. forming a 90 degree angle  2. rostral extension from the corner |
| 63 | **Postorbital, anterolateral extension:**  *State (1) and state (2) of character 57 (rostral extension from the postorbital corner) does not necessary occur in the same taxon (e.g.,* Oceanosuchus*).*  0. small or absent  1. very large, appearing in lateral view to contact the dorsal surface of the jugal |
| 64 | **Postorbital and squamosal, relative lengths in dorsal view:**  0. squamosal is longer  1. postorbital is longer |
| 65 | **Squamosal, projects further posteriorly than the occipital condyle:**  0. no  1. yes |
| 66 | **Squamosal, contribution to the supratemporal arch:**  0. 40% or less  1. at least 50% |
| 67 | **Dorsal and ventral rims of squamosal groove for external ear-flap musculature:**  0. absent  1. ventral rim placed lateral to dorsal rim  2. ventral rim directly beneath dorsal rim |
| 68 | **Parietal-squamosal, posterior margin in dorsal view:**  *State (1) is an apomorphy of Rhacheosaurini*  0. concave curve  1. largely straight, but the parietal forms distinct ‘U’-shape with the concave margin directed towards the occipital condyle  2. parietal posterior margin is convex |

Orbit and temporal region

| Character | Description |
| --- | --- |
| 69 | **Orbit, position (ORDERED):**  0. fully dorsal  1. mainly dorsal, but with slight inclination  2. lateral, but slightly inclined dorsally, usually visible in dorsal view  3. fully lateral with orbit shape only clear in lateral view |
| 70 | **Orbit, shape:**  0. circular, anteroposterior and dorsoventral axes subequal (± 5%)  1. longitudinal ellipsoid, anteroposterior axis more than 10% longer than dorsoventral axis  2. transverse ellipsoid, dorsoventral axis more than 10% longer than anteroposterior axis |
| 71 | **Orbit, anterodorsal margin and the lacrimal: (NEW)**  *In Thalattosuchia, state (1) is an apomorphy of* Teleidosaurus calvadosii  0. lacrimal is excluded from the orbit anterodorsal margin  1. lacrimal reaches the orbit anterodorsal margin |
| 72 | **Orbit, posterodorsal margin and the postorbital: (NEW)**  *In Thalattosuchia, state (1) is an apomorphy of the clade* Teleidosaurus *+ Metriorhynchidae*  0. postorbital is excluded from the orbit posterodorsal margin  1. postorbital reaches the orbit posterodorsal margin |
| 73 | **Orbit, anteroventral margin and the lacrimal: (NEW)**  0. lacrimal is excluded from the orbit anteroventral margin  1. lacrimal reaches the orbit anteroventral margin |
| 74 | **Orbit, posteroventral margin and the postorbital: (NEW)**  *In Thalattosuchia, state (1) is an apomorphy of* Platysuchus multiscrobiculatus + Teleosaurus cadomensis  0. postorbital is excluded from the orbit posteroventral margin  1. postorbital reaches the orbit posteroventral margin |
| 75 | **Orbit, ventral margin and the jugal: (NEW)**  *In Thalattosuchia, state (1) is an apomorphy of* Platysuchus multiscrobiculatus  0. jugal participates in the orbit ventral margin  1. jugal excluded from the orbit by lacrimal-postorbital contact |
| 76 | **Palpebrals:**  0. two palpebrals in orbit  1. one large palpebral  2. absent |
| 77 | **Sclerotic ossicles (composing the sclerotic ring):**  *Within Thalattosuchia, state (1) is an apomorphy of* Pelagosaurus *+ Metriorhynchidae.*  0. absent  1. present |
| 78 | **Jugal, width of anterior process relative to posterior process:**  0. subequal  1. about twice as broad |
| 79 | **Jugal, extends rostrally in front of the prefrontal:**  0. no  1. yes |
| 80 | **Jugal, base of postorbital process in lateral view:**  0. directed posterodorsally  1. dorsally |
| 81 | **Postfrontal (NEW):**  *State (1) is an apomorphy of Crocodylomorpha*  0. Present  1. Absent |
| 82 | **Postorbital bar, morphology of dorsal end:**  0. dorsal end of the postorbital bar broadens dorsally, continuous with dorsal part of the postorbital  1. dorsal part of the postorbital bar constricted, distinct from the dorsal part of the postorbital |
| 83 | **Postorbital bar, vascular opening on lateral edge of dorsal part:**  0. absent  1. present |
| 84 | **Postorbital bar, morphology of postorbital-jugal contact:**  0. postorbital medial to jugal  1. postorbital lateral to jugal |
| 85 | **Postorbital bar, cross-section:**  0. transversely flattened  1. cylindrical |
| 86 | **Infratemporal fenestra (=laterotemporal fenestra), in lateral view (ORDERED):**  0. considerably longer in length than the orbit (greater than 25%)  1. equal/subequal in length than the orbit (± 10%)  2. shorter in length than the orbit (less than 25%) |
| 87 | **Spina quadratojugalis (ORDERED):**  0. absent  1. either small or low crest  2. prominent |

**Palate and perichoanal structures**

| Character | Description |
| --- | --- |
| 88 | **Palatal surface of the rostrum, notch near premaxilla-maxilla suture: (NEW)**  0. absent  1. present, lozenge-shaped |
| 89 | **Palatal surface of the rostrum, naso-oral fossa/fenestra: (NEW)**  0. absent  1. present, usually round or elliptic in shape |
| 90 | **Maxilla-palatine contact, presence (NEW):**  *Character helps to quantify the development of the secondary palate.*  0. Absent  1. Present |
| 91 | **Palatine, anterior margin has a mid-line anterior process (NEW):**  0. present  1. absent |
| 92 | **Palatine, mid-line anterior process shape, in palatal view (NEW):**  0. lateral margins of the mid-line anterior process converge: anteriorly orientated “V”-shape  1. lateral margins of the mid-line anterior process largely parallel: anteriorly orientated “U”-shape |
| 93 | **Palatine, anterior margin has two non-midline anterior processes (NEW):**  *In Thalattosuchia, state (1) is an apomorphy of Metriorhynchinae.*  *In* Montealtosuchus *and* Hamadasuchus *the mid-line anterior process has a concave anterior margin, creating two “non-midline” processes*  0. Absent  1. Present |
| 94 | **Palatine, has a very large mid-line anterior process and at the suborbital fenestrae the palatine anterior margin curves rostrolaterally towards it, creating two “small processes”:**  *This morphology is variably observed in Eusuchia, Dyrosauridae and Pholidosauridae.*  0. no  1. yes |
| 95 | **Palatine, form secondary palate:**  0. palatines of primary palate exposed and do not contact one another secondarily on mid-line  1. palatines meet on mid-line forming a secondary palate |
| 96 | **Pterygoid, secondary palate (ORDERED):**  0. absent  1. thin shelves not meeting  2. present |
| 97 | **Palatine-pterygoid suture, lateral protrusions by palatine into the pterygoids:**  0. absent  1. present |
| 98 | **Pterygoid flange, orientation (in palatal view):**  0. horizontal  1. largely horizontal, but clearly rostrally inclined  2. strongly projecting caudally |
| 99 | **Pterygoid, delimits the posterior margin of the choanae:**  0. no, or not well-delimits the posterior margin of the choane  1. yes |
| 100 | **Choanal opening, in palatal view:**  *State (1) is observed in extant species*  0. choanal opening orientated posteriorly, enclosed ventrally by the palatine and by the pterygoid dorsally  1. choana opens into palate through a deep midline depression (choanal groove) |
| 101 | **Choana, anterior margin shape:**  0. semicircular or elliptical  1. ‘V’-shaped with its base directed anteriorly  2. broad ‘U’-shaped with its base directed anteriorly  3. ‘W’-shaped with its base directed anteriorly |

Occiptial

| Character | Description |
| --- | --- |
| 102 | **Supraoccipital, posterior surface:**  0. nearly flat  1. two lateral prominences |
| 103 | **Supraoccipital, contribution to the border of the foramen magnum:**  *Character 19 from Gower (2002)*.  0. excluded from dorsal border of foramen magnum by mediodorsal midline contact between opposite exoccipitals  1. contributes to border of foramen magnum |
| 104 | **Paroccipital process, orientation in occipital view:**  *State (1) is an apomorphy of Rhacheosaurini.*  *State (2) is an apomorphy of a subclade of Geosaurinae.*  *State (3) is an apomorphy of Dyrosauridae.*  0. horizontal  1. dorsolaterally orientated, at a 45 degree angle  2. ventral-edge horizontal, then terminal third sharply inclined dorsolaterally at a 45 degree angle  3. prominently arch ventrally |
| 105 | **Paroccipital process, large ventrolateral region:**  0. present  1. absent |
| 106 | **Paroccipital process, overlap by the squamosal:**  0. small: the squamosal does not extend more posteriorly than the paroccipital process  1. large: it extends further posteriorly than the paroccipital process |
| 107 | **Foramen for cranial XII nerve, position on occipit:**  0. above the occipital condyle in line with the foramen magnum  1. below the foramen magnum |
| 108 | **Foramen for the internal carotid artery, size:**  *State (1) is an apomorphy of* Pelagosaurus *+ Metriorhynchidae.*  0. similar in size to the openings for cranial nerves IX-XI  1. extremely enlarged |
| 109 | **Occipital surface ventral to occipital condyle:**  0. slopes rostroventrally  1. roughly parallel to the transverse plane |

**Braincase, basicranium and suspensorium**

| Character | Description |
| --- | --- |
| 110 | **Trigeminal fossa (=fossa for cranial nerve V), development on quadrate and laterosphenoid:**  *Character based the discovery by Fernández et al. (2011)*.  *State (1) is an apomorphy of Metriorhynchidae.*  0. developed rostrally and caudally to the trigeminal fenestra (i.e. fossa present on both laterosphenoid and quadrate)  1. fossa is mainly developed caudally to the fenestra (i.e. fossa present on quadrate) |
| 111 | **Supratemporal fossae, laterosphenoidprootic suture forms a blunt crest creating two distinct fossae for muscle attachment:**  *Character based upon data from Holliday & Witmer (2009) and Fernández et al. (2011).*  0. yes  1. crest absent |
| 112 | **Parasphenoid ridge/rostrum (?), in palatal view:**  *The homology of this ridge is unknown. Andrews (1913) considered the midline pterygoid ridge to be the parasphenoid. However, the pterygoids are poorly known for metriorhynchids, and we cannot discount this as a purely pteryoid structure. Until this structure has undergone CT scanning we will provisionally use the term parasphenoid for this structure.*  0. not visible  1. forms a midline ridge along the pterygoids |
| 113 | **Basisphenoid, paired ridges located medially on the ventral surface:**  0. absent  1. present |
| 114 | **Basisphenoid, exposure in palatal view:**  0. exposed on ventral surface of braincase  1. virtually excluded from ventral surface by pterygoid and basioccipital |
| 115 | **Basisphenoid, exposure anterior to the quadrates in palatal view (RE-PHRASED):**  *State (1) is an apomorphy of a subgroup of “teleosaurids”. This character state is caused by the caudal expansion of the pterygoid’s posterior margin, so that the anterior portion of the quadrates is obscured, as are the lateral margins of the basisphenoid. However, there is a distinct basisphenoid ‘rostrum’ that in some taxa continue to bifurcate the ptergoids rostrally. This morphology is not observed in* Teleosaurus cadomensis*,* Peipehsuchus teleorhinus, Steneosaurus brevior, Pelagosaurus typus *or Metriorhynchidae.*  0. basisphenoid terminates approximately level to the anterior extent of the quadrates  1. basisphenoid ‘rostrum’ exposed along the palatal surface anterior to the quadrates, continuing to bifurcating the pterygoids |
| 116 | **Basisphenoid, exposure ventral to the basioccipital at maturity in occipital aspect:**  0. absent, pterygoid dorsoventrally short ventral to median eustachian  1. present, pterygoid dorsoventrally tall ventral to median eustachian opening |
| 117 | **Basioccipial , basal tubera:**  0. reduced  1. large and pendulous |
| 118 | **Quadrate, prominent crest on dorsal surface of distal quadrate extending proximally to lateral extent of quadrate–exoccipital contact:**  0. absent  1. present |
| 119 | **Quadrate, dorsal primary head contact:**  0. only squamosal  1. squamosal and (or near) laterosphenoid |
| 120 | **Quadrate orbital process, remains free of bony attachment along its rostromedial surface:**  *This character represents the ‘quadrate incompletely sutured to the braincase’ statement in* *Holliday & Witmer (2009) and Fernández et al. (2011)*.  0. yes  1. no |
| 121 | **Quadrate, fenestrae on the dorsal surface on the proximal end:**  0. two or more  1. none |
| 122 | **Quadrate, pneumatism:**  0. not pneumatic  1. pneumatic |
| 123 | **External auditory meatus fossa, anterior extention: (ORDERED) (RE-PHRASED)**  0. limited to the squamosal  1. reaches the posterior margin of postorbital  2. broadly exposed on the postorbital (covering the anterolateral margin)  3. crosses the postorbital and reaches the orbit |
| 124 | **Quadrate, squamosal, and otoccipital:**  0. do not meet to enclose cranioquadrate passage  1. enclose passage near lateral edge of skull  2. meet broadly lateral to the passage |
| 125 | **Cranioquadrate canal (NEW):**  *Character based on data from Jouve (2009).*  0. Incompletely separated from the external auditory aperature by a thin ventral lamina of the exoccipital not closed dorsally  1. Cranioquadrate canal clearly separated from the otic aperature by the quadrate or exoccipital and squamosal |
| 126 | **Eustachian tubes (ORDERED):**  *Character 121 from Nesbitt (2011); based on character 13 of Gower (2002)*.  0. not enclosed by bone  1. partially enclosed by bone  2. fully enclosed by bone |

Mandible

| Character | Description |
| --- | --- |
| 127 | **Mandible geometry, relative positions of the dentary tooth-row and coronid process, and development of dorsal curvature of the caudal-end of the mandible:**  *State (1) is an apomorphy of Metriorhynchidae. Quantifies the incipient increase of gape at the base of Metriorhynchidae.*  0. gentle curvature in the dorsal margin of the mandible, from the coronoid process to the end of the tooth-row  1. strong curvature, raising the coronoid process considerably above the tooth-row |
| 128 | **Mandible geometry, relative positions of coronoid process, retroarticular process and glenoid fossa:**  *State (1) is an apomorphy of the clade “Mr Leeds’ specimen + Geosaurini”. This character quantifies the greater increase in gape associated with macrophagous geosaurines.*  0. coronoid process level to both the retroarticular process and glenoid fossa  1. coronoid process ventral to both the retroarticular process and glenoid fossa |
| 129 | **Anterior of mandible (dentary), dorsal margin of the anterior portion compared to the dorsal margin of the posterior portion:**  *Character 154 in Nesbitt (2011)*  0. horizontal (in the same plane)  1. ventrally deflected  2. dorsally expanded |
| 130 | **Anterior of mandible (dentary), in dorsal view:**  *State (1) is an apomorphy of Teleosauridae.*  0. Outer margin converging towards tip or parallel  1. distinct notched spatulate shape |
| 131 | **Anterior of mandible (dentary), interalveolar space size (NEW):**  *State (1) is an apomorphy of Geosaurini, and is observed in* Suchodus durobrivensis  0. anterior interalveolar spaces are variable in size, ranging from being larger than the proceeding and preceding alveolus to being half the size  1. all anterior interalveolar spaces are less than half the length of the immediate alveoli |
| 132 | **Mandibular symphysis, length (character states altered) (ORDERED):**  0. symphysis less than a third of mandible length (lower than 0.3)  1. symphysis less than half and more than a third of mandible length (between 0.3 and 0.45)  2. symphysis under half of mandible length (between 0.45 and 0.5)  3. symphysis greater than half of mandible length (more than 0.5) |
| 133 | **Mandibular symphysis, depth (ORDERED):**  *This character quantifying the relative depth of the mandibular symphysis makes the absolute tooth crown size character redundant. This is because the metriorhynchids with the largest crowns also have the deepest symphyses (e.g.,* Dakosaurus andiniensis*,* Plesiosuchus manselii*).*  0. deep (10% or more of mandible length)  1. moderate (between 7-8% of mandible length)  2. narrow (between 4.5-6% of mandible length)  3. very narrow (4% or less of mandible length) |
| 134 | **External mandibular fenestra:**  *State (1) is an apomorphy of Metriorhynchidae.*  0. present  1. absent |
| 135 | **Surangulodentary groove, morphology: (NEW)**  *Note taphonomic or preservational damage can obscure state (1).*  *State (2) is an apomorphy of Geosaurini. Previously it was considered an apomorphy of* Dakosaurus*; however the type specimens for the genera* Dakosaurus, Plesiosuchus *and* Geosaurus *exhibit this morphology. The deep groove is also observed in a* Torvoneustes *specimen held in a private collection.*  0. absent  1. present as a subtle, shallow groove  2. deeply excavated |
| 136 | **Surangulodentary groove, extant: (NEW)**  0. groove is longer on the dentary than on the surangular  1. groove is as long on the dentary as on the surangular |
| 137 | **Surangulodentary groove, large foramen present at the dentary terminus:**  *State (1) is an apomorphy of* Dakosaurus.  0. absent  1. present |
| 138 | **Splenial, involvement in mandibular symphysis:**  0. slight (<10% of symphysis length)  1. extensive (>20% of symphysis length)  2. not involved |
| 139 | **Angular, in lateral view, length relative to the anterior margin of the orbit: (NEW)**  0. angular does not extend rostrally beyond the orbits  1. angular does extend rostrally beyond the orbits |
| 140 | **Surangular, in lateral view, length relative to the anterior margin of the orbit: (NEW)**  0. surangular does not extend rostrally beyond the orbits  1. surangular does extend rostrally beyond the orbits |
| 141 | **Surangular, along the dorsal margin of the mandible:**  *This character does not always covary with the character 120, as in non-Rhacheosaurini metriorhynchines the dentary extensively overlaps the surangular (particularly in lateral view), obscuring its rostral development. The full extent of the surangular rostral development can only be determined by examining the dorsal margin in those taxa (e.g.,* Metriorhynchus*).*  0. does not extend beyond the orbit  1. does extend beyond the orbit |
| 142 | **Surangular, possesses a distinct coronoid process:**  0. no  1. yes |
| 143 | **Surangular, extension toward posterior end of retroarticular process:**  0. along entire length  1. pinched off anterior to posterior tip |
| 144 | **Prearticular:**  0. present  1. absent |
| 145 | **Coronoid, rostral development along the dorsal margin:**  0. does not project as far as the dentary tooth row  1. projects further anteriorly than the caudal-most alveoli |
| 146 | **Coronoid, participates on the external face of the mandible:**  0. no  1. yes |
| 147 | **Articular, glenoid fossa orientation:**  0. anterodorsally  1. dorsally |
| 148 | **Retroarticular process, shape in dorsal view: (RE-PHRASED)**  0. broad and robust  1. triangular  2. paddle-shaped  3. ellipsoid, spoon-shaped |
| 149 | **Retroarticular process, width:**  0. narrower than the glenoid fossa  1. wider than the glenoid fossa (projecting medially past the glenoid fossa) |
| 150 | **Retroarticular process, length:**  0. long (longer than wide, and longer than the glenoid fossa width)  1. short (wider than long, and shorter than the glenoid fossa width) |

Dentition

| Character | Description |
| --- | --- |
| 151 | **Premaxilla tooth count (ORDERED):**  0. five  1. four  2. three |
| 152 | **Maxilla tooth count (ORDERED):**  0. 11, or fewer  1. 12-17  2. 18-20  3. 20-28  4. 28, or more |
| 153 | **Number of teeth partially supported by both the premaxilla and maxilla:**  0. none  1. one |
| 154 | **Dentary tooth count (ORDERED):**  *This character does not covary with the count of the maxillary teeth, as some taxa (e.g.* “Metriorhynchus” casamiquelai*) have more teeth in the dentary than in the maxilla*  0. 30, or more teeth per rami  1. 20-29  2. 19-14  3. 14, or fewer |
| 155 | **Premaxilla-anterior maxillary tooth crown apicobasal length to basal width ratio (NEW):**  0. 3 or greater  1. 2.5 or less |
| 156 | **Maxillary teeth, crown size (ORDERED):**  *Although this character would obviously correlate with the character quantifying mandibular symphysis depth, in Geosaurinae this is not necessarily the case. As shown by Young et al. (in press), the symphysis is deeper in* “Metriorhynchus” brachyrhynchus *than* “Mr Leeds’ Specimen”*, but the latter has tooth crowns with a greater apicobasal length.*  0. crowns not enlarged (typically less than 3cm in apicobasal length)  1. moderately enlarged (between 3 and 4 cm in apicobasal length)  2. enlarged (apicobasal length 6 cm or greater) |
| 157 | **Maxillary teeth, mediolateral compression/crown cross section: (character state removed)**  0. no mediolateral compression  1. weak mediolateral compression (crown midpoint labiolingual width 60-90% distal-medial width)  2. strong mediolateral compression (crown midpoint labiolingual width <60% distal-medial width) |
| 158 | **Maxillary teeth, crown cross section: (NEW)**  0. subcircular to elliptical  1. teardrop shaped |
| 159 | **Maxillary teeth, constriction at base of crown:**  0. absent  1. present |
| 160 | **Maxillary teeth, orientation of the anterior to mid-snout crowns:**  0. not procumbent  1. procumbent |
| 161 | **Maxillary teeth, enamel bands (sensu Brusatte *et al*., 2007):**  *Posterior-most maxillary crowns*  0. absent  1. present |
| 162 | **Maxillary teeth, tooth crown tip:**  *Anterior crowns*  0. sharpen or worn apex  1. blunt and rounded at the tips |
| 163 | **Dentary teeth posterior to tooth opposite premaxilla-maxilla contact:**  0. equal in size  1. enlarged dentary teeth opposite to smaller teeth in maxillary tooth row |
| 164 | **Teeth facets:**  *State (1) is an apomorphy of* Geosaurus giganteus *and* G. grandis.  0. either lacking, or faceted into 4-5 indistinct planes  1. distinctly faceted into 3 planes |
| 165 | **Laminar teeth (teeth with cross-section highly elliptical at base of crown, with mesial-distal axis approximately twice the labial-lingual axis, or greater):**  *State (1) is an apomorphy of* Geosaurus.  0. absent  1. present, laminar teeth dominate dentition |
| 166 | **Tooth curvature (ORDERED):**  0. none, crown apical/subapical (91 – 89 degrees)  1. weakly recurved (88 – 82 degrees)  2. strongly recurved (< 80 degrees) |
| 167 | **Tooth mesial and distal margins, presence of carinae:**  0. lack carinae  1. unicarinate – created by a smooth keel (raised ridge) on either the mesial or the disal margin  2. bicarinate – created by a smooth keel on both the mesial and distal margins |
| 168 | **‘Serrations’ created on the surface of the carinae by the conspicuous superficial ornamentation of enamel (false-ziphodonty, sensu Prasad & Broin, 2002):**  0. absent  1. present |
| 169 | **True denticles (true-ziphodonty, sensu Prasad & Broin, 2002) (NEW):**  *In Thalattosuchia, basal geosaurines (*“Mr Leeds’ specimen” *and* “Metriorhynchus” brachyrhynchus) *code as state (1).*  *Genera within Geosaurini codes as state (2).*  0. absent  1. incipient denticles that are poorly defined (hard to discern even under Scanning Electron Microscopy) and does not alter, or very little, the height of the carinal keel (definition described in Young et al., in press)  2. well defined denticles (can be discerned with or without optical aids) |
| 170 | **Carinae and true denticles, progression along the carinae (NEW):**  *In Thalattosuchia, basal geosaurines (*“Mr Leeds’ specimen” *and* “Metriorhynchus” brachyrhynchus) *code as state (1).*  *Genera within Geosaurini codes as state (2).*  *Note that this character and character describing possession of true denticles currently co-correlate. However, the two morphologies are not the same, and it is possible that taxa can code differently for these two characters (i.e. the ziphomorphy condition – see Andrade & Bertini, 2008).*  0. denticles are absent, or present on their own (i.e., they do not form a series)  1. heterogeneous carina, denticles form short rows (of 2-10 denticles) and do not proceed contiguously along the entire carina  2. homogeneous carina, denticles form a contiguous series along the entire carina |
| 171 | **Denticle shape, when observed in lingual or labial view (NEW):**  *In Thalattosuchia,* Plesiosuchus *codes as state (0).*  0. “chisel”-shaped or rectangular  1. rounded |
| 172 | **Denticle distribution across the dentition (NEW):**  Dakosaurus *codes as state (1).*  *At present no taxon is known to combine the microziphodont and macroziphodont conditions. However, it is entirely possible that such a taxon could occur. As such, state (3) was created.*  *In Thalattosuchia,* Dakosaurus *codes as (2), while “*Metriorhynchus*”* brachyrhynchus*,* “Mr Leeds’ specimen”*,* Torvoneustes*,* Geosaurus *and* Plesiosuchus *codes as (1).*  0. all or most teeth lack denticles  1. all teeth are microziphodont (sensu Andrade et al., 2010)  2. all teeth are macroziphodont (sensu Andrade et al., 2010)  3. teeth show variation in denticle size (with both microziphodonty and macroziphodonty) |
| 173 | **Occlusion, relation between maxillary and dentary series:**  0. in-line or interlocked  1. maxillary dentition overbites dentary dentition |
| 174 | **Morphology of enamel surface ornamentation, apicobasal ridges:**  *In Thalattosuchia,* Geosaurus*,* Dakosaurus, Rhacheosaurus *and* Cricosaurus *code as state (0).* “Mr Leeds’ specimen” *codes as state (1).* Plesiosuchus *codes as state (2).*  0. enamel ornamentation absent macroscopically, although under SEM microscopic ripples may be present  1. enamel ornamentation largely absent, with on the basal half of the crown short, well-spaced, well-defined apicobasally aligned ridges  2. composed of numerous apicobally aligned ridges that are of low-relief (can only be properly viewed with visual aids), set close to each other, but become shorter and well spaced towards the carinae  3. composed of numerous well-defined apicobasally aligned ridges, conspicuous and set close to each other |
| 175 | **Morphology of enamel surface ornamentation, macroscopic anastomosed pattern:**  0. absent  1. present and strongly developed, but only on the apical half of the crown |

**Axial skeleton**

| Character | Description |
| --- | --- |
| 176 | **Atlas, hypocentrum length:**  0. long: >15% of odontoid process length  1. short: subequal to odontoid process length (±5%) |
| 177 | **Axis, neural arch diapophysis:**  0. absent  1. present |
| 178 | **Presacral vertebrae number:**  0. 24  1. 25 |
| 179 | **Number of cervico-dorsal vertebrae where the parapophyses are borne on the centrum (‘cervical vertebrae’) (ORDERED):**  0. 9 or 10  1. 8  2. 7 |
| 180 | **Cervical vertebrae, shape:**  0. amphicoelous  1. procoelous |
| 181 | **Cervical vertebrae, centrum length vs centrum width (ORDERED):**  0. long (centrum, length >1.5 the centrum width)  1. moderate (centrum length - width subequal ±5%)  2. short (centrum length <0.95 the centrum width) |
| 182 | **Middle cervical vertebrae, neural spine height relative to centrum height (NEW) (ORDERED):**  *Currently, there is not the information to code most crocodylomorphs. Within Thalattosuchia* Steneosaurus edwardsi *is 0,* St. leedsi *codes as 1, and metriorhynchids as state 2.*  0. neural spine height is greater than centrum height  1. neural spine and centrum heights are approximately equal  2. neural spine height is less than centrum height |
| 183 | **Number of cervico-dorsal vertebrae where the parapophyses are borne on the neural arch (‘thoracic vertebrae’) (ORDERED):**  *This character, (along with character 184, categorising lumbral vertebrae) was formulated to help understand the regionalisation of the presacral column. Currently, there is not the information to code most crocodylomorphs.*  0. 12  1. 13  2. 14  3. 15 |
| 184 | **Number of cervico-dorsal vertebrae posterior to the “thoracic vertebrae” and anterior to the sacral vertebrae where the parapophyses are no longer borne on the neural arch (‘lumbral vertebrae’) (ORDERED):**  *This character, (along with character 183, categorising thoracic vertebrae) was formulated to help understand the regionalisation of the presacral column. Currently, there is not the information to code most crocodylomorphs.*  0. 2  1. 3  2. 4 |
| 185 | **Thoracic and lumbral vertebrae, shape:**  0. amphicoelous  1. procoelous |
| 186 | **Thoracic vertebrae, shallow fossa on the anterior margin of the diapopohysis immediately lateral to the parapophysis:**  *State( 0) is a apomorphy of metriorhynchids, best observed in thoracic vertebrae mid-to-late in the series*  0. present  1. absent |
| 187 | **Thoracic vertebrae, orientation of parapophysis:**  *State (1) is an apomorphy of Metriorhynchidae.*  0. posteriorly or horizontal  1. anteriorly |
| 188 | **Anterior thoracic vertebrae, parapophysis in relation to the diapophysis (NEW):**  *Currently, there is not the information to code most crocodylomorphs. Within Thalattosuchia* Steneosaurus edwardsi *and* St. leedsi *are state 0, and metriorhynchids code as state 1.*  0. parapophysis ventral to, or level with, diapophysis (when observed in lateral view)  1. parapophysis dorsal to diapophysis (when observed in lateral view) |
| 189 | **Anterior thoracic vertebrae, neural spine height relative to centrum height (NEW):**  *Currently, there is not the information to code most crocodylomorphs. Within Thalattosuchia* Machimosaurus mosae *and* Steneosaurus edwardsi *are 0, and* St. leedsi *and metriorhynchids code as state 1.*  0. neural spine and centrum heights are approximately equal  1. neural spine height is less than centrum height |
| 190 | **‘‘Insertion’’ of a sacral vertebra between the first and second primordial sacral vertebrae:**  *Character 207 from Nesbitt (2011).*  *This character codes for the “third” sacral found in certain taxa (e.g.* Machimosaurus*). Within Thalattosuchia, three sacral vertebrae is an apomorphy of* Machimosaurus*.*  0. absent  1. present |
| 191 | **Caudal vertebrae, number:**  0. less than 46  1. more than 50 |
| 192 | **Tail, vertebrae morphology near distal end:**  *State (1) is an apomorphy of Metriorhynchidae. Character re-phrased based on Andrade (2010).*  0. non-hypocercal, distal vertebrae isomorphic to poorly heteromorphic  1. hypocercal, caudal series clearly heteromorphic, with a section of the distal vertebrae defining the lower lobe of a tail fin |
| 193 | **Axis rib (NEW):**  *State (1) is an apomorphy of* Pelagosaurus *and Metriorhynchidae. Callovian teleosaurids have a distinct ‘bump’ or ‘process’ where a second articular head would be (see Andrews, 1913). However, in no specimen is there a second articular head preserved.*  0. holocephalous (rib elongate, with one articular head)  1. dichocephalous (rib triradiate, with two articular heads) |
| 194 | **Axis rib, tuberculum:**  0. wide with broad dorsal tip  1. narrow with acute dorsal tip |
| 195 | **Sacral vertebrae, orientation of the transverse processes:**  *State (1) is an apomorphy of Thalattosuchia.*  0. horizontal  1. arched ventrally, at least in the first sacral |
| 196 | **Sacral vertebrae, relative position of lateral end of the transverse processes:**  *State (1) is an apomorphy of* Pelagosaurus *and Metriorhynchidae.*  0. level with the vertebral centrum  1. ventral relative to the vertebral centrum, transverse processes of both sacrals lateroventrally directed |
| 197 | **Chevrons (=haemal arches), shape (posterior chevrons have a anterodorsal process):**  *State (1) is an apomorphy of Metriorhynchidae.*  0. either ‘V’ or ‘Y’-shaped, no distinct anterodorsal process  1. posterior chevrons have a ‘W’-shape when observed in anterior view, formed by a anterodorsal process rising between the ‘Y’-shape |

**Appendicular skeleton**

| Character | Description |
| --- | --- |
| 198 | **Coracoid, shape (ORDERED):**  0. neither proximal nor distal end are fan-shaped, having angular margins  1. distal end convex, forming a gentle fan-shape while the proximal (scapula-articular) end is triangular in shape with blunt ends  2. both proximal and distal ends are convex |
| 199 | **Scapula blade (NEW):**  *State (1) is an apomorphy of Thalattosuchia, except* Platysuchus multiscrobiculatus.  0. scapula blade very large, more than 200% of the width of the scapular shaft, while the scapular shaft contributes less than 50% of total scapular length  1. scapula blade reduced, being narrower than the proximal region and less than 150% the width of the scapular shaft, while the scapular shaft contributes at least 50% of total scapula length |
| 200 | **Scapula, anterior and posterior margins in lateral aspect:**  *New character state*  0. symmetrically concave in lateral view  1. anterior edge more strongly concave than posterior edge  2. posterior edge more strongly concave than anterior edge |
| 201 | **Scapula, deltoid crest:**  0. present  1. absent |
| 202 | **Scapula/Humerus, size (ORDERED):**  0. humerus longer than scapula (> 15%)  1. humerus and scapula subequal in length (± 13%)  2. humerus shorter in length than scapula (< 15%) |
| 203 | **Humerus, proximal head:**  *Character 232 from Nesbitt (2011):*  0. confined to the proximal surface  1. posteriorly expanded and hooked |
| 204 | **Humerus, proximomedial articular surface:**  0. present (strongly arched edge)  1. absent (weakly arched edge) |
| 205 | **Humerus, deltopectoral crest:**  *Sstate (2) (absent/vestigial) has been removed, as metriorhynchids of the subclade Rhacheosaurini do indeed possess a deltopectoral crest*  0. present and distinct from the proximal surface  1. present, but continuous with the proximal surface |
| 206 | **Humerus, shape: (NEW)**  *State (1) is an apomorphy of Metriorhynchidae*  0. has typical long bone morphology (longer than width at distal end)  1. broadly expanded and plate-like |
| 207 | **Humerus, length of the shaft relative to total humerus length: (character re-phrased)**  *This character quantifies the reduction in humeral shaft size in Metriorhynchidae*  0. shaft contributing more than 50% of total humeral length  1. shaft contributes between 35-38% of total humeral length  2. shaft contributes less than 25% of total humeral length |
| 208 | **Humerus-antebrachium joint surface:**  *State (1) is an apomorphy of Metriorhynchidae*  0. complex, allowing one degree of motion  1. planar, limiting possible motion |
| 209 | **Radius, shape:**  *State (1) is an apomorphy of Metriorhynchinae. The geosaurine condition is unknown*  0. has typical long bone morphology (longer than width at distal end)  1. broadly expanded and plate-like |
| 210 | **Radiale, shape:**  *State (1) is an apomorphy of Metriorhynchinae. The geosaurine condition is unknown*  0. has typical long bone morphology (longer than width at distal end)  1. broadly expanded and plate-like |
| 211 | **Ulna, shape:**  *State (1) is an apomorphy of Metriorhynchinae. The geosaurine condition is unknown*  0. has typical long bone morphology (longer than width at distal end)  1. broadly expanded and plate-like |
| 212 | **Ulnare, shape:**  *State (1) is an apomorphy of Metriorhynchinae. The geosaurine condition is unknown*  0. has typical long bone morphology (longer than width at distal end)  1. broadly expanded and plate-like |
| 213 | **Metacarpal 1, shape:**  *State (1) is an apomorphy of Metriorhynchinae. The geosaurine condition is unknown*  0. elongate, more than twice long as wide  1. broadly expanded, maximum width at least 60% total length |
| 214 | **Pubis, contribution to acetabulum (ORDERED):**  *Character 86 from Turner & Sertich (2010).*  0. forms anterior half of ventral edge of acetabulum  1. contacting ilium but partially excluded from acetabulum by anterior process of ischium  2. completely excluded from acetabulum by anterior process of ischium |
| 215 | **Ilium, posterior process presence:**  *Character 128 in Young & Andrade (2009), Andrade et al. (2010), Young et al. (2011a).*  0. present  1. absent |
| 216 | **Ilium, posterior margin expanded into a thin “fan”-shape (NEW):**  *State (1) is an apomorphy of Teleosauridae (except* Platysuchus multiscrobiculatus *and* Steneosaurus bollensis*).*  0. no  1.yes, posterior margin is expanded into a thin “fan”-shape that extends from the iliac crest to the ventral margin |
| 217 | **Illium, size:**  0. large (length of dorsal border at least 30% of femur length)  1. small (length of dorsal border less than 21% of femur length) |
| 218 | **Ischium, anterior process (ORDERED):**  0. developed – with clearly defined articulation facets for pubis and ilium; additionally anterior process is at least half as wide as the posterior process  1. reduced – lacks both articulation facets, and is 30-50% as wide as posterior process  2. highly reduced – lacking both articulation facets, and is < 25% as wide as posterior process |
| 219 | **Femur, proximal portion, posteromedial tuber:**  *Character 301 from Nesbitt (2011), character states re-ordered.*  0. absent  1. present and small  2. present and largest of the proximal tubera |
| 220 | **Femur, proximal condylar fold:**  *Character 312 from Nesbitt (2011).*  0. absent  1. present |
| 221 | **Femur, ridge of attachment of the M. caudifemoralis:**  *Modified from character 315 in Nesbitt (2011).*  *Note: we code thalattosuchians as state (0). They lack a fourth trochanter sensu stricto, as they only possess a large flattened rugose area for the muscle attachment, not a distinct process.*  0. absent, flattened rugose area  1. low and without a distinct medial asymmetrical apex (= fourth trochanter)  2. bladelike with a distinct asymmetric apex located medially |
| 222 | **Lateral edge of proximal articular surface of femur (lesser trochanter):**  0. rounded  1. ‘squared’ with enlarged scar for musculus ischiotrochantericus |
| 223 | **Femur, medial condyle of the distal portion:**  *Character 320 from Nesbitt (2011).*  0. tapers to a point on the medial portion in distal view  1. smoothly rounded in distal view |
| 224 | **Femur, distal surface between the lateral and medial condyles:**  *Character 321 from Nesbitt (2011).*  0. nearly flat or flat  1. groove separating the medial condyle from the lateral condyle |
| 225 | **Tibia, length (ORDERED):**  0. long (>45% of femur length)  1. medium (40-45% of femur length)  2. short (31-39% of femur length)  3. very short (< 30% of femur length) |
| 226 | **Calcaneum tuber:**  0. well developed – with long neck (subequal in length to main body of calcaneum ±5%), distal end wider than main body of calcaneum and projects inwards the body at >80 degress  1. poorly developed – short neck (< half length of calcaneum main body), distal end < half the width of calcaneum main body width & projects out straight from calcaneum  2. absent/vestigial – no defined tuber, the posterior edge of calcaneum one smooth, gentle curve |
| 227 | **Metatarsals, length:**  0. metatarsals 1-4 longer than digits (>20%)  1. metatarsals 2-4 shorter than digits (< 90%) |
| 228 | **Metatarsal I, proximal end expansion (ORDERED):**  0. proximal end not enlarged (no more than 10% wider than any other metatarsal)  1. enlarged (20-30% wider)  2. moderately enlarged (46-51%)  3. greatly enlarged (>75% wider) |
| 229 | **Pes, digit lengths:**  0. digit lengths in descending order III, IV, II, I  1. IV, III, II, I (digit IV elongated, creating a paddle-like shape as each digit is ~10% shorter) |
| 230 | **Forelimb – hind limb, length ratio (ORDERED):**  *Character re-designed, based on Character 212 of Nesbitt (2011), number of character states expanded to reflect the forelimb reduction in Thalattosuchia*  *= humerus + radius : femur + tibia*  0. less than 0.45  1. between 0.45 and 0.55  2. greater than 0.55 |
| 231 | **Hind limb, distal to proximal bone length ratio (NEW) (ORDERED):**  *This character is designed to help elucidate variation in the proportions of the hind limb. In Thalattosuchia state (0) is an apomorphy of both Metriorhynchinae and* Steneosaurus priscus*, while Middle Jurassic teleosaurids (and the Late Jurassic genus* Machimosaurus*) and Geosaurinae code as state (1).*  *= tibia : femur*  0. less than 0.4  1. between 0.4 and 0.5  2. greater than 0.5 |

Dermal Armour

| Character | Description |
| --- | --- |
| 232 | **Dorsal osteoderm distribution:**  0. absent  1. present in two parallel rows (paravertebral rows)  2. present with more than two parallel rows (paravertebral and accessory rows) |
| 233 | **Dorsal osteoderm, presence of a ‘peg-like’ anterolateral process (forming a stylofoveal joint):**  0. absent  1. present |
| 234 | **Dorsal osteoderm, dimensions (NEW):**  *Based on character 407 in Nesbitt (2011)*  *Croc-line archosaurs including basal crocodylomorphs have state (1)*  *Crocodyliformes have state (2)*  0. square shaped, length and width approximately equal  1. longer than wide  2. wider than long |
| 235 | **Dorsal osteoderms, paravertebral only (NEW):**  *Based on character 404 in Nesbitt (2011)*  *Croc-line archosaurs including basal crocodylomorphs have state (1)*  *Crocodyliformes have state (0)*  0. flat or weakly arched  1. distinct longitudinal bend near lateral edge |
| 236 | **Ventral trunk osteoderm distribution:**  0. present  1. absent |
| 237 | **Dorsal tail osteoderm distribution (ORDERED):**  *Character previously coded for both the ventral and dorsal caudal rows together. This character was split as* Pelagosaurus *lacks ventral caudal osteoderms, but possesses dorsal caudal osteoderms.*  0. present, covering at least half of the tail  1. present, covering less than half of the tail  2. absent |
| 238 | **Ventral tail osteoderm distribution (NEW):**  *State (1) is an apomorphy of* Pelagosaurus *+ Metriorhynchidae*  0. present  1. absent |
| 239 | **Osteoderm dorsal surface, ornamentation:**  *State (2) is an apomorphy of* Machimosaurus  0. small round to ellipsoid pits in very dense equal distribution  1. large round to ellipsoid pits but in high density  2. pits variable in size and length, from small to large, but on osteoderms with a keel, the pits can become elongate grooves |
| 240 | **Osteoderm dorsal surface, keel (longitudinal ridge) (NEW):**  0. present along the entire series of the paravertebral osteoderms (i.e., cervical to caudal)  1. reduced distribution on the paravertebral osteoderms (lumbral, sacral and caudal osteoderms)  2. keel absent on all paravertebral osteoderms |

**3) Supplementary references**

Andrade MB (2005) Revisão sistemática e taxonômica dos Notosuchia (Metasuchia, Crocodylomorpha). Unpublished MSc. Thesis, Universidade Estadual Paulista, Rio Claro, Brazil, 239p.

Andrade MB, Bertini RJ (2008) A new *Sphagesaurus* (Mesoeucrocodylia: Notosuchia) from the Upper Cretaceous of Monte Alto City (Bauru Group, Brazil), and a revision of the Sphagesauridae. Historical Biology 20: 101-136.

Bonaparte JF (1991) Los vertebrados fosiles de la Formacion Rio Colorado, de la Ciudad de Neuquen y cercanias, Cretacico Superior, Argentina. Revista del Museo Argentino de Ciencias Naturales 4: 31-63.

Buchy M-C, Vignaud P, Frey E, Stinnesbeck W, González AHG (2006) A new thalattosuchian crocodyliform from the Tithonian (Upper Jurassic) of northeastern Mexico. Comptes Rendus Palevol 5: 785–794.

Buckley GA, Brochu CA (1999) An enigmatic new crocodile from the Upper Cretaceous of Madagascar. In: Unwin D. eds, Cretaceous Fossil Vertebrates: Special Papers in Palaeontology 60: 149–175. London: The Palaeontological Association.

Buffetaut E (1977) Sur un crocodilien marin *Metriorhynchus superciliosus*, de l’Oxfordien supérieur (Rauracien) de l’Ile de Ré (Charente-Maritime). Annales de la Société des Sciences Naturelles de la Charente-Maritime 4: 252–266.

Carvalho IS, Bertini RJ (1999) *Mariliasuchus*: um novo Crocodylomorpha (Notosuchia) do Cretáceo da Bacia Bauru, Brasil. Geologia Colombiana 24: 83-105.

Carvalho IS, Campos ACA, Nobre PH (2005) *Baurusuchus salgadoensis*, a new Crocodylomorpha from Bauru Basin (Cretaceous), Brazil. Gondwana Research 8: 11-30.

Carvalho IS, Ribeiro LCB, Avilla LS (2004) *Uberabasuchus terrificus* sp. nov., a new Crocodylomorpha from the Bauru Basin (Upper Cretaceous), Brazil. Gondwana Research 7: 975-1002.

Carvalho IS, Vasconcellos FM, Tavares SA-CS (2007) *Montealtosuchus arrudacamposi*, a new peirosaurid (Mesoeucrocodylia) from the Late Cretaceous Adamantina Formation of Brazil. Zootaxa 1607: 35-46.

Clark JM, Sues H-D, Berman DS (2000) A new specimen of *Hesperosuchus agilis* from the Upper Triassic of New Mexico and the interrelationships of basal crocodylomorph archosaurs. Journal of Vertebrate Paleontology 20: 683–704.

Colbert EC, Mook CC (1951) The ancestral crocodile *Protosuchus*. Bulletin of the American Museum of Natural History 97: 143–182.

Crush PJ (1984) A late Upper Triassic sphenosuchid crocodilian from Wales. Palaeontology 27: 131-157.

Debelmas J (1958) Découverte d’une ceinture pelvienne de dacosaure dans le néocomien des environs de Castellane (Basses-Alpes). Travaux du Laboratoire de Géologie de la Faculté des Sciences de l’Université de Grenoble 34: 43–48.

Frey E, Buchy M-C, Stinnesbeck W, López-Oliva JG (2002) *Geosaurus* *vignaudi* n. sp. (Crocodylia, Thalattosuchia), first evidence of metriorhynchid crocodilians in the Late Jurassic (Tithonian) of central-east Mexico (State of Puebla). Canadian Journal of Earth Sciences 39: 1467–1483.

Gasparini ZB (1973) Revisión de ‘? *Purranisaurus* *potens’* Rusconi, 1948 (Crocodila, Thalattosuchia). Los Thalattosuchia como un nuevo infraorden de los Crocodilia. Actas del congreso Geológica Argentino 3: 423–431.

## Gasparini ZB, Cichowolski M, Lazo DG (2005) First record of Metriorhynchus (Reptilia: Crocodyliformes) in the Bathonian (Middle Jurassic) of the Eastern Pacific. **Journal of Paleontology** 79: 801-805.

Gasparini ZB, Chong G (1977) *Metriorhynchus casamiquelai* n. sp. (Crocodilia, Thalattosuchia) a marine crocodile from the Jurassic (Callovian) of Chile, South America. Neues Jahrbuch für Geologie und Paläontologie, Abhandlungen 153: 341–360.

Gasparini ZB, Iturralde-Vinent M (2001) Metriorhynchid crocodiles (Crocodyliformes) from the Oxfordian of Western Cuba. Neues Jahrbuch für Geologie und Paläontologie, Monatshefte 9: 534–542.

Gasparini ZB, Vignaud P, Chong G (2000) The Jurassic Thalattosuchia (Crocodyliformes) of Chile: a paleobiogeographic approach. Bulletin de la Société Géologique de France 171: 657–664.

Gow CE (2000) The skull of *Protosuchus haughtoni*, an Early Jurassic crocodyliform from Southern Africa. Journal of Vertebrate Paleontology 20: 49–56.

Gower DJ (2002) Braincase evolution in suchian archosaurs (Reptilia: Diapsida): evidence from the rauisuchian *Batrachotomus kupferzellensis*. Zoological Journal of the Linnean Society 136: 49–76.

Herrera Y, Fernández MS, Varela JA (2009) Morfología del miembro anterior de *Geosaurus* *araucanensis* Gasparini y Dellapé, 1976 (Crocodyliformes: Thalattosuchia). Ameghiniana 46: 657–667.

Hua S, Vignaud P, Atrops F, Clément A (2000) *Enaliosuchus macrospondylus* Koken, 1883 (Crocodylia, Metriorhynchidae) du Valanginien de Barret-le-Bas (Hautes Alpes, France): un cas unique de remontée des narines externs parmi les crocodiliens. Géobios 33: 467–474.

Jouve S (2009) The skull of *Teleosaurus cadomensis* (Crocodylomorpha; Thalattosuchia), and phylogenetic analysis of Thalattosuchia. Journal of Vertebrate Paleontology 29: 88-102.

Larsson HCE, Sues H-D (2007) Cranial osteology and phylogenetic relationships of *Hamadasuchus rebouli* (Crocodyliformes: Mesoeucrocodylia) from the Cretaceous of Morocco. Zoological Journal of the Linnean Society 149: 533–567.

Lennier G (1887) Description des fossils du Cap de la Heve. Bulletin de la Société Géologique de Normandie, Le Harve 12: 17-98.

Mercier J (1933) Contribution à l’étude des Métriorhynchidés (crocodiliens). Annales de Paléontologie 22: 99-119.

Nobre PH, Carvalho IS (2006) *Admantinasuchus navae*: a new Gondwanan Crocodylomorpha (Mesoeucrocodylia) from the Late Cretaceous of Brazil. Gondwana Research 10: 370-378.

Pol D (2003) New remains of *Sphagesaurus huenei* (Crocodylomorpha: Mesoeucrocodylia) from the Late Cretaceous of Brazil. Journal of Vertebrate Paleontology 23: 817-831.

Price LI (1945) A new reptile from the Cretaceous of Brazil. Notas Preliminares e Estudos - DGM 25: 1-8.

Price LI (1950) On a new crocodilian, *Sphagesaurus*, from the Cretaceous of the State of São Paulo, Brazil. Anais da Academia Brasileira de Ciências 22: 77-85.

Rusconi C (1948) Nuevo plesiosaurio pez y Langostas del mar Jurassico de Mendoza. Revista del Museo de Historia Natural de Mendoza 2: 3-12.

Rusconi C (1955) Acerca del plesiosaurio ‘*Purranisaurus*’ del Jurasico de Mendoza. Anales de la Sociedad Científica Argentina 160: 71–77.

Salisbury SW, Frey E, Martill DM, Buchy M-C (2003) A new crocodilian from the Lower Cretaceous Crato Formation of north-eastern Brazil. Palaeontographica Abteilung A 270: 3-47.

Salisbury SW, Molnar RE, Frey E, Willis P (2006) The origin of modern crocodyliforms: new evidence from the Cretaceous of Australia. Proceedings of the Royal Society B 273: 2439–2448.

Schwarz D, Salisbury SW (2005) A new species of *Theriosuchus* (Atoposauridae, Crocodylomorpha) from the Late Jurassic (Kimmeridgian) of Guimarota, Portugal. Geobios 38: 779-802.

Sues H-D, Olsen PE, Carter JG, Scott DM (2003) A new crocodylomorph archosaur from the Upper Triassic of North Carolina. Journal of Vertebrate Paleontology 23: 329-343.

Turner AH, Buckley GA (2008) *Mahajangasuchus insignis* (Crocodyliformes: Mesoeucrocodylia) cranial anatomy and new data on the origin of the eusuchian-style palate. Journal of Vertebrate Paleontology 28: 382-408.

Turner AH, Sertich JJW (2010) Phylogenetic history of *Simosuchus clarki* (Crocodyliformes: Notosuchia) from the Late Cretaceous of Madagascar. Journal of Vertebrate Paleontology 30, supplement to issue 6: 177- 236. Society of Vertebrate Paleontology Memoir 10.

Tykoski RS, Rowe TB, Ketcham RA, Colbert MW (2002) *Calsoyasuchus valliceps*, a new crocodyliform from the Early Jurassic Kayenta Formation of Arizona. Journal of Vertebrate Paleontology 22: 593–611.

Vasconcellos FM, Carvalho IS (2005) Estágios de desenvolvimento de *Mariliasuchus amarali*, Crocodyliformes Mesoeucrocodylia da Formação Adamantina, Cretáceo Superior da Bacia Bauru, Brasil. Anuário do Instituto de Geociências 28: 49-69.

Weinbaum JC (2011) The skull of *Postosuchus kirkpatricki* (Archosauria: Paracrocodyliformes) from the Upper Triassic of the United States. PaleoBios 30: 18–44

Woodward AS (1896) On two Mesozoic crocodilians, *Notosuchus* (genus novum) and *Cynodontosuchus* (genum novum) from the red sandstones of the Territory of Neuquén (Argentine Republic). Anales del Museo de La Plata – Serie Paleontología Argentina 4: 1-20.

**4) Complete list of institutional abbreviations**

**AMNH**, American Museum of Natural History, New York City, NY, USA

**BRSMG**, Bristol City Museum & Art Gallery, Bristol, England, UK

**BSPG**, Bayerische Staatssammlung für Paläontologie und Historische Geologie, München, Germany

**CAMSM**, Sedgwick Museum, Cambridge, England, UK

**CM**, Carnegie Museum of Natural History, Pittsburgh, PA, USA

**CMC**, Cincinnati Museum Center, Cincinnati, OH, USA

**DGM**, Departamento Nacional de Produção Mineral, Rio de Janeiro, Brazil

**FEF**, Fundação Educacional de Fernandópolis, Fernandópolis, Brazil

**GLAHM**, Hunterian Museum, Glasgow, Scotland, UK

**HMN**, Humboldt Museum für Naturkunde, Berlin, Germany

**MACN**, Museo Argentino de Ciências Naturales ‘Bernardino Rivadavia’, Buenos Aires, Argentina

**MLP**, Museo de La Plata, La Plata, Argentina

**MPMA**, Museu de Paleontologia de Monte Alto, Monte Alto, Brazil

**MN-UFRJ**, Museu Nacional, Universidade Federal do Rio de Janeiro, Rio de Janeiro, Brazil

**MUCPv**, Museo de la Universidad Nacional del Comahue, Neuquén, Argentina

**NHMUK**, Natural History Museum, London, England, UK

**OXFUM**, Oxford University Museum of Natural History, Oxford, England, UK

**PETMG**, Peterborough Museum & Art Gallery, Peterborough, England, UK

**RCL**, Museu de Ciências Naturais da Pontifícia Universidade Católica de Minas Gerais, Belo Horizonte, Brazil

**RMS**, Royal Museum Scotland, Edinburgh, Scotland, UK

**SMNK**, Staatliches Museum für Naturkunde Karlsruhe, Germany

**SMNS**, Staatliches Museum für Naturkunde Stuttgart, Germany

**UFRJ-DG**, Departamento de Geologia, Universidade Federal do Rio de Janeiro, Brazil

**URC, IGCE-UNESP**, Museu “Paulo Milton Barbosa Landim”, Instituto de Geociências e Ciências Exatas, Universidade Estadual Paulista, Rio Claro, Brazil

**USNM**, National Museum of Natural History, Washington DC, USA
